# *Limosilactobacillus mucosae*-derived extracellular vesicles modulates macrophage phenotype and orchestrates gut homeostasis in a diarrheal piglet model

**DOI:** 10.1038/s41522-023-00403-6

**Published:** 2023-06-06

**Authors:** Jingjing Li, Shuaifei Feng, Zhenyu Wang, Jinhui He, Zeyue Zhang, Huicong Zou, Zhifeng Wu, Xiangdong Liu, Hong Wei, Shiyu Tao

**Affiliations:** 1 College of Animal Sciences and Technology, Huazhong Agricultural University, Wuhan, 430070 China; 2 State Key Laboratory of Animal Nutrition, College of Animal Science and Technology, China Agricultural University, No. 2 Yuanmingyuan West Road, Beijing, 100193 China

**Keywords:** Metagenomics, Applied microbiology

## Abstract

The diarrheal disease causes high mortality, especially in children and young animals. The gut microbiome is strongly associated with diarrheal disease, and some specific strains of bacteria have demonstrated antidiarrheal effects. However, the antidiarrheal mechanisms of probiotic strains have not been elucidated. Here, we used neonatal piglets as a translational model and found that gut microbiota dysbiosis observed in diarrheal piglets was mainly characterized by a deficiency of *Lactobacillus*, an abundance of *Escherichia coli*, and enriched lipopolysaccharide biosynthesis. *Limosilactobacillus mucosae* and *Limosilactobacillus reuteri* were a signature bacterium that differentiated healthy and diarrheal piglets. Germ-free (GF) mice transplanted with fecal microbiota from diarrheal piglets reproduced diarrheal disease symptoms. Administration of *Limosilactobacillus mucosae* but not *Limosilactobacillus reuteri* alleviated diarrheal disease symptoms induced by fecal microbiota of diarrheal piglets and by ETEC K88 challenge. Notably, *Limosilactobacillus mucosae*-derived extracellular vesicles alleviated diarrheal disease symptoms caused by ETEC K88 by regulating macrophage phenotypes. Macrophage elimination experiments demonstrated that the extracellular vesicles alleviated diarrheal disease symptoms in a macrophage-dependent manner. Our findings provide insights into the pathogenesis of diarrheal disease from the perspective of intestinal microbiota and the development of probiotic-based antidiarrheal therapeutic strategies.

## Introduction

Diarrheal disease is common in infants, with more than 500,000 deaths from diarrheal disease reported each year worldwide in children under 5 years of age^1–3^. In addition to its high mortality, diarrheal disease in early childhood causes malnutrition and persistent intestinal damage^4–6^. The diarrheal disease leads to impaired intestinal barrier function and increased intestinal mucosal permeability, inducing the onset of intestinal inflammation and, ultimately, growth retardation, as well as reduced resistance to subsequent pathogen infections^7,8^. Although the mechanisms leading to diarrhea have been studied for decades and diarrheal mortality has declined substantially, diarrhea continues to cause significant human mortality and morbidity, mainly because of its complex causes^9,10^. Therefore, elucidating the pathogenesis of neonatal diarrheal diseases is of great importance.

Early life is a window of opportunity for the development of the intestinal microbiota, intestinal physical barrier function, and the immune system^11^. The gut microbiota of the neonate has profound impacts on host health by regulating intestinal nutrient metabolism, maturation of the immune system, and maintenance of the intestinal barrier^12–15^. The instability of the gut microbiota and the immaturity of the immune system make newborns particularly vulnerable to pathogens^16^. When diarrhea occurs, a marked increase in the colonization of pathogenic microorganisms follows the disruption of the composition of the intestinal microbiota, suggesting that disruptions of the microbiota enhance the pathogenicity of these strains^17^. A stable intestinal microbiota promotes host health by improving resistance to colonizing by pathogenic microorganisms^18,19^.

Recently, fecal microbiota transplantation (FMT) achieved unexpectedly positive results in the treatment of *Clostridium difficile* infection, inflammatory bowel disease, and irritable bowel syndrome; therefore, FMT has become a routine therapy for complex intestinal diseases^20–23^. However, it has been a challenge to clarify which intestinal microorganisms confer the therapeutic effect during FMT treatment. Growing evidence suggests that reconstitution of gut microbiota with keystone probiotic strains is an effective strategy against gastrointestinal diseases^24,25^. Thus, the identification of potential specific probiotics with diarrhea-relieving potential may provide new strategies for the precise control of neonatal diarrheal disease and the prevention of intestinal diseases.

Bacterial extracellular vesicles (EVs) participate in a wide variety of pathophysiological functions that involve intercellular interactions, such as nutrient acquisition, virulence factor delivery, and immune regulation^26,27^. In particular, EVs have been shown to mediate bacterial pathogenesis and invasion through the delivery of toxins and virulence factors to host cells^28^. Much of the early work on bacterial EVs focused on their roles in pathogenic Gram-negative bacteria, but an increasing number of Gram-positive bacteria have recently been shown to produce EVs. For example, EV-producing Gram-positive bacteria include *Bacillus anthracis*^29^, *Streptococcus pneumonia*e^30^, *B. subtilis*^31^, and *C. perfringens*^32^. *Lactobacillus* is the most common Gram-positive bacterium, and several *Lactobacillus* species have recently been found to produce EVs, such as *L. casei BL23*^33^, *L. plantarum WCFS1*^34^, and *L. acidophilus*^35^. However, the potential roles and functions of these *Lactobacillus*-derived EVs have not been extensively studied, even though a recent study has shown that some *Lactobacillus* strains have superior efficacy in preventing diarrheal disease in mammals^24^. Therefore, whether *Lactobacillus* with antidiarrheal properties exerts their effects through their EVs deserves further exploration.

The similarities in intestinal structure and development between humans and pigs make pigs a superior animal model for studying the pathophysiological basis of gastrointestinal diseases^36,37^. Here, using neonatal piglets, germ-free (GF) mice, and specified pathogen-free (SPF) mice as animal models and by integrating multi-omics, fecal microbial transplantation (FMT), enterotoxigenic *Escherichia coli* K88 (ETEC K88)-induced intestinal inflammatory damage, and interventions with *Limosilactobacillus mucosae* (*L. mucosae*) and its derived EVs, this study systematically characterized the intestinal microbiome of newborn diarrheal piglets, further revealed the causal relationship between intestinal microbiota and diarrheal disease symptoms, and elucidated the underlying mechanism by which *Lactobacillus*-derived from healthy piglets reduces diarrheal disease symptoms. These results provide insights into how *Lactobacilli* and its derived EVs affect infant gut health and suggest strategies for the prevention and treatment of diarrhea-related diseases.

## Results

### Differences in gut microbiota between healthy and diarrheal piglets

**Fig. 1:**
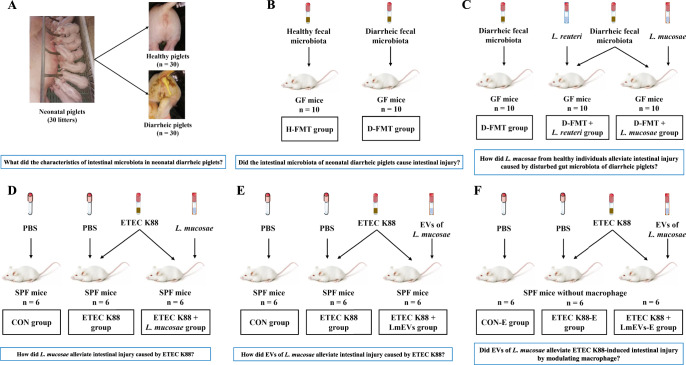
Study design for the entire experiment. **A** Selection of healthy newborn and diarrheal piglets. **B** Transplantation of fecal microbiota from healthy and diarrheic piglets to GF mice. **C** The protective effect of *L. mucosae* and *L. reuteri* on intestinal damage caused by fecal microbiota in diarrheal piglets. **D** The protective effect of *L. mucosae* on intestinal damage caused by ETEC K88. **E** The protective effect of EVs of *L. mucosae* on intestinal damage caused by ETEC K88. **F** In vivo elimination of macrophages in mice.

To reveal the characteristics of intestinal microbiota composition and function in healthy and diarrheal newborn piglets, we performed metagenomic sequencing of fecal samples collected from 30 healthy and 30 diarrheal newborn piglets (Fig. 1A). At the phylum level, the Richness index was significantly lower (*P* = 0.035) while the Shannon (*P* = 0.043) and Simpson (*P* = 0.022) indexes were significantly higher in diarrheal piglets as compared to healthy piglets (Supplementary Fig. 1A). A principal coordinates analysis (PCoA) showed that the microbial structure also differed between the two groups (*P* = 0.002; Supplementary Fig. 1B). Firmicutes, Bacteroidetes, and Proteobacteria were the dominant phylum in both groups (Supplementary Fig. 1C). The proportions of Firmicutes and Actinobacteria were significantly lower in diarrheal piglets, while the proportion of Fusobacteria was significantly higher (Supplementary Fig. 1D).

At the genus level, the Richness index was significantly lower (*P* = 0.000) and the Simpson (*P* = 0.018) index was significantly higher in diarrheal newborn piglets (Supplementary Fig. 2A). There were also significant differences in the structure of the microbiota at the level of the genera between the two groups (*P* = 0.000; Supplementary Fig. 2B). *Lactobacillus*, *Escherichia*, and *Bacteroides* were the dominant genera in diarrheal newborn piglets, while *Lactobacillus*, *Bacteroides*, and *Prevotella* were the dominant genera in healthy newborn piglets (Supplementary Fig. 2C). We identified 21 differential bacterial genera between the healthy and diarrheal newborn piglets (Supplementary Fig. 2D). Compared with healthy newborn piglets, diarrheal newborn piglets were characterized by five enriched genera (*Vagococcus*, *Sutterella*, *Megasphaera*, *Fusobacterium*, and *Allisonella*) and by 16 depleted genera (*Streptococcus*, *Slackia*, *Ruthenibacterium*, *Peptostreptococcus*, *Lactobacillus*, *Holdemanella*, *Gemella*, *Faecalicoccus*, *Erysipelatoclostridium*, *Eisenbergiella*, *Collinsella*, *Clostridium*, *Christensenella*, *Campylobacter*, *Anaerotruncus*, and *Anaeromassilibacillus*).

**Fig. 2:**
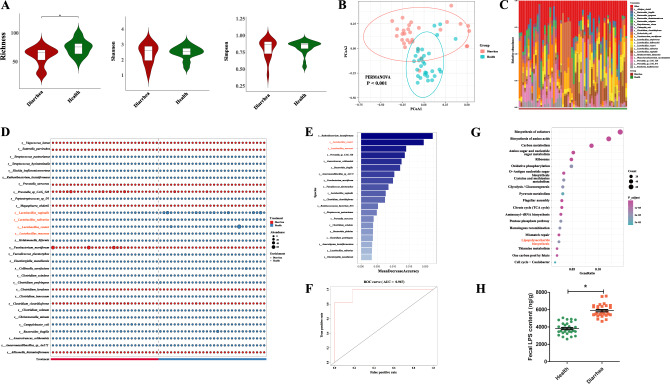
Gut microbiome characteristics at the species level in neonatal diarrheal piglets vs healthy piglets. **A** Comparison of alpha diversity (Richness, Shannon, and Simpson) of gut microbiomes at the species level between healthy and diarrheal newborn piglets. **B** PCoA analysis of gut microbiomes between healthy and diarrheal newborn piglets at the species level; data were analyzed using PERMANOVA. **C** Histogram of the species composition of the top 20 bacterial species. **D** Differential bacteria species between the two groups. **E** Key species obtained from random forest analyses. **F** ROC analysis was performed for the random forest model. **G** The top 20 KEGG pathways enriched by differential KOs. **H** Fecal LPS content. *n* = 30. Data were expressed as the means ± SEM (**H**) and one-way ANOVA was performed, followed by LSD’s test (**H**). *P < 0.05, diarrhea group vs healthy group.

At the species level, the Richness index was significantly lower (*P* = 0.001) in the diarrheal piglets than in the healthy piglets (Fig. 2A). We found significant differences in microbial structure at this level between the two groups by PCoA analysis (*P* = 0.001; Fig. 2B). We observed that the relative abundance of *E. coli* was the highest in diarrheal piglets, and *L. vaginalis* was the highest in healthy piglets (Fig. 2C). In addition, we found that the relative abundances of a total of 31 species were significantly different between the two groups (Fig. 2D). Random forest analyses showed that several key species, including *Ruthenibacterium lactatiformans*, *Limosilactobacillus reuteri* (*L. reuteri*), and *L. mucosae*, may play important roles in mediating the onset of diarrhea (Fig. 2E). The accuracy of the random forest classification model established using these 20 bacteria as variables was tested by receiver operating characteristic curve (ROC) analyses, and the AUC value was found to reach 0.967, indicating the high accuracy of our model (Fig. 2F).

In total, 410 Kyoto Encyclopedia of Genes and Genomes (KEGG) orthologs (KOs) that differed significantly between the two groups were identified (Supplementary Table 1). A KEGG function analysis further revealed that the enrichment of the following pathways differed significantly between the two groups: biosynthesis of cofactors, biosynthesis of amino acids, carbon metabolism, amino sugar and nucleotide sugar metabolism, ribosome, oxidative phosphorylation, O-antigen nucleotide sugar biosynthesis, cysteine and methionine metabolism, glycolysis/gluconeogenesis, pyruvate metabolism, flagellar assembly, citrate cycle, aminoacyl-tRNA biosynthesis, pentose phosphate pathway, homologous recombination, mismatch repair, lipopolysaccharide biosynthesis, thiamine metabolism, one carbon pool by folate, and the Caulobacter cell cycle (Fig. 2G). We further observed that all KOs enriched in the lipopolysaccharide (LPS) biosynthesis pathway were elevated in diarrheal newborn piglets (Supplementary Table 1). Accordingly, fecal LPS content was significantly higher in the diarrheal piglets compared to the healthy piglets (*P* < 0.05; Fig. 2H).

### The transfer of fecal microbiota from diarrheal piglets causes growth retardation, systemic inflammation, and gut inflammatory damage in GF mice

**Fig. 3:**
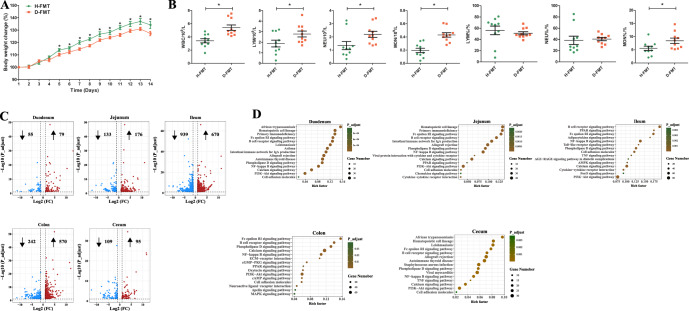
Evaluation of growth, systemic inflammation, and gut transcriptome characteristics in H-FMT GF mice vs D-FMT GF mice. **A** Body weights of mice during the experimental period. **B** Levels of WBC, LYM, NEU, MON, LYM%, NEU%, and MON% in the blood of experimental mice. **C** Volcano plot of differential genes in five intestinal segments. Blue denotes genes that are significantly downregulated in the D-FMT group, and red denotes genes that are significantly upregulated in the D-FMT group. **D** KEGG signaling pathways enriched in five intestinal segment differential genes. The sizes of the circles represent the number of genes enriched in the pathway, and the color is related to *P_adjust*. *n* = 10 for **A**, **B**; *n* = 4 for **C**, **D**. Data were expressed as the means ± SEM (**A**, **B**) and one-way ANOVA was performed, followed by LSD’s test (**A**, **B**). *P < 0.05: H-FMT group vs D-FMT group.

To evaluate the effect of gut microbiota of diarrheal piglets on host growth and inflammatory status, we performed an FMT experiment (Fig. 1B). We found that the rate of increase of body weight of the H-FMT group was significantly higher than that of the D-FMT group beginning on day 5, and this difference in rates persisted until the end of the experiment (Fig. 3A). We measured routine biochemical parameters in the blood (Fig. 3B); total white blood cell count (WBC) (*P* = 0.001), lymphocytes (LYM) (*P* = 0.049), neutrophil granulocytes (NEU) (*P* = 0.041), monocytes (MON) (*P* = 0.000), and MON% (*P* = 0.039) were significantly higher in the D-FMT group.

We sequenced and analyzed the transcriptomes of host cells from the duodenum, jejunum, ileum, cecum, and colon of both groups of mice. The gene profiles in these five intestinal segments exhibited distinct patterns after FMT (Fig. 3C). We found that there were 134 differential genes in the duodenum, of which 79 genes were significantly upregulated and 55 genes were significantly downregulated in the D-FMT group. There were 309 differential genes in the jejunum, with 176 genes significantly upregulated and 133 genes significantly downregulated in the D-FMT group. There were 1609 differential genes in the ileum, with 670 genes significantly upregulated and 939 genes significantly downregulated in the D-FMT group. There were 204 differential genes in the cecum, with 95 genes significantly upregulated and 109 genes significantly downregulated in the D-FMT group. There were 812 differential genes in the colon, with 570 genes significantly upregulated and 242 genes significantly downregulated in the D-FMT group.

We annotated the pathways in which these differential genes are involved (Fig. 3D) and found that several signaling pathways were significantly enriched in all five intestinal segments, including the NF-κB signaling pathway, the phospholipase D signaling pathway, cell adhesion molecules, B cell receptor signaling pathways, the Fc epsilon RI signaling pathway, the phosphoinositide 3-kinase/Akt signaling pathway, and calcium signaling pathways. Moreover, we showed that the major differential genes involved in the above signaling pathways were observed in all intestinal segments (Supplementary Fig. 3).

**Fig. 4:**
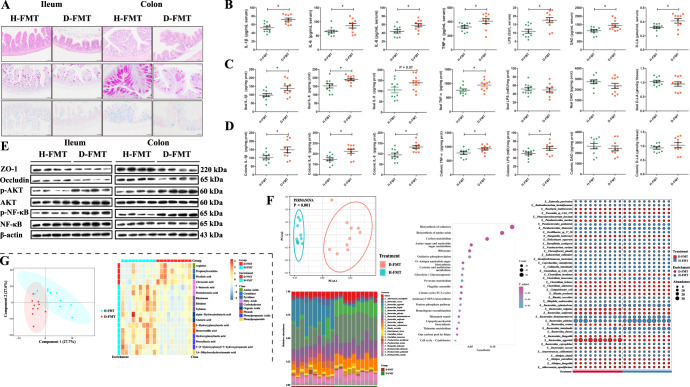
Characteristics of the gut barrier, inflammatory response, and microenvironment in H-FMT GF mice vs D-FMT GF mice. **A** Ileum and colon tissue sections from H-FMT and D-FMT mice: the first row of the image show H&E staining, the second row shows PAS staining, and the third row shows AB staining (scale bar, 50 μm). **B** The levels of IL-1β, IL-6, IL-8, TNF-α, LPS, DAO, and D-LA in the serum. **C** The levels of IL-1β, IL-6, IL-8, TNF-α, LPS, DAO, and D-LA in ileum tissue. **D** The levels of IL-1β, IL-6, IL-8, TNF-α, LPS, DAO, and D-LA in colon tissue. **E** Levels of ZO-1, occludin, AKT, and NF-κB proteins and p-AKT and p-NF-κB in ileum and colon. **F** PCoA analysis at the species levels: data were analyzed using PERMANOVA; Species composition histogram of the top 20 species; The top 20 KEGG pathways enriched in differential KOs; Microbial bubble plots of the differences between H-FMT and D-FMT groups at the species level. **G** PLS-DA analysis of the metabolome; The heatmap of differential metabolites. The left side of the picture is the enriched group of metabolites, and the right side is the name of the metabolite and its corresponding class. *n* = 10 for **A**–**D**, **F**, **G**; *n* = 4 for **E**. Data were expressed as the means ± SEM (**B**–**D**) and one-way ANOVA was performed, followed by LSD’s test (**B**–**D**). *P < 0.05: H-FMT group vs D-FMT group.

We found that the length of the ileum villus in mice of the D-FMT group was significantly shortened (*P* = 0.000), and the villus to crypt length ratio in the D-FMT group was significantly lower (*P* = 0.000) than that of H-FMT group (Fig. 4A and Supplementary Fig. 4A). The colonic histopathological score was significantly higher in the D-FMT group (*P* = 0.013; Fig. 4A and Supplementary Fig. 4A), and the number of neutral mucins and acid mucins was significantly lower in the D-FMT group than in the H-FMT group in both ileum (*P* = 0.013/0.009) and colon (*P* = 0.043/0.029) (Fig. 4A and Supplementary Fig. 4A).

We also measured the indicators related to inflammation and intestinal permeability in the serum, ileum, and colon. The serum levels of interleukin (IL)-1β (*P* = 0.001), IL-6 (*P* = 0.041), IL-8 (*P* = 0.017), tumor necrosis factor (TNF)-α (*P* = 0.045), LPS (*P* = 0.004), diamine peroxidase (DAO) (*P* = 0.019), and d-lactate (d-LA) (*P* = 0.021) concentrations were significantly higher in the D-FMT group than in the H-FMT group (Fig. 4B). The levels of IL-1β (*P* = 0.029), IL-6 (*P* = 0.013), and TNF-α (*P* = 0.035) were significantly higher in the ilea of mice in the D-FMT group than in the H-FMT group, but there was no significant difference in levels of LPS, IL-8, DAO, or D-LA in the ilea of GF mice between the two groups (Fig. 4C). The levels of IL-1β (*P* = 0.041), IL-6 (*P* = 0.005), IL-8 (*P* = 0.004), TNF-α (*P* = 0.039) and LPS (*P* = 0.037) in the colon were significantly higher in the D-FMT group than in the H-FMT group, while DAO and d-LA were not significantly different (Fig. 4D) between the two groups. As shown in Fig. 4E, the levels of expression of ZO-1 and occludin proteins in the D-FMT group were significantly decreased in both the ileum and colon. Moreover, the protein abundance of total AKT and NF-κB were similar between D-FMT and H-FMT groups, but the levels of p-AKT and p-NF-κB in D-FMT were significantly higher in the D-FMT group than those in H-FMT group.

### The transfer of fecal microbiota from diarrheal piglets to GF mice causes disorders of gut microbiota and metabolites

To investigate the composition and function of the gut microbiome in GF mice after FMT, we performed metagenomic sequencing analyses on fecal samples. At the phylum level, there was a significant difference in the Richness index (*P* = 0.003; Supplementary Fig. 5A). No difference in beta diversity at the phylum level was observed between the two groups (Supplementary Fig. 5B). We analyzed the phylum composition of the two groups (Supplementary Fig. 5C) and found that the microbiota from both groups were mainly composed of Bacteroidetes and Firmicutes. Further analysis of differences in bacteria showed that a significantly lower relative abundance of Actinobacteria was observed in the D-FMT group (Supplementary Fig. 5D).

At the genus level, the D-FMT group had a significantly lower Shannon index (*P* = 0.015) and Simpson (*P* = 0.003) index than did the H-FMT group (Supplementary Fig. 6A). We also found significant differences in bacterial composition at the genus level between the two groups (*P* = 0.001; Supplementary Fig. 6B). The microbes in both groups were mainly composed of *Bacteroides* and *Parabacteroides* (Supplementary Figure 6C), and a differential analysis identified 24 genera, including *Bacteroides*, *Parabacteroides*, *Erysipelatoclostridium*, and several others (Supplementary Fig. 6D).

At the species level, there were significant differences in microbiota structure between the two groups as determined by PCoA analyses (*P* = 0.001; Fig. 4F). Microbial composition results showed that *Bacteroides eggerthii*, *B. plebeius*, and *B. thetaiotaomicron* were the dominant species in the D-FMT group, while *B. plebeius*, *B. thetaiotaomicron*, and *B. intestinalis* were the dominant species in the H-FMT group (Fig. 4F). A total of 44 bacterial species that differed significantly between the two groups were identified (Fig. 4F). There were also 873 KOs that differed significantly between the two groups (Supplementary Table 2). Thirteen KOs were enriched in the LPS biosynthesis pathway, including 10 KOs that were upregulated in the D-FMT group (Fig. 4F and Supplementary Table 2).

We evaluated the similarity of the gut microbiomes in GF mice and piglets (Supplementary Fig. 7A). We found that the gut microbes in the two groups were completely matched at the phylum level. At the genus level, the homology rate reached 82.71% in the healthy group and 80.67% in the diarrhea group. At the species level, the homology rate reached 83.61% in the healthy group and 63.67% in the diarrhea group.

Fecal metabolomics analyses were also performed on both groups of recipient mice. The overall distribution of metabolites and the composition of metabolites in the two groups are shown in Supplementary Fig. 7B, C. There were significant differences in the metabolites between these two groups (Fig. 4G). Further analysis of the data revealed that 17 metabolites differed between the two groups, with arginine, propionylcarnitine, picolinic acid, and citraconic acid having higher levels in the D-FMT group, and 2-butenoic acid, pentadecanoic acid, rhamnose, ribulose, xylulose, alpha-hydroxyisobutyric acid, glutaric acid, 3-hydroxyphenylacetic acid, homovanillic acid, hydroxyphenyllactic acid, phenyllactic acid, 3-(3-hydroxyphenyl)-3-hydroxypropanoic acid, and 3,4-dihydroxyhydrocinnamic acid having significantly higher levels in the H-FMT group (Fig. 4G). KEGG analyses revealed arginine and proline metabolism, pentose and glucuronate interconversions, and aminoacyl-tRNA biosynthesis to be the pathways in which the metabolites were enriched (Supplementary Fig. 7D).

### *L. mucosae* alleviates diarrheal disease symptoms by inhibiting inflammatory responses and regulating the gut microbiota in diarrheal recipient GF mice

**Fig. 5:**
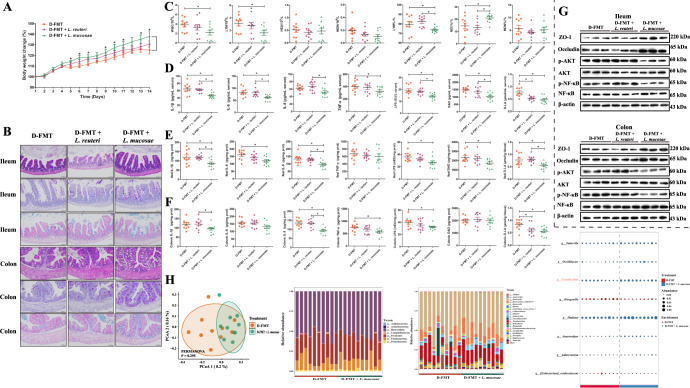
The protective effect of *L. mucosae* and *L. reuteri* on intestinal damage caused by fecal microbiota in diarrheal piglets. **A** Body weights of mice were determined during the experimental period. **B** Ileum and colon tissue sections of experimental mice: the first row of the image show H&E staining, the second row shows PAS staining, and the third row shows AB staining (scale bar, 50 μm). **C** Levels of WBC, LYM, NEU, MON, LYM%, NEU%, and MON% in the blood of experimental mice. **D** The levels of IL-1β, IL-6, IL-8, TNF-α, LPS, DAO, and D-LA in the serum. **E** The levels of IL-1β, IL-6, IL-8, TNF-α, LPS, DAO, and D-LA in ileum tissue. **F** The levels of IL-1β, IL-6, IL-8, TNF-α, LPS, DAO, and D-LA in colon tissue. **G** Levels of ZO-1, occludin, AKT, and NF-κB proteins and p-AKT and p-NF-κB in ileum and colon. **H** Principal coordinates analysis at the ASV level; Relative abundance of the bacterial composition of the fecal microbiota of GF mice at the phylum and genus level; Bubble diagram for fecal microbial differential analysis at the genus level. *n* = 10 for **A**–**F** and **H**; *n* = 3 for **G**. Data were expressed as the means ± SEM (**A** and **C**–**F**) and one-way ANOVA was performed, followed by LSD’s test (**A** and **C**–**F**). *P < 0.05.

We isolated *L. reuteri* and *L. mucosae* from healthy newborn piglet fecal samples and investigated the effect of these two specific *Lactobacilli* on the diarrheal disease symptoms in diarrheal recipient GF mice (Fig. 1C). Upon measuring the body weights of the GF mice over time, we found that from day 5, the GF mice fed with *L. mucosae* exhibited significant improvement to the growth retardation caused by administration of the diarrhea fecal microbiota suspension, while the *L. reuteri* intervention group was not significantly different from the D-FMT group (Fig. 5A).

By histological observation of the colon and ileum of mice in the three groups (Fig. 5B), we found that villus length (*P* = 0.004), neutral mucins (*P* = 0.028), and acid mucins (*P* = 0.003) of the ileum were significantly elevated in the *L. mucosae* intervention group compared to the D-FMT group (Supplementary Fig. 4B). Moreover, the histopathological score of the colon was significantly lower (*P* = 0.002; Supplementary Fig. 4B). The neutral mucins (*P* = 0.010) and acid mucins (*P* = 0.003) of the colon were significantly higher in the *L. mucosae* intervention group compared to the D-FMT group (Supplementary Fig. 4B). However, the differences in these indicators did not reach the level of statistical significance when comparing the ileum and colon in the *L. reuteri* intervention group to those of the D-FMT group (Supplementary Fig. 4B).

Blood biochemical indexes results showed that the *L. mucosae* intervention group had significantly lower WBC (*P* = 0.033), LYM (*P* = 0.022), and LYM% (*P* = 0.046) compared with the D-FMT group, while the NEU% (*P* = 0.019) was markedly higher in the *L. mucosae* intervention group (Fig. 5C). In addition, serum levels of IL-1β (*P* = 0.002), IL-6 (*P* = 0.007), TNF-α (*P* = 0.050), LPS (*P* = 0.003), DAO (*P* = 0.002), and d-LA (*P* = 0.000) in the *L. mucosae* intervention group were significantly lower than those in the D-FMT group (Fig. 5D). We also found that the levels of IL-1β (*P* = 0.012), IL-6 (*P* = 0.005), IL-8 (*P* = 0.004), LPS (*P* = 0.009), DAO (*P* = 0.029), and d-LA (*P* = 0.011) in the ileum of the *L. mucosae* intervention group were significantly lower than those in the D-FMT group (Fig. 5E).

For the colon, the contents of IL-8 (*P* = 0.000), TNF-α (*P* = 0.017), LPS (*P* = 0.036), and D-LA (*P* = 0.007) were significantly lower in the *L. mucosae* intervention group than in the D-FMT group (Fig. 5F). We also found that the levels of expression of ZO-1 and occludin proteins in the ileum and colon were significantly higher in the *L. mucosae* intervention group (Fig. 5G). The levels of p-AKT and p-NF-κB were significantly lower in the *L. mucosae* intervention group (Fig. 5G), and these values were not significantly different between the *L. reuteri* intervention group and the D-FMT group (Fig. 5G).

We also analyzed changes in the structure and composition of the intestinal microbiota after *L. mucosae* intervention (Fig. 5H). PCoA showed that bacterial signatures between the D-FMT and the *L. mucosae* intervention groups were not significantly distinct at the amplicon sequence variant (ASV) level. At the phylum level, Bacteroidota, Firmicutes, and Fusobacteriota were the predominant bacteria in the feces of mice of the D-FMT and *L. mucosae* intervention groups. At the genus level, *Bacteroides*, *Fusobacterium*, and *Bacteroidetes_vadinHA17* were the predominant bacteria in the feces of both D-FMT mice and those receiving the *L. mucosae* intervention. Moreover, we identified a total of eight discriminative bacterial genus between the D-FMT group and the *L. mucosae* intervention group. Compared with the D-FMT group, the *L. mucosae* intervention group was characterized by six enriched species and two depleted species.

### *L. mucosae* alleviates diarrheal disease symptoms by inhibiting inflammatory responses and regulating the gut microbiota in ETEC K88 challenge mice

**Fig. 6:**
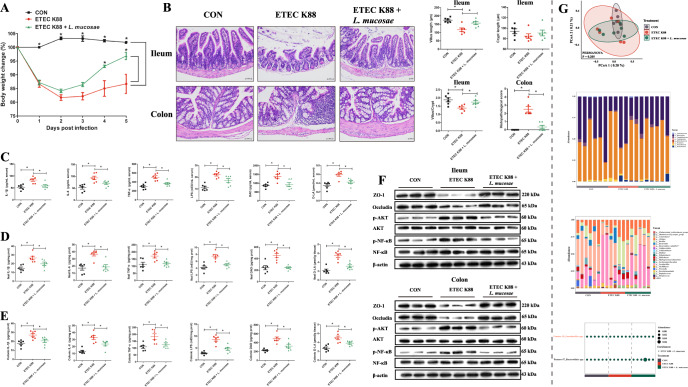
The protective effect of *L. mucosae* on intestinal damage caused by ETEC K88. **A** Body weights of mice were determined during the experimental period. **B** H&E staining in the ileum and colon tissue sections of experimental mice; The villus length, crypt length, ratio of the villus to crypt length of the ileum, and histopathological score of the colon (scale bar, 50 μm). **C** The levels of IL-1β, IL-6, IL-8, TNF-α, LPS, DAO, and D-LA in the serum. **D** The levels of IL-1β, IL-6, IL-8, TNF-α, LPS, DAO, and D-LA in ileum tissue. **E** The levels of IL-1β, IL-6, IL-8, TNF-α, LPS, DAO, and D-LA in colon tissue. **F** Levels of ZO-1, occludin, AKT, and NF-κB proteins and p-AKT and p-NF-κB in ileum and colon. **G** Principal coordinates analysis at the ASV level; Relative abundance of the bacterial composition of the fecal microbiota of mice at the phylum and genus level; Bubble diagram for fecal microbial differential analysis at the ASV level. *n* = 6 for **A**–**E** and **G**; *n* = 3 for **F**. Data were expressed as the means ± SEM (**A**–**E**) and one-way ANOVA was performed, followed by LSD’s test (**A**–**E**). * *P* < 0.05.

We also explored the mitigation effect of *L. mucosae* on the diarrheal disease symptoms induced with ETEC K88 (Fig. 1D). Upon measuring the body weights of the mice during the experiment, we found that the mice fed with *L. mucosae* exhibited a significant improvement in the growth retardation caused by ETEC K88 infection (Fig. 6A). By histological observation of the colon and ileum of the three groups of mice (Fig. 6B), the villus length (*P* = 0.005) and villus to crypt length ratio (*P* = 0.024) of the ileum were found to be significantly elevated in the ETEC K88 + *L. mucosae* group compared to the ETEC K88 group (Fig. 6B). Moreover, the histopathological score of the colon was significantly lower (*P* = 0.000; Fig. 6B) in the intervention group. The serum, ileum, and colon levels of IL-1β (*P* = 0.004/0.025/0.021), IL-6 (*P* = 0.022/0.001/0.015), TNF-α (*P* = 0.002/0.010/0.016), LPS (*P* = 0.018/0.001/0.000), DAO (*P* = 0.003/0.000/0.004), and D-LA (*P* = 0.003/0.000/0.019) in the ETEC K88 + *L. mucosae* group were significantly lower than those of the ETEC K88 group (Fig. 6C–E). The levels of expression of ZO-1 and occludin proteins in the ileum and colon were significantly higher in the ETEC K88 + *L. mucosae* group than in the ETEC K88 group, whereas the levels of p-AKT and p-NF-κB were significantly lower (Fig. 6F).

We also analyzed changes in the structure and composition of the intestinal microbiota after *L. mucosae* intervention (Fig. 6G). PCoA showed that bacterial signatures between the three groups were not significantly distinct at the ASV level. At the phylum level, Firmicutes, Bacteroidota, and Campilobacterota were the predominant bacteria in the feces of ETEC K88 mice, whereas Firmicutes, Bacteroidota, and Proteobacteria were the predominant bacteria in the feces of ETEC K88 + *L. mucosae* mice. At the genus level, *Lactobacillus*, *Bacteroidetes_vadinHA17*, and *Alistipes* were the predominant bacteria in the feces of both ETEC K88 and ETEC K88 + *L. mucosae* mice. We identified a total of two discriminative microbes between the ETEC K88 and ETEC K88 + *L. mucosae* groups at the ASV level; compared with the ETEC K88 group, the ETEC K88 + *L. mucosae* group was characterized by two enriched bacteria. The characteristics of the gut microbiota in the control and ETEC K88 + *L. mucosae* groups at the end of the *L. mucosae* intervention but prior to the ETEC K88 challenge are shown in Supplementary Fig. 8.

### *L. mucosae*-derived EVs alleviate diarrheal disease symptoms by mediating macrophage phenotypes in ETEC K88 challenge mice

**Fig. 7:**
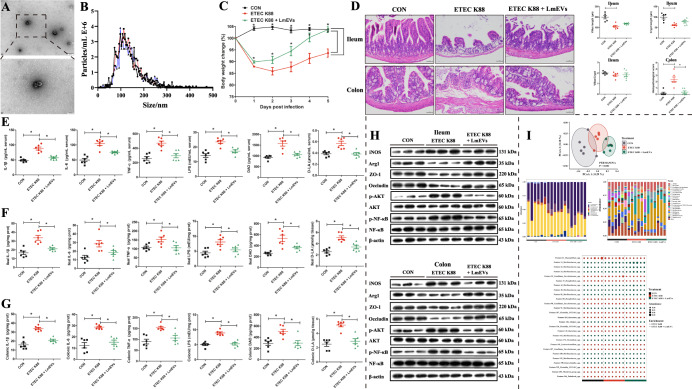
The protective effect of EVs of *L. mucosae* on intestinal damage caused by ETEC K88. **A** TEM of isolated EVs (scale bar, 200 or 100 nm). **B** Size distribution of EVs analyzed by NTA. **C** Body weights of mice during the experimental period. **D** H&E staining of ileum and colon tissue sections of experimental mice; Measurements of villus length, crypt length, and villus to crypt length ratios of the ileum and the histopathological scores of the colon (scale bar, 50 μm). Levels of IL-1β, IL-6, TNF-α, LPS, DAO, and D-LA in **E** the serum, **F** the ileum tissue, and **G** the colon tissue. **H** Expression of iNOS, Arg1, ZO-1, occludin, AKT, and NF-κB proteins and levels of p-AKT and p-NF-κB in ileum and colon. **I** Principal coordinates analysis at the ASV level; Relative abundance of the bacterial composition of the fecal microbiota of mice at the phylum and genus level; Bubble diagram for fecal microbial differential analysis at ASV level. *n* = 3 for **A** and **B**; *n* = 6 for **C**, **E**–**G**, and **I**; *n* = 5/6 for **D**; *n* = 3 for **H**. Data were expressed as the means ± SEM (**C**–**G**) and one-way ANOVA was performed, followed by LSD’s test (**C**–**G**). *P < 0.05.

We further explored the mitigation effect of *L. mucosae*-derived EVs on the diarrheal disease symptoms caused by ETEC K88 (Fig. 1E). Transmission electron microscopy (TEM) was used to confirm the presence of EVs in the *L. mucosae* cultures (Fig. 7A). EVs of *L. mucosae* (LmEVs) were also quantified by nanoparticle tracking analysis (NTA) (Fig. 7B). Biochemical analysis revealed that the LmEVs contained DNA, RNA, and proteins, and the protein content was noticeably higher than that of DNA or RNA (Supplementary Fig. 9). After co-incubation with MODE-K or IPEC-J2 cells for 6 h, DiI-labeled LmEVs (red signal) were found in the cytoplasm of these cells, suggesting that LmEVs were internalized by intestinal epithelial cells (Supplementary Fig. 10). By measuring the body weights of the mice during the experiment, we determined that mice fed with *L. mucosae*-derived EVs exhibited a significant improvement to the growth retardation caused by ETEC K88 infection (Fig. 7C). Upon histological observation of the colon and ileum of the three groups of mice (Fig. 7D), the histopathological score of the colon was determined to be significantly lower in the intervention group (*P* = 0.000; Fig. 7D). In addition, the serum, ileum, and colon levels of IL-1β (*P* = 0.000/0.001/0.000), IL-6 (*P* = 0.001/0.025/0.000), TNF-α (*P* = 0.000/0.011/0.004), LPS (*P* = 0.001/0.014/0.000), DAO (*P* = 0.000/0.006/0.003), and d-LA (*P* = 0.000/0.001/0.000) in the ETEC K88 + LmEVs group were significantly lower than those of the ETEC K88 group (Fig. 7E–G). We also found that the levels of expression of Arg1, ZO-1, and occludin proteins in the ileum and colon were significantly higher in the ETEC K88 + LmEVs group than in the ETEC K88 group, whereas the expression of iNOS and the levels of p-AKT and p-NF-κB proteins were significantly lower (Fig. 7H).

We also analyzed changes in the structure and composition of the intestinal microbiota after LmEV intervention (Fig. 7I). PCoA showed that bacterial signatures among the three groups were significantly distinct at the ASV level. At the phylum level, Bacteroidota, Firmicutes, and Campilobacterota were the predominant bacteria in the feces of mice from the ETEC K88 and ETEC K88 + LmEVs groups. At the genus level, *Muribaculaceae*, *Alistipes*, and *Lactobacillus* were the predominant bacteria in the feces of the ETEC K88 group, whereas *Muribaculaceae*, *Lactobacillus*, and *Alistipes* were the predominant bacteria in the feces of ETEC K88 + LmEVs mice. Moreover, we identified a total of 27 discriminative bacteria between the ETEC K88 and ETEC K88 + LmEVs groups at the ASV level; compared with the ETEC K88 group, the ETEC K88 + LmEVs group was characterized by 13 enriched bacteria and by 14 depleted bacteria.

### Macrophages are necessary for *L. mucosae*-derived EVs to alleviate diarrheal disease symptoms in ETEC K88 challenge mice

**Fig. 8:**
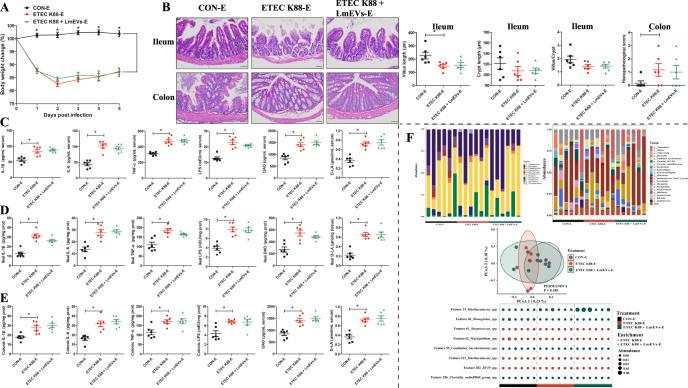
Effect of macrophage clearance on *L. mucosae*-derived EVs in the alleviation of ETEC K88-induced intestinal injury. **A** Body weights of mice over the experimental period. **B** H&E staining of ileum and colon tissue sections of experimental mice; Measurements of villus length, crypt length, and the villus to crypt length ratio of the ileum, and histopathological scores of the colon (scale bar, 50 μm). The levels of IL-1β, IL-6, TNF-α, LPS, DAO, and D-LA in the **C** serum, **D** ileum tissues, and **E** colon tissues. **F** Principal coordinates analysis at the ASV level; Relative abundance of the bacterial composition of the fecal microbiota of mice at the phylum and genus level; Bubble diagram for fecal microbial differential analysis at the ASV level. *n* = 6 for **A**–**F**. Data were expressed as the means ± SEM (**A**–**E**) and one-way ANOVA was performed, followed by LSD’s test (**A**–**E**). *P < 0.05.

To elucidate the roles of macrophages in the mitigation of ETEC K88 challenge-induced diarrheal disease symptoms by LmEVs, we performed a macrophage elimination experiment (Fig. 1F). No difference was found in body weight changes between macrophage-eliminated mice challenged with ETEC K88 (the ETEC K88-E group) and ETEC K88-E mice fed LmEVs (the ETEC K88 + LmEVs-E group) (Fig. 8A). There were no significant differences in jejunal villus length, crypt length, villus to crypt length ratio or colonic histopathology scores between ETEC K88 + LmEVs-E and ETEC K88-E groups (Fig. 8B). There were also no obvious differences in the serum, ileum, and colon levels of IL-1β, IL-6, TNF-α, LPS, DAO, and d-LA between ETEC K88 + LmEVs-E and ETEC K88-E groups (Fig. 8C–E).

The changes in the structure and composition of the intestinal microbiota after LmEV intervention are shown in Fig. 8F. PCoA showed that bacterial signatures between the three groups were not significantly distinct at the ASV level. At the phylum level, Firmicutes, Bacteroidota, and Proteobacteria were the predominant bacteria in the feces of ETEC K88-E mice and ETEC K88 + LmEV-E mice. At the genus level, *Lactobacillus*, *Muribaculaceae*, and *Acinetobacter* were the most dominant genus in the feces of ETEC K88-E mice, whereas *Lactobacillus*, *Muribaculaceae*, and *Alistipes* were the most dominant genus in the feces of ETEC K88 + LmEV-E mice. Moreover, we identified a total of eight discriminative microbes between the ETEC K88-E and ETEC K88 + LmEVs-E groups at the ASV level. Compared with the ETEC K88-E group, the ETEC K88 + LmEVs-E group was characterized by four enriched bacteria and by four depleted bacteria.

## Discussion

Diarrheal disease is a global health problem for humans and livestock^38^. Diarrhea is estimated to occur in millions of patients, and diarrheal diseases cause billions of dollars in medical expenditures worldwide each year^39^. Diarrhea is hypothesized to be caused in part by dysbiosis of the gut microbiota, which acts as a link between host disease phenotype and the physiological function of tissues and organs^24^. Therefore, it is essential to elucidate the mechanisms underlying diarrheal disorders, especially from the perspective of the intestinal microbiota, in order to develop new prevention and treatment strategies. Here, we first used healthy and diarrheal neonatal piglets as models to systematically reveal the characteristics of neonatal gut microbiomes under these different conditions, and we identified specific potential probiotic strains that distinguish healthy and diarrheal individuals. Secondly, we transplanted fecal microbiota from healthy and diarrheal neonatal piglets to GF mice to elucidate the molecular mechanisms by which the disordered intestinal microbiota of diarrheal neonates led to intestinal damage and growth retardation. In addition, we performed intervention trials using specific probiotic components isolated from healthy individuals and demonstrated superior alleviative efficacy against diarrheal disease symptoms. Finally, we demonstrated that probiotic-derived EVs modulated macrophage phenotypes to counteract diarrheal disease symptoms.

The use of newborn piglets as a translational model allowed elucidation of the intestinal microbiota characteristics of healthy and diarrheal individuals. The results of our metagenomic analysis showed that neonatal piglets with diarrhea are indeed suffering from intestinal microbiota disorder, which is characterized by a distinct intestinal microbiota structure from that of healthy individuals. *Lactobacilli spp*. are currently recognized as probiotics that play an important role in maintaining intestinal microbiota homeostasis^40^. Our data revealed that *Lactobacillus spp*. are in the highest relative abundance in the guts of both healthy and diarrheal neonates, but that *Lactobacillus spp*. and some specific *Lactobacillus* species are significantly deficient in the guts of diarrheal neonates. We further identified *L. reuteri* and *L. mucosae* as key microorganisms that distinguished healthy and diarrheal neonates using random forest models. *E. coli* is the major enteric pathogen responsible for diarrheal disease, and children under 5 years of age are susceptible to *E. coli* infection^41^. In our study, *Escherichia* was the second predominant genus in the feces of diarrheal newborn piglets. Furthermore, although no statistical differences existed, the relative abundance of *E. coli* in the feces of diarrheal piglets was twice of that of healthy piglets. LPS is a classical virulence factor produced by *E. coli* that causes diarrhea^42,43^, and our KEGG functional analysis revealed that LPS biosynthesis was significantly higher in diarrheal newborn piglets than in healthy newborn piglets. The Fusobacteria are a small group of Gram-negative bacteria, of which *Fusobacterium* are commonly found in the digestive tract of humans and animals. The main representative species of *Fusobacterium* is *Fusobacterium nucleatum*, which has been shown to be strongly associated with intestinal inflammation and colorectal cancer^44,45^. In our study, the level of Fusobacteria was significantly higher in the diarrheal newborn piglet, suggesting that *Fusobacterium* may be an intermediate mediator of intestinal inflammation in piglets suffering from *E. coli*-induced diarrhea. Our research favors elucidating the potential mechanisms by which *Lactobacilli* alleviate diarrheal disease rather than the pathogenesis of diarrhea, so have left the study of pathogenic bacteria to future work. The present results suggest that *E. coli* challenge and LPS overproduction are important risk factors for neonatal diarrhea, while the absence of *Lactobacillus* severely weakens the resistance to the pathogenic invasion of the neonatal intestinal microbiota.

In addition to its potential as a therapy for complex diseases, FMT has likewise helped to reveal the intrinsic link between gut microbes and host disease phenotypes and physiological functions of tissues and organs^46–48^. In our study, transplantation of fecal microbiota from diarrheal piglets to GF mice induced significant growth retardation accompanied by severe systemic inflammatory responses. Consistent with results in newborn piglets, there were significant differences in the intestinal microbiota structure between GF mice that were recipients of healthy and diarrheal piglet microbial samples. Although the vast majority of the intestinal microbiota of recipient mice was derived from donor pigs, the species composition of the mice intestinal microbiota was radically altered, a phenomenon common to many FMT studies^49,50^. *Bacteroides* play an important role in intestinal microbiota-host interactions, and previous studies have shown that *Bacteroides* can regulate the production of pro-inflammatory cytokines^51,52^. Our data showed that *Bacteroides* was dominant in the intestinal microbiota of both groups of mice, and the relative abundance of *Bacteroides* in the feces of diarrheal recipient mice was significantly higher than that of healthy recipient mice. Notably, some intestinal microbiota-related functional pathways were transferred from the donor pigs to the recipient mice, such as LPS biosynthesis. Consistently, our results showed that the serum concentrations of LPS were significantly higher in the diarrheal recipient mice than in the healthy recipient mice. Fecal metabolites are considered to be a functional readout of the gut microbiota^53^, and there were also significant differences between the fecal metabolites of the two groups of recipient mice in our study. The results of the identification of differential metabolites showed that some metabolites with anti-inflammatory potential (3,4-dihydroxyhydrocinnamic acid^54^, homovanillic acid^55^, and phenyllactic acid^56^) were significantly enriched in healthy recipient mice, whereas a pro-inflammatory metabolite (picolinic acid^57^) was enriched in diarrheal recipient mice. These data imply that the disturbing of intestinal microbiota and their metabolites in diarrheal neonates are responsible for the growth retardation and inflammatory responses secondary to diarrheal disease.

In addition to leading to high infant mortality, diarrheal disease is an important driver of intestinal dysplasia^58,59^. Intestinal dysplasia is mainly manifested as a poorly developed intestinal mucosal immune system and impaired intestinal barrier function, which increases intestinal permeability and bacterial translocation, causing systemic inflammatory responses and ultimately leading to neonatal growth retardation^60^. In this study, the disturbed intestinal microbiota of diarrheal piglets caused profound effects on intestinal development in recipient GF mice, mainly in the form of dramatic changes in the gene expression profiles of all intestinal segment tissues (duodenum, jejunum, ileum, cecum, and colon). KEGG enrichment analysis revealed that differential genes in the five intestinal segments mapped mainly to inflammatory and immune-related signaling pathways, such as NF-κB and PI3K-AKT signaling pathways. NF-κB and AKT are important protein molecules that mediate inflammatory responses and are phosphorylated to activate their biological functions^61,62^. Our results showed that phosphorylation of NF-κB and AKT are both elevated in the intestines of diarrheal recipient mice, with similarly elevated levels of pro-inflammatory cytokines in intestinal tissues. Immunodeficiency and inflammatory responses impair intestinal barrier function, disrupt intestinal morphology and structure, and increase intestinal permeability, thus leading to the infiltration of harmful substances such as LPS from the intestinal lumen into the blood circulation^63,64^. Consistently, transplantation of fecal microbiota from diarrheal piglets severely deteriorated intestinal tight junction proteins and intestinal morphology in recipient mice. These findings demonstrate that the disordered intestinal microbiota of diarrheal neonates induces a local inflammatory response in the intestine through the activation of inflammatory signaling pathways, which in turn leads to defects in intestinal development.

In clinical practice, FMT has been widely used in attempts to cure gastrointestinal disorders, and it has shown some efficacy^65,66^. A previous study found that FMT reduced the severity of disease by increasing the relative abundance of probiotics^67^. As mentioned above, we identified *L. reuteri* and *L. mucosae* as key intestinal microorganisms that distinguish between healthy and diarrheal neonates. To verify whether their replenishment could rescue diarrhea-driven intestinal microbiota-induced growth retardation and intestinal damage, we further conducted probiotic intervention experiments. Previous studies have shown that *L. reuteri* and *L. mucosae* have anti-inflammatory capacity^68,69^. Accordingly, our data showed that *L. mucosae* but not *L. reuteri* effectively abrogated the inflammatory response and intestinal damage by inhibiting LPS synthesis and the phosphorylation of NF-κB and AKT, thus effectively alleviating the growth retardation caused by diarrhea-driven gut microbiota and ETEC K88 infection. Probiotics typically protect host health by directly killing pathogenic bacteria or by remodeling the intestinal microbiota to enhance resistance to colonization by pathogenic bacteria^18,24,70^. In our study, *L. mucosae* intervention significantly increased the fecal abundance of *Lactobacillus spp*. in mice. These data suggest that *L. mucosae* from healthy individuals play an important role in resistance to diarrhea and intestinal injury by regulating intestinal microbiota. However, the mechanisms by which *L. mucosae* regulates host intestinal homeostasis to alleviate diarrheal disease need to be further elucidated.

Microbial regulation of host physiological functions can be mediated by active mediators secreted by the microbiota in addition to direct interactions with the host^71,72^. Among the different bacterial-derived mediators, EVs have been shown to play a key role in intercellular crosstalk and signaling^73^. The communication between probiotics and host cells through EV secretion has attracted the attention of the scientific community. Recent studies have shown that some probiotic-derived EVs play similar roles to their parental bacteria^74,75^. In this study, we successfully isolated and identified *L. mucosae*-derived EVs. In addition, we also demonstrated LmEVs exerted better efficacy than *L. mucosae* in alleviating diarrheal disease symptoms. A recent study has shown that *L reuteri* DSM 17938-derived EVs alone can completely reproduce the effects of their parental bacteria on gut motility^76^. In addition, there is evidence that *L reuteri* DSM 17938 and BG-R46-derived EVs enhance intestinal barrier function upon being taken up by intestinal epithelial cells, thereby reducing leakage caused by ETEC^77^. EVs from *L reuteri* BBC3 can also be taken up by macrophages to modulate the immune response^78^. However, our data revealed that *L reuteri* failed to relieve diarrheal disease symptoms, so we did not perform follow-up experiments with *L reuteri*-derived EVs. The *L reuteri* used in our study was isolated from the feces of healthy newborn piglets, which differs from the model strain used in the above reports, and the variability between strains is likely to be an important reason for the different findings. A previous study has shown that *L reuteri* DSM 17938 or its EVs can decrease the duration of acute diarrhea and hospitalization for acute gastroenteritis, but does not seem to prevent nosocomial diarrhea or antibiotic-associated diarrhea^79^, indicating that even the beneficial effects of the same strain can differ under different pathological conditions.

Macrophages, as the core cells that initiate and regulate the inflammatory response, can produce a variety of cytokines, promote the proliferation of intestinal epithelial cells, regulate the integrity of the epithelial barrier, and play an important role in maintaining intestinal homeostasis^80,81^. Dependent on converging signals from inflammatory stimuli and the cellular environment, the different phases of macrophage activation are widely described as pro-inflammatory M1 and anti-inflammatory M2 polarization^82,83^. Recent studies have shown a regulatory effect of the NF-κB and AKT signaling pathways on macrophage phenotypes^84,85^. Our data showed that EV intervention activated M2 macrophage polarization and inhibited M1-type macrophage polarization, as well as suppressed the expression of inflammatory regulatory proteins (NF-κB and AKT). Notably, EVs lost the ability to alleviate the diarrheal disease symptoms in a macrophage elimination mouse model. These findings indicated that LmEVs combat diarrhea by modulating the inflammatory response in a macrophage-dependent manner.

**Fig. 9:**
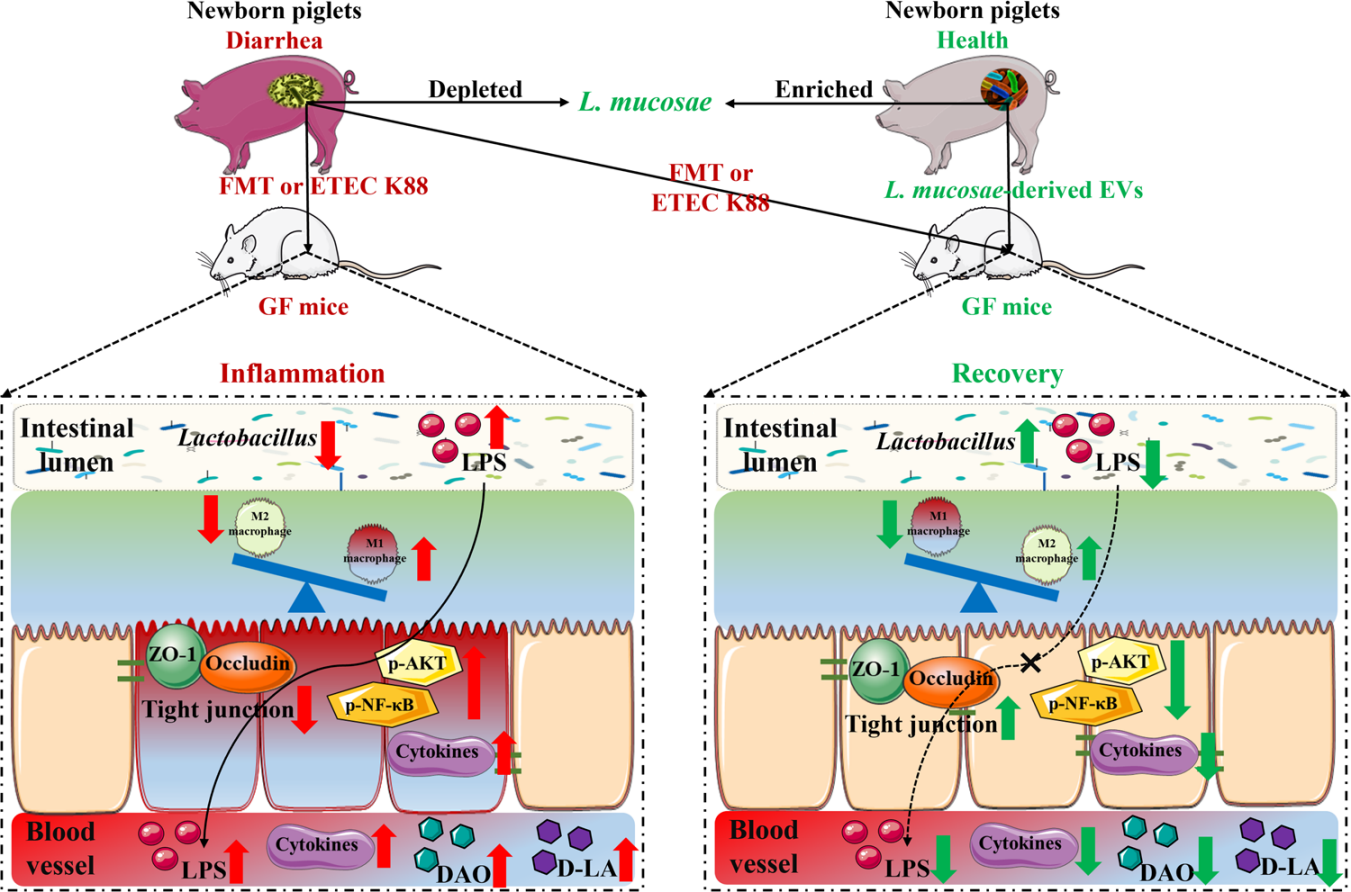
Schematic diagram summarizing the findings in the present study.

In conclusion, intestinal microbiota disorders in diarrheal piglets were characterized by alteration of microbiota structure, absence of *Lactobacillus*, excess *E. coli* proliferation and LPS biosynthesis. *L. mucosae* and *L. reuteri* were potential key microbes that distinguish healthy from diarrheal piglets. Diarrhea-driven gut microbiota successfully conveyed diarrheal disease phenotypes such as growth retardation, inflammatory responses, and intestinal damage to GF mice. Mechanistically, the disordered intestinal microbiota of diarrheal neonates activated pro-inflammatory signaling pathways via LPS, resulting in disrupted intestinal morphological structures, weakened intestinal barrier function, and increased intestinal permeability. Excess LPS translocation from the intestinal lumen to the blood circulation induced a systemic inflammatory response, ultimately leading to growth retardation. *L. mucosae* isolated from the feces of healthy newborn piglets exerted its superior antidiarrheal effect by reducing the inflammatory response and intestinal damage. *L. mucosae*-derived EVs regulated intestinal homeostasis by activating M2 macrophages and inhibiting M1 macrophages, thereby alleviating diarrheal disease symptoms. Overall, by regulating the activity of macrophages through the release of EV, *L. mucosae* ameliorated the sickness caused by gut microbes. The main findings of our study are summarized in Fig. 9. These findings provide a potential strategy for the prevention of diarrhea risk in newborns.

## Methods

### Isolation and culture of the *L. mucosae* and *L. reuteri*

Fecal samples from newborn healthy piglets were randomly selected for *Lactobacillus* isolation. Samples of frozen feces (200 mg) were suspended in sterile phosphate-buffered saline (PBS) buffer and thoroughly disrupted by pipetting in order to create bacterial suspension. This suspension was diluted with a tenfold gradient in sterile PBS, and equal volumes (100 μL) of each dilution were spread on de Man, Rogosa, and Sharpe (MRS) agar medium and incubated anaerobically at 37 °C for 24 h. Single colonies with different morphologies were selected using inoculation loops from plates produced with different dilutions, and the bacteria were purified by scribing on new MRS agar medium and incubated the plates anaerobically at 37 °C for 24 h. After four purification cycles, single colonies were transferred from MRS agar plates into 500 μL of MRS broth, and the liquid was transferred under anaerobic conditions to 15 mL of MRS broth and incubated at 37 °C for 24 h. When the MRS broth was cloudy, DNA was extracted from a portion of the bacterial broth. The universal primers 27 F (5′-AGAGTTTGATCCTGGCTCAG-3′) and 1492 R (5′-GGTTACCTTGTTACGACTT-3′) were used to amplify the 16S rRNA gene of the single strain, and the PCR product was sequenced by the Sanger method. Another portion of the bacterial solution was mixed with an equal volume of 50% sterile glycerol and stored at −80 °C. The 16S rRNA gene sequences were used to scan the NCBI nucleotide sequence database to identify strains of *L. reuteri* and *L. mucosae*.

### Isolation and identification of LmEVs

EVs were isolated from culture supernatants of *L. mucosae* bacteria^78,86^. Briefly, bacterial cultures at the logarithmic stage of growth were centrifuged at 8000 × *g* for 30 min. The supernatant was collected and centrifuged again at 20,000 × *g* for 45 min. This supernatant was then passed through a 0.22 μm filter (Millipore), and the filtrate was centrifuged in an SW 32 Ti rotor (Beckman Coulter, Fullerton, CA, USA) at 120,000 × *g* for 2 h at 4 °C. The supernatant was discarded, and the sediment was resuspended in PBS, and the suspension was filtered and recentrifuged at 120,000 × *g* for 2 h at 4 °C. After centrifugation, the sediment was collected in PBS. The presence of EVs was confirmed with TEM, and the range of particle diameters of the EVs was determined by NTA.

### Biochemical analysis of LmEVs

The protein content of the LmEVs was determined by the Solarbio BCA Protein Assay Kit (Solarbio, #PC0020) following the manufacturer’s instructions. DNA and RNA in the LmEVs were quantified by using the Qubit™ dsDNA HS Assay Kit (YEASEN, #12640ES60) and Qubit™ RNA HS Assay Kit (Invitrogen, #Q32852) following the manufacturer’s instructions, respectively. The contents of protein, DNA, and RNA were normalized by using 1 × 10^9^ particles of vesicles. The amount of LmEVs used in the following experiments was based on the protein content.

### Cellular uptake of LmEVs

MODE-K and IPEC-J2 cells were cultured in DMEM supplemented with 10% fetal bovine serum, 100 U/mL penicillin, and 100 μg/mL streptomycin in an atmosphere of 5% CO_2_ at 37 °C. The cell culture medium was changed to Hank’s balanced salt solution, and after 15 min of incubation, DiI (Sigma, #42364)-labeled LmEVs (10 μg/mL) were added, and the cells were incubated for 6 h. DiI-positive cells were analyzed using a fluorescence microscope.

### Ethics approval and consent to participate in declarations

All animal experimental and sample collection procedures were approved by the Institutional Animal Care and Use Committee of the Huazhong Agricultural University, Hubei, China. In this study, all experimental methods were performed in accordance with the Huazhong Agricultural University of Health Guide for the Care and Use of Laboratory Animals (Approval number HZAUMO-2022-0005).

### Selection of newborn healthy and diarrheal piglets

Newborn piglets used in this study were obtained from Guangxi Yangxiang Co., Ltd. After delivery, 30 piglet litters were selected, and one diarrheal piglet and one healthy piglet were selected from each litter. Piglets with liquid and watery feces for at least 2 consecutive days were classified as diarrheal piglets, while piglets showing no diarrhea or other diseases were classified as healthy piglets^87^. Fecal samples were collected from newborn piglets (30 diarrheal piglets and 30 healthy piglets), of which the ages ranged from 8 to 11 days (Supplementary Table 3). All selected piglets did not receive any antibiotics, probiotics, or prebiotics prior to sample collection. Collected stool samples were divided into two portions: one was immediately snap-frozen in liquid nitrogen, and the other was mixed in a sterile glycerol-PBS solution (15% glycerol) and then frozen in liquid nitrogen. All samples were stored at −80 °C.

To prepare FMT solutions, fecal samples from either diarrheal piglets or healthy piglets were handled under anaerobic conditions. Pooled fecal samples were centrifuged at 100×*g* for 2 min, and the supernatant was filtered through a 70 μm filter^88^.

### Transplantation of fecal microbiota from healthy and diarrheic piglets to GF mice

Twenty GF mice (Kunming, 4 weeks of age) were housed in a sterile isolation environment. Feed and water were available ad libitum. Mice were randomly divided into H-FMT (*n* = 10) or D-FMT (*n* = 10) groups. The H-FMT group was administered 200 μL of a fecal microbiota suspension from healthy piglets by gavage once per day in the morning for one week, and the D-FMT group was administered an equivalent amount of a fecal microbiota suspension from diarrheal piglets. The animals were reared normally in the second week. The body weights of the treated mice were determined each morning during the entire experimental period. Feces samples were collected at the end of the experimental period, and freshly collected stools were quickly immersed in liquid nitrogen and stored at −80 °C. All mice were sacrificed by cervical dislocation. Histological samples from the duodenum, jejunum, ileum, cecum, and colon were rinsed with PBS to remove the intestinal contents and fixed in 4% paraformaldehyde. The remainder of the duodenum, jejunum, ileum, cecum, and colon tissues were collected and stored at −80 °C. Venous blood was collected from the inner corner of the eye. A total of two types of blood samples were collected. One of the samples was whole blood, which was collected in 0.5 mL EDTA anticoagulation tubes for tests of hematological parameters. The second blood sample was left to stand for approximately 30 min at room temperature and then centrifuged for 10 min at 1000 × *g*.

### The protective effect of *L. mucosae* and *L. reuteri* on intestinal damage caused by fecal microbiota in diarrheal piglets

Thirty GF mice (Kunming, 4 weeks of age) were divided into three groups: a D-FMT group (*n* = 10), a D-FMT + *L. reuteri* group (*n* = 10), and a D-FMT + *L. mucosae* group (*n* = 10). Feed and water were provided ad libitum. The D-FMT group was administered by gavage 100 μL of a fecal microbiota suspension derived from diarrheal piglets and 100 μL of PBS daily for the first week, and then 200 μL of PBS daily for the second week. The D-FMT + *L. reuteri* group and D-FMT + *L. mucosae* group were administered by gavage 100 μL of a fecal microbiota suspension of diarrheal piglets and 100 μL of *L. reuteri* or *L. mucosae* bacteria suspension (10^10^ CFU/mL), respectively, daily for the first week, and 200 μL of PBS daily for the second week. The body weights of all mice were determined every morning. The sample collection procedures were the same as those used in the second animal trial experiment.

### The protective effect of *L. mucosae* on intestinal damage caused by ETEC K88

A total of 18 SPF mice (BALB/c, 4 weeks of age) were used in this experiment. The mice were randomly divided into three groups: (1) CON group (*n* = 6); (2) ETEC K88 group (*n* = 6); and (3) ETEC K88 + *L. mucosae* group (*n* = 6). Mice of the ETEC K88 strain (serotype O149:K91, K88ac) were kindly provided by Prof. Wang of China Agricultural University (Beijing, China). Over the first three weeks of the experiment, the mice of the CON and ETEC K88 groups received 200 μL of PBS per day by gavage, and the ETEC K88 + *L. mucosae* group received 200 μL of an *L. mucosae* suspension (10^10^ CFU/mL) per day. Subsequently, 200 μL of PBS was injected intraperitoneally in the CON group, and 200 μL of ETEC K88 (2 × 10^8^ CFU/mL) was injected intraperitoneally in the ETEC K88 and ETEC K88 + *L. mucosae* groups, and samples were collected 5 days later. The body weights of all mice were determined every morning. The sample collection procedures were the same as those used in the second animal trial experiment.

### The protective effect of EVs of *L. mucosae* on intestinal damage caused by ETEC K88

A total of 18 SPF mice (BALB/c, 4 weeks of age) were used in this experiment. The mice were randomly divided into three groups: (1) CON group (*n* = 6); (2) ETEC K88 group (*n* = 6); and (3) ETEC K88 + LmEVs group (*n* = 6). Mice in the CON group were injected intraperitoneally with 200 μL of PBS, and mice in the ETEC K88 and ETEC K88 + LmEVs groups were injected intraperitoneally with 200 μL of ETEC K88 (2 × 10^8^ CFU/mL). Over the next five consecutive days, mice in the CON and ETEC K88 groups received 200 μL of PBS orally daily, and mice in the ETEC K88 + LmEVs group received 50 μg of LmEVs in 200 μL PBS orally daily. The treatment doses of LmEVs were used according to our previous study^89^. The body weights of all mice were recorded every morning. The sample collection procedures were the same as those used in the second animal trial experiment.

### In vivo elimination of macrophages in mice

Elimination of macrophages was performed by intraperitoneal injection with clodronate liposomes (200 μL/mouse) daily for 3 days prior to the experiment and then every 3 days during this experiment as previously described in ref. ^90^. All other experimental steps and sample collection were consistent with the fifth animal trial. This experiment was also divided into three groups: (1) CON-E group (*n* = 6); (2) ETEC K88-E group (*n* = 6); and (3) ETEC K88 + LmEVs-E group (*n* = 6).

### Metagenomic sequencing

The total genomic DNA of fecal samples was extracted using the QIAamp Fast DNA Stool Mini Kit (Qiagen, Tübingen, Germany). DNA concentration and quality was measured with a Qubit 2.0 Fluorometer (Life Technologies, CA, USA). High-quality DNA (1 μg) was used as input material for library construction. Sequencing libraries were generated using NEBNext® Ultra™ DNA Library Prep Kit for Illumina (NEB, USA). The library was sequenced using an Illumina HiSeq platform, and paired-end reads were generated.

### Metagenomic analysis

High-quality reads were obtained for downstream analysis by filtering adapters, low-quality reads, and host genomic DNA contamination from raw sequencing data. Taxonomy annotation was performed with MetaPhlAn3 (version 3.0.7)^91^ and their relative abundance was calculated, followed by the calculation of differential species between diarrhea piglets and healthy piglets with the help of the Linear discriminant analysis Effect Size (LEfSe) method, with a screening criterion of Linear Discriminant Analysis (LDA) score ≥2 and *P* < 0.05. R packages vegan (version 2.5-7) and ade4 (version 1.7-18) were used to calculate the alpha diversity and perform PCoA analyses. The difference in alpha diversity between the two groups was calculated using Wilcox.test. The significance of PCoA was calculated using permutational multivariate analysis of variance (PERMANOVA) based on 9999 permutations. Species with mean relative abundances less than 0.1% were filtered out, and the remaining species were used as an input feature for the random forest classification model with R packages forcats (version 0.5.1), random Forest (version 4.7-1) and splines (version 4.1.2). Species importance was ranked by the mean decrease in model accuracy. The top 20 species were selected as variables in the random forest model, and their area under the curve (AUC) values were calculated with the R package ROCR (version 1.0-11) to evaluate model accuracy. In addition, humann3 (version 3.0.0) was employed to perform functional annotation. The significant differential KO was calculated by Wilcox.test. The significantly differential KO (*p*_adj <0.01) were enriched against KEGG pathways using the R package MicrobiomeProfiler (version 1.1.3).

### 16S rRNA gene amplification, sequencing, and analysis

The V3-V4 region of the 16 S rRNA gene was amplified using universal primers, and the amplified products pooled into equimolar amounts and sequenced on the Illumina MiSeq platform to generate paired-end reads of 300 bp. Raw reads without barcodes were subject to quality control by filtering out low-quality reads. The resulting high-quality reads were imported into QIIME2 as input. In brief, high-quality sequences were first denoised using the DADA2 algorithm and subjected to taxonomy classification using SILVA 132 database. Data analysis was performed on the free online platform of Majorbio Cloud Platform (www.majorbio.com). The microbial results were adjusted by the false discovery rate (FDR) analysis (q < 0.05). Statistical significance was considered at *P* < 0.05.

### RNA extraction, sequencing, and data analysis

Trizol Reagent (Invitrogen) was used to extract the total RNA. The obtained RNA was measured using NanoDrop ND-1000 Spectrophotometer (Thermo, USA). Transcriptome library preparation and sequencing followed^92^. The raw paired-end reads were quality controlled and trimmed by SeqPrep (https://github.com/jstjohn/SeqPrep) and Sickle (https://github.com/najoshi/sickle) using default parameters. The obtained clean reads were compared with the reference genome of mice (http://asia.ensembl.org/Mus_musculus/Info/Index) by TopHat (version 2.0.0) to obtain gene abundance files. To obtain differentially expressed genes between the two groups, differential expression analysis was performed by the R package DESeq2 with a screening threshold of |log2FoldChange| ≥ 1 and *p*_adjust_ < 0.05. After that, KEGG enrichment analysis was performed for differentially expressed genes with Bonferroni-corrected *P* value ≤ 0.05 was required to screen out significantly enriched pathways. KEGG pathway analysis were carried out by KOBAS (http://kobas.cbi.pku.edu.cn/home.do).

### Measurement of the fecal metabolome

We performed the metabolomics detection and analysis by Metabo-Profile (Shanghai, China), then quantified all targeted metabolites with Metabo-Profile Biotechnology (Shanghai) Co., Ltd^93^.

### Hematoxylin and eosin (H&E), periodic acid-Schiff (PAS), and Alcian blue (AB) staining and analysis

H&E, PAS, and AB staining assays were performed^94,95^. Briefly, intestinal tissues fixed with 4% formaldehyde were embedded in paraffin, and sections (5 mm thickness) were obtained and stained with H&E, PAS, or AB. CaseViewer software (version 220 2.2) was used to measure the villus height, crypt depth, and number of goblet cells and glycoproteins and to evaluate histopathology scoring.

### Determination of inflammatory cytokines, LPS, and gut permeability indexes

IL-1β, IL-6, IL-8, TNF-α, LPS, D-LA, and DAO content in the serum, ileum, and colon tissues of mice were measured using enzyme-linked immunosorbent assay (ELISA) kits in accordance with the manufacturer’s instructions (Shanghai Enzyme-linked Biotechnology Co. Ltd., Shanghai, China).

### Western blotting

RIPA protein lysis solution (Roche, Penz-berg, Germany) and BCA protein assay kits (Pierce, Rockford, IL, USA) were used to extract and quantify proteins from intestinal tissues, respectively. The proteins separated by SDS-PAGE gel electrophoresis were transferred to a nitrocellulose membrane (Bio Trace, Pall Co, USA). Then, the nitrocellulose membrane was immersed in a blocking buffer for 2 h and then incubated with the corresponding primary antibodies overnight. The membranes were washed with Tris-buffered-saline with Tween (TBST) and then immersed in a secondary antibody dilution for 2 h. Finally, the protein expression was recorded and analyzed using an Imaging System (Bio-Rad, USA) and Quantity One software (Bio-Rad, USA), respectively. The following antibodies used in the western blot assays were shown as follows: horseradish peroxidase (HRP)-conjugated secondary antibodies (Santa Cruz Biotechnology, goat anti-rabbit (sc-2004; 1:10,000), goat anti-mouse (sc-2005; 1:10,000)), ZO-1 antibody (Abcam, ab221547; 1:1000), Occludin antibody (Abcam, ab216327; 1:1000), phospho-AKT antibody (Abcam, ab8933; 1:1000), AKT antibody (Abcam, ab8805; 1:1000), phospho-NF-κB antibody (Abcam, ab239882; 1:1000), NF-κB antibody (Abcam, ab207297; 1:1000), iNOS antibody (Abcam, ab178945; 1:1000), Arg1 antibody (Abcam, ab92274; 1:1000), β-actin antibody (Sigma-Aldrich, A5441; 1:1000). All blots or gels derive from the same experiment and that were processed in parallel. Original blots are provided in Supplementary Information.

### Statistical analysis

Statistical significance was assessed using a one-way analysis of variance (ANOVA), followed by a post hoc LSD test for pairwise comparisons (SPSS version 20.0 for Windows; SPSS Inc., Chicago, IL, USA). All results were expressed as means ± SEM. Differences were considered statistically significant if *P* < 0.05. Number of replicates used for statistics are noted in the figure legends.

## Supplementary information

Supplementary Information

nr-reporting-summary

## Data Availability

The datasets supporting the conclusions of this article are available in the NCBI Sequence Read Archive (SRA) repository under accession numbers PRJNA847006, PRJNA847017, and PRJNA895872.

## References

[1] Liu L (2016). Global, regional, and national causes of under-5 mortality in 2000-15: an updated systematic analysis with implications for the Sustainable Development Goals. Lancet.

[2] Duggan CP, Jaksic T (2017). Pediatric intestinal failure. N. Engl. J. Med..

[3] Thiagarajah JR (2018). Advances in evaluation of chronic diarrhea in infants. Gastroenterology.

[4] Clasen TF (2015). Interventions to improve water quality for preventing diarrhoea. Cochrane Database Syst. Rev..

[5] Ferdous F (2013). Severity of diarrhea and malnutrition among under five-year-old children in rural Bangladesh. Am. J. Trop. Med. Hyg..

[6] Manary MJ (2010). Perturbed zinc homeostasis in rural 3-5-y-old Malawian children is associated with abnormalities in intestinal permeability attributed to tropical enteropathy. Pediatr. Res..

[7] Ngure FM (2014). Water, sanitation, and hygiene (WASH), environmental enteropathy, nutrition, and early child development: making the links. Ann. N. Y. Acad. Sci..

[8] John CC, Black MM, Nelson CA (2017). Neurodevelopment: the impact of nutrition and inflammation during early to middle childhood in low-resource settings. Pediatrics.

[9] Collaborators GBDU-M. (2021). Global, regional, and national progress towards Sustainable Development Goal 3.2 for neonatal and child health: all-cause and cause-specific mortality findings from the Global Burden of Disease Study 2019. Lancet.

[10] Thiagarajah JR, Donowitz M, Verkman AS (2015). Secretory diarrhoea: mechanisms and emerging therapies. Nat. Rev. Gastroenterol. Hepatol..

[11] Lu Q (2020). Alternations of gut microbiota composition in neonates conceived by assisted reproductive technology and its relation to infant growth. Gut Microbes.

[12] Brodin P (2022). Immune-microbe interactions early in life: a determinant of health and disease long term. Science.

[13] Westrom B, Arevalo Sureda E, Pierzynowska K, Pierzynowski SG, Perez-Cano FJ (2020). The Immature Gut Barrier and Its Importance in Establishing Immunity in Newborn Mammals. Front. Immunol..

[14] Krajmalnik-Brown R, Ilhan ZE, Kang DW, DiBaise JK (2012). Effects of gut microbes on nutrient absorption and energy regulation. Nutr. Clin. Pract..

[15] Kabat AM, Srinivasan N, Maloy KJ (2014). Modulation of immune development and function by intestinal microbiota. Trends Immunol..

[16] Bauer E, Williams BA, Smidt H, Verstegen MW, Mosenthin R (2006). Influence of the gastrointestinal microbiota on development of the immune system in young animals. Curr. Issues Intest. Microbiol..

[17] Ward DV (2016). Metagenomic sequencing with strain-level resolution implicates uropathogenic *E. coli* in necrotizing enterocolitis and mortality in preterm infants. Cell Rep..

[18] Buffie CG, Pamer EG (2013). Microbiota-mediated colonization resistance against intestinal pathogens. Nat. Rev. Immunol..

[19] Pamer EG (2016). Resurrecting the intestinal microbiota to combat antibiotic-resistant pathogens. Science.

[20] Smits LP, Bouter KE, de Vos WM, Borody TJ, Nieuwdorp M (2013). Therapeutic potential of fecal microbiota transplantation. Gastroenterology.

[21] Hvas CL (2019). Fecal microbiota transplantation is superior to fidaxomicin for treatment of recurrent *Clostridium difficile* infection. Gastroenterology.

[22] Haifer C (2022). Lyophilised oral faecal microbiota transplantation for ulcerative colitis (LOTUS): a randomised, double-blind, placebo-controlled trial. Lancet Gastroenterol. Hepatol..

[23] Lima SF (2022). Transferable immunoglobulin A-coated *Odoribacter splanchnicus* in responders to fecal microbiota transplantation for ulcerative colitis limits colonic inflammation. Gastroenterology.

[24] Hu J (2018). A microbiota-derived bacteriocin targets the host to confer diarrhea resistance in early-weaned piglets. Cell Host Microbe.

[25] Lemon KP, Armitage GC, Relman DA, Fischbach MA (2012). Microbiota-targeted therapies: an ecological perspective. Sci. Transl. Med..

[26] Yanez-Mo M (2015). Biological properties of extracellular vesicles and their physiological functions. J. Extracell Vesicles.

[27] Kim JH, Lee J, Park J, Gho YS (2015). Gram-negative and Gram-positive bacterial extracellular vesicles. Semin. Cell Dev. Biol..

[28] Schwechheimer C, Kuehn MJ (2015). Outer-membrane vesicles from Gram-negative bacteria: biogenesis and functions. Nat. Rev. Microbiol..

[29] Rivera J (2010). Bacillus anthracis produces membrane-derived vesicles containing biologically active toxins. Proc. Natl Acad. Sci. USA.

[30] Olaya-Abril A (2014). Characterization of protective extracellular membrane-derived vesicles produced by *Streptococcus pneumoniae*. J. Proteomics.

[31] Brown L, Kessler A, Cabezas-Sanchez P, Luque-Garcia JL, Casadevall A (2014). Extracellular vesicles produced by the Gram-positive bacterium *Bacillus subtilis* are disrupted by the lipopeptide surfactin. Mol. Microbiol..

[32] Jiang Y, Kong Q, Roland KL, Curtiss R (2014). Membrane vesicles of *Clostridium perfringens* type A strains induce innate and adaptive immunity. Int. J. Med. Microbiol..

[33] Dominguez Rubio AP (2017). *Lactobacillus casei* BL23 produces microvesicles carrying proteins that have been associated with its probiotic effect. Front. Microbiol..

[34] Li M (2017). Lactobacillus-derived extracellular vesicles enhance host immune responses against vancomycin-resistant enterococci. BMC Microbiol..

[35] Dean SN, Leary DH, Sullivan CJ, Oh E, Walper SA (2019). Isolation and characterization of Lactobacillus-derived membrane vesicles. Sci. Rep..

[36] Wang Z (2021). Xylan alleviates dietary fiber deprivation-induced dysbiosis by selectively promoting Bifidobacterium pseudocatenulatum in pigs. Microbiome.

[37] Li N (2020). Spatial heterogeneity of bacterial colonization across different gut segments following inter-species microbiota transplantation. Microbiome.

[38] Zhou X (2022). Intestinal accumulation of microbiota-produced succinate caused by loss of microRNAs leads to diarrhea in weanling piglets. Gut Microbes.

[39] da Cruz Gouveia MA, Lins MTC, da Silva GAP (2020). Acute diarrhea with blood: diagnosis and drug treatment. J. Pediatr..

[40] van Baarlen P, Wells JM, Kleerebezem M (2013). Regulation of intestinal homeostasis and immunity with probiotic lactobacilli. Trends Immunol..

[41] Zhang Y, Tan P, Zhao Y, Ma X (2022). Enterotoxigenic *Escherichia coli*: intestinal pathogenesis mechanisms and colonization resistance by gut microbiota. Gut Microbes.

[42] Clairfeuille T (2020). Structure of the essential inner membrane lipopolysaccharide-PbgA complex. Nature.

[43] Xiao Z (2021). A potential probiotic for diarrhea: *Clostridium tyrobutyricum* protects against LPS-induced epithelial dysfunction via IL-22 produced by Th17 cells in the ileum. Front. Immunol..

[44] Kostic AD (2013). Fusobacterium nucleatum potentiates intestinal tumorigenesis and modulates the tumor-immune microenvironment. Cell Host Microbe.

[45] Brennan CA, Garrett WS (2019). Fusobacterium nucleatum - symbiont, opportunist and oncobacterium. Nat. Rev. Microbiol..

[46] Turnbaugh PJ (2006). An obesity-associated gut microbiome with increased capacity for energy harvest. Nature.

[47] Wang Z (2021). Gut microbiota modulates the inflammatory response and cognitive impairment induced by sleep deprivation. Mol. Psychiatry.

[48] Huang Z (2020). Faecal microbiota transplantation from metabolically compromised human donors accelerates osteoarthritis in mice. Ann. Rheum. Dis..

[49] Ridaura VK (2013). Gut microbiota from twins discordant for obesity modulate metabolism in mice. Science.

[50] Ma C (2018). Cow-to-mouse fecal transplantations suggest intestinal microbiome as one cause of mastitis. Microbiome.

[51] Mazmanian SK, Round JL, Kasper DL (2008). A microbial symbiosis factor prevents intestinal inflammatory disease. Nature.

[52] Yang J (2020). Landscapes of bacterial and metabolic signatures and their interaction in major depressive disorders. Sci. Adv..

[53] Zierer J (2018). The fecal metabolome as a functional readout of the gut microbiome. Nat. Genet..

[54] Wang S, Suh JH, Zheng X, Wang Y, Ho CT (2017). Identification and quantification of potential anti-inflammatory hydroxycinnamic acid amides from Wolfberry. J. Agric. Food Chem..

[55] Ding S, Jiang H, Fang J, Liu G (2021). Regulatory effect of resveratrol on inflammation induced by lipopolysaccharides via reprograming intestinal microbes and ameliorating serum metabolism profiles. Front. Immunol..

[56] Chifiriuc MC (2009). In vivo experimental model for the study of the influence of subinhibitory concentrations of phenyllactic acid on *Staphylococcus aureus* pathogenicity. Roum. Arch. Microbiol. Immunol..

[57] Bosco MC (2003). Macrophage activating properties of the tryptophan catabolite picolinic acid. Adv. Exp. Med. Biol..

[58] Kotloff KL (2019). The incidence, aetiology, and adverse clinical consequences of less severe diarrhoeal episodes among infants and children residing in low-income and middle-income countries: a 12-month case-control study as a follow-on to the Global Enteric Multicenter Study (GEMS). Lancet Glob. Health.

[59] Moore SR (2010). Prolonged episodes of acute diarrhea reduce growth and increase risk of persistent diarrhea in children. Gastroenterology.

[60] Kinashi Y, Hase K (2021). Partners in leaky gut syndrome: intestinal dysbiosis and autoimmunity. Front. Immunol..

[61] Afonina IS, Zhong Z, Karin M, Beyaert R (2017). Limiting inflammation-the negative regulation of NF-kappaB and the NLRP3 inflammasome. Nat. Immunol..

[62] Schieber AM (2015). Disease tolerance mediated by microbiome *E. coli* involves inflammasome and IGF-1 signaling. Science.

[63] Tilg H, Zmora N, Adolph TE, Elinav E (2020). The intestinal microbiota fuelling metabolic inflammation. Nat. Rev. Immunol..

[64] Martel J (2022). Gut barrier disruption and chronic disease. Trends Endocrinol. Metab..

[65] Thomas H (2017). IBD: FMT induces clinical remission in ulcerative colitis. Nat. Rev. Gastroenterol. Hepatol..

[66] Moayyedi P (2015). Fecal microbiota transplantation induces remission in patients with active ulcerative colitis in a randomized controlled trial. Gastroenterology.

[67] Lleal M (2019). A single faecal microbiota transplantation modulates the microbiome and improves clinical manifestations in a rat model of colitis. EBioMedicine.

[68] Kim JK, Lee KE, Lee SA, Jang HM, Kim DH (2020). Interplay between human gut bacteria *Escherichia coli* and *Lactobacillus mucosae* in the occurrence of neuropsychiatric disorders in mice. Front. Immunol..

[69] Wu H (2020). Lactobacillus reuteri maintains intestinal epithelial regeneration and repairs damaged intestinal mucosa. Gut Microbes.

[70] Buffie CG (2015). Precision microbiome reconstitution restores bile acid mediated resistance to *Clostridium difficile*. Nature.

[71] Thaiss CA, Zmora N, Levy M, Elinav E (2016). The microbiome and innate immunity. Nature.

[72] Macia L, Nanan R, Hosseini-Beheshti E, Grau GE (2019). Host- and microbiota-derived extracellular vesicles, immune function, and disease development. Int. J. Mol. Sci..

[73] Diaz-Garrido N, Badia J, Baldoma L (2021). Microbiota-derived extracellular vesicles in interkingdom communication in the gut. J. Extracell Vesicles.

[74] Kang CS (2013). Extracellular vesicles derived from gut microbiota, especially Akkermansia muciniphila, protect the progression of dextran sulfate sodium-induced colitis. PLoS ONE.

[75] Kim JH (2016). Extracellular vesicle-derived protein from *Bifidobacterium longum* alleviates food allergy through mast cell suppression. J. Allergy Clin. Immunol..

[76] West CL (2020). Microvesicles from *Lactobacillus reuteri* (DSM-17938) completely reproduce modulation of gut motility by bacteria in mice. PLoS ONE.

[77] Pang Y (2022). Extracellular membrane vesicles from *Limosilactobacillus reuteri* strengthen the intestinal epithelial integrity, modulate cytokine responses and antagonize activation of TRPV1. Front. Microbiol..

[78] Hu R (2021). Lactobacillus reuteri-derived extracellular vesicles maintain intestinal immune homeostasis against lipopolysaccharide-induced inflammatory responses in broilers. J. Anim. Sci. Biotechnol..

[79] Dargenio VN (2022). Use of *Limosilactobacillus reuteri* DSM 17938 in paediatric gastrointestinal disorders: an updated review. Benef. Microbes.

[80] Na YR, Stakenborg M, Seok SH, Matteoli G (2019). Macrophages in intestinal inflammation and resolution: a potential therapeutic target in IBD. Nat. Rev. Gastroenterol. Hepatol..

[81] Viola MF, Boeckxstaens G (2021). Niche-specific functional heterogeneity of intestinal resident macrophages. Gut.

[82] Macias-Ceja DC (2019). Succinate receptor mediates intestinal inflammation and fibrosis. Mucosal Immunol..

[83] Quail DF (2016). The tumor microenvironment underlies acquired resistance to CSF-1R inhibition in gliomas. Science.

[84] Liu L (2020). Progranulin inhibits LPS-induced macrophage M1 polarization via NF-small ka, CyrillicB and MAPK pathways. BMC Immunol..

[85] Vergadi E, Ieronymaki E, Lyroni K, Vaporidi K, Tsatsanis C (2017). Akt signaling pathway in macrophage activation and M1/M2 polarization. J. Immunol..

[86] Liu L, Liang L, Yang C, Zhou Y, Chen Y (2021). Extracellular vesicles of *Fusobacterium nucleatum* compromise intestinal barrier through targeting RIPK1-mediated cell death pathway. Gut Microbes.

[87] Hermann-Bank ML (2015). Characterization of the bacterial gut microbiota of piglets suffering from new neonatal porcine diarrhoea. BMC Vet. Res..

[88] Wu Z (2021). Gut microbiota from green tea polyphenol-dosed mice improves intestinal epithelial homeostasis and ameliorates experimental colitis. Microbiome.

[89] Ma L (2022). *Clostridium butyricum* and its derived extracellular vesicles modulate gut homeostasis and ameliorate acute experimental colitis. Microbiol. Spectr..

[90] Li L (2021). IL-25-induced shifts in macrophage polarization promote development of beige fat and improve metabolic homeostasis in mice. PLoS Biol..

[91] Beghini F (2021). Integrating taxonomic, functional, and strain-level profiling of diverse microbial communities with bioBakery 3. eLife.

[92] Tao S (2021). N-acyl-homoserine lactones may affect the gut health of low-birth-weight piglets by altering intestinal epithelial cell barrier function and amino acid metabolism. J. Nutr..

[93] Wu Z (2021). Intestinal microbiota and serum metabolic profile responded to two nutritional different diets in mice. Front. Nutr..

[94] Tao S (2014). A high-concentrate diet induced colonic epithelial barrier disruption is associated with the activating of cell apoptosis in lactating goats. BMC Vet. Res..

[95] Tao S, Bai Y, Li T, Li N, Wang J (2019). Original low birth weight deteriorates the hindgut epithelial barrier function in pigs at the growing stage. FASEB J..

