# Supplementary material for: Limosilactobacillus mucosae-derived extracellular vesicles modulates macrophage phenotype and orchestrates gut homeostasis in a diarrheal piglet model
Source: NPJ Biofilms Microbiomes. 2023 Jun 6;9:33. doi: 10.1038/s41522-023-00403-6 (PMC10244441; doi:10.1038/s41522-023-00403-6)

Supplementary materials for

***Limosilactobacillus mucosae*-derived extracellular vesicles modulates macrophage phenotype and orchestrates gut homeostasis in a diarrheal piglet model**

Jingjing Li[1,3], Shuaifei Feng[1,3], Zhenyu Wang[2,3], Jinhui He[1], Zeyue Zhang[1], Huicong Zou[1], Zhifeng Wu[1], Xiangdong Liu[1,*], Hong Wei[1,*], Shiyu Tao[1,*]

[1]College of Animal Sciences and Technology, Huazhong Agricultural University, Wuhan, China 430070

[2]State Key Laboratory of Animal Nutrition, College of Animal Science and Technology, China Agricultural University, No. 2 Yuanmingyuan West Road, Beijing 100193, China

[3]These authors contributed equally to the present work.

[*]**Address for reprint requests and other correspondence:**

Xiangdong Liu, College of Animal Sciences and Technology, Huazhong Agricultural University, Wuhan, China 430070 (e-mail: liuxiangdong@mail.hzau.edu.cn).

Hong Wei, College of Animal Sciences and Technology, Huazhong Agricultural University, Wuhan, China 430070 (e-mail: weihong63528@163.com).

Shiyu Tao, College of Animal Sciences and Technology, Huazhong Agricultural University, Wuhan, China 430070 (e-mail: sytao@mail.hzau.edu.cn).

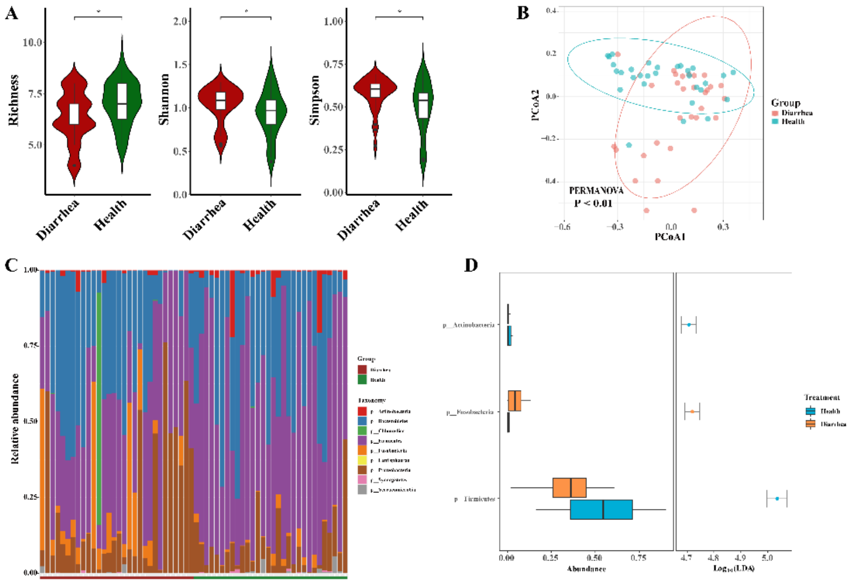

Supplementary Figure 1: Gut microbiome characteristics at the phylum level, related to Figure 2

A: Comparison of gut microorganisms at the phylum level alpha diversity (Richness, Shannon, and Simpson) between the two groups. B: PCoA analysis of gut microbiome at the phylum level in the two groups, data were analyzed using PERMANOVA. C: Histogram of the composition of gut microbiome at the phylum level in the two groups. D: Differential bacteria at the phylum level between the two groups. n = 30. Mann-Whitney *U* test was performed (A). * $P < 0.05$: Diarrhea group versus Health group.

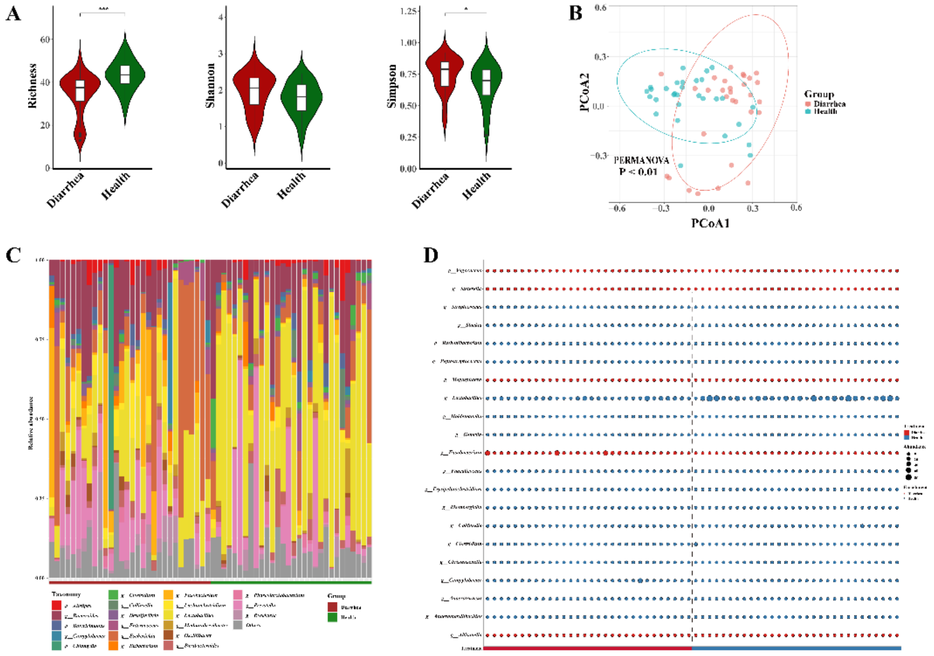

Supplementary Figure 2: Gut microbiome characteristics at the genus level, related to Figure 2

A: Comparison of gut microorganisms at the genus level alpha diversity (Richness, Shannon, and Simpson) between the two groups. B: PCoA analysis of gut microbiome at the genus level in the two groups, data were analyzed using PERMANOVA. C: Histogram of the composition of gut microbiome at the genus level in the two groups. D: Differential bacteria at the genus level between the two groups. n = 30. Mann-Whitney *U* test was performed (A). * $P < 0.05$, *** $P < 0.001$: Diarrhea group versus Health group.

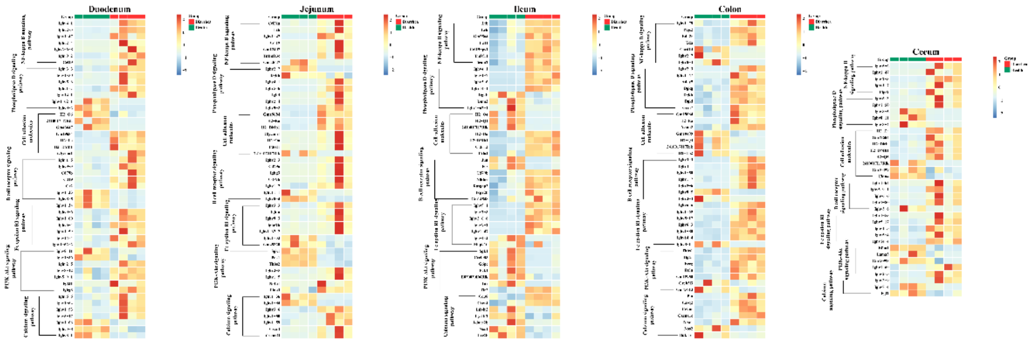

Supplementary Figure 3: Heatmap of differential genes related to immunity, inflammation, and cell survival among the five intestinal segments, related to Figure 3

The color of the corresponding grid of each gene is related to the expression level. The higher the expression, the redder the color. n = 4.

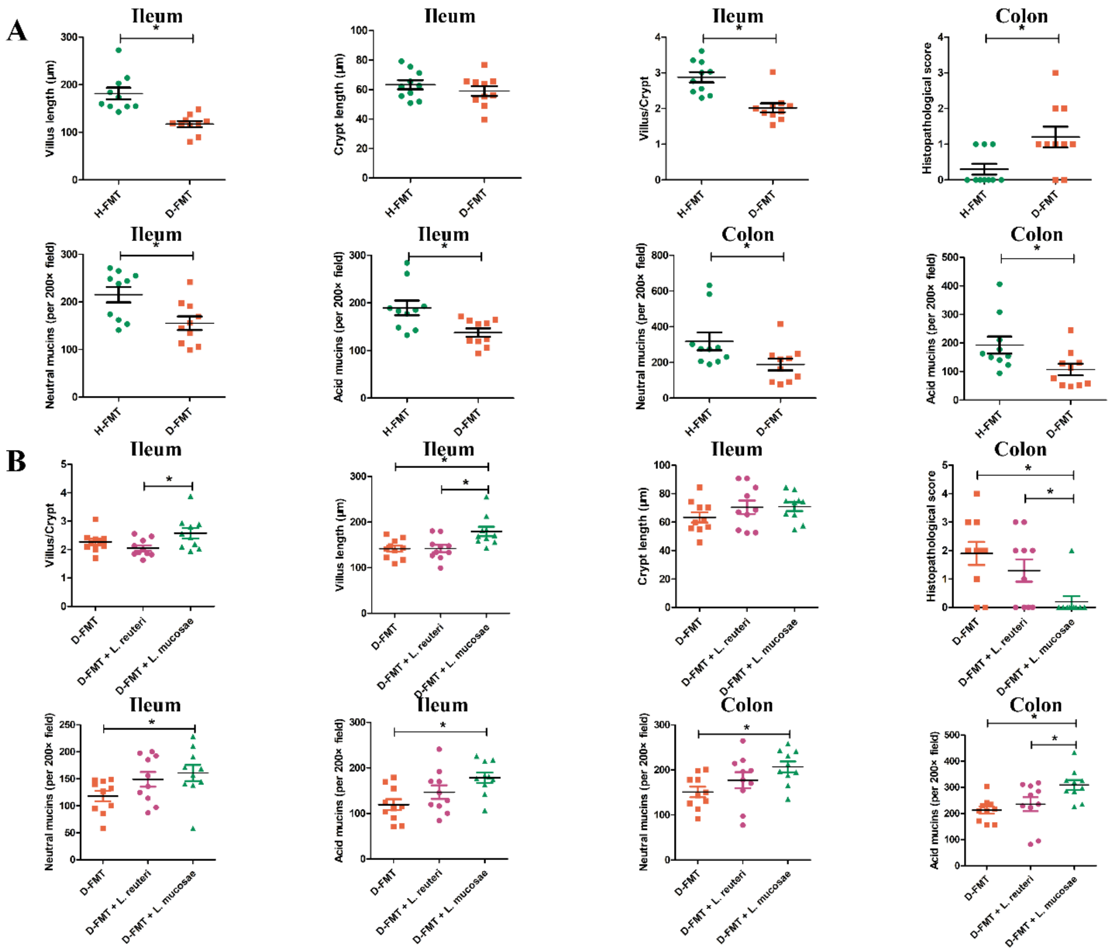

Supplementary Figure 4: Morphological statistics, related to Figure 4 and 5

A: The measurement results of villus length, crypt length, villus\crypt of the ileum, the measurement results of the histopathological score of the colon, and the measurement results of neutral mucins and acid mucins of the ileum and colon of the second animal trial. B: The measurement results of villus length, crypt length, villus\crypt of the ileum, the measurement results of the histopathological score of the colon, and the measurement results of neutral mucins, and Acid mucins of the ileum and colon of the third animal trial. Data are expressed as the means ± SEM (A and B) and one-way ANOVA was performed, followed by LSD's test (A and B). * $P < 0.05$ for A: H-FMT group versus D-FMT group; n = 10. * $P < 0.05$.

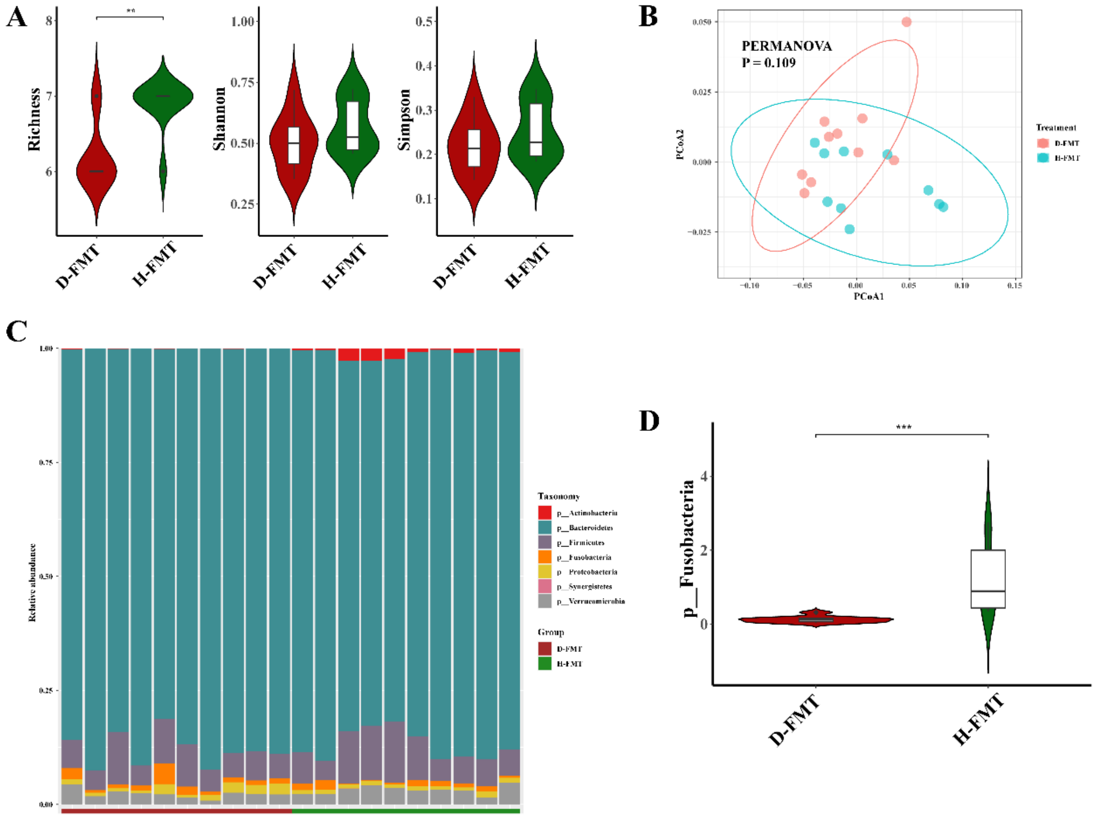

Supplementary Figure 5: Gut microbiome characteristics at the phylum level, related to Figure 4

A: Comparison of alpha diversity (Richness, Shannon, and Simpson) of gut microbiome at the phylum level between the two groups. B: PCoA analysis of gut microbiome at the phylum level between the two groups, data were analyzed using PERMANOVA. C: Histogram of the composition of the two groups of intestinal microbiomes at the phylum level. D: Differential bacteria at the phylum level between the two groups. n = 10. Mann-Whitney *U* test was performed (A). ** $P < 0.01$; *** $P < 0.001$: H-FMT group versus D-FMT group.

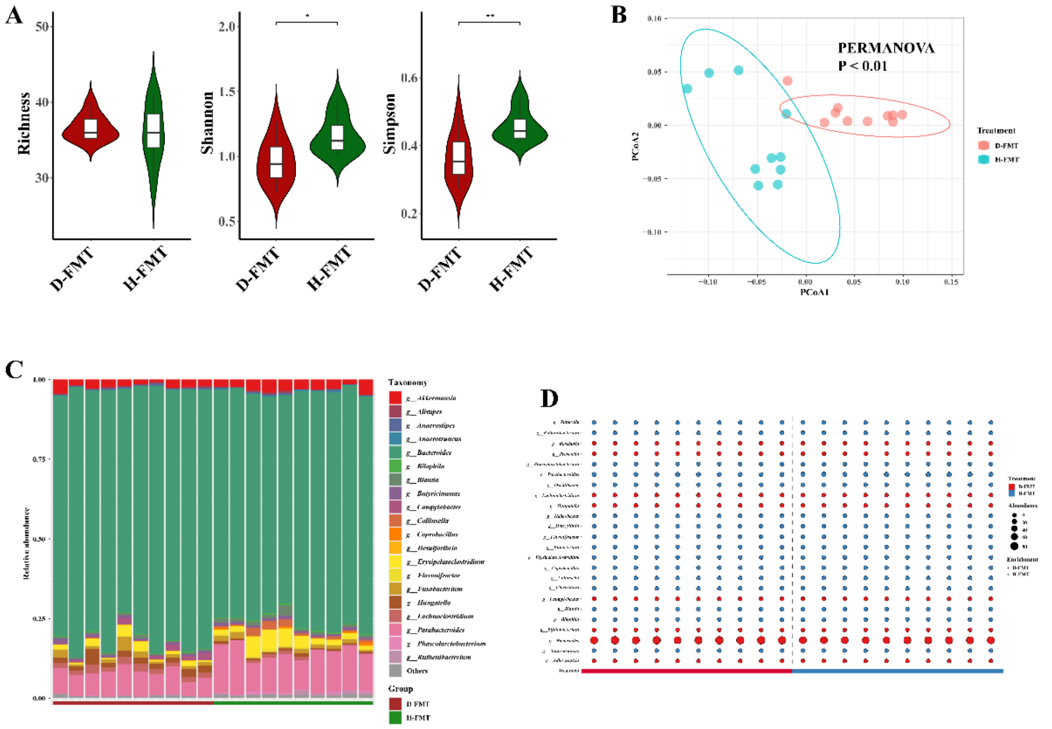

Supplementary Figure 6: Gut microbiome characteristics at the genus level, related to Figure 4

A: Comparison of alpha diversity (Richness, Shannon, and Simpson) of gut microbiome at the genus level between the two groups. B: PCoA analysis of gut microbiome at the genus level between the two groups, data were analyzed using PERMANOVA. C: Histogram of the composition of the two groups of intestinal microbiomes at the genus level. D: Differential bacteria at the genus level between the two groups. n = 10. Mann-Whitney *U* test was performed (A). * $P < 0.05$; ** $P < 0.01$: H-FMT group versus D-FMT group.

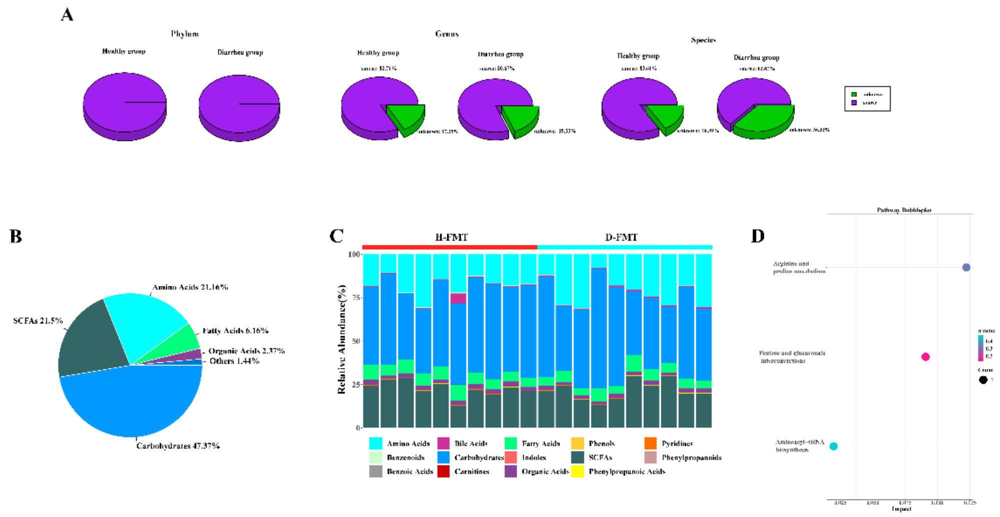

Supplementary Figure 7: Microbiome traceability and metabolites composition characteristics, related to Figure 4

A: Traceability analysis of microbiome in GF mice at the phylum, genus, and species levels. B: Pie charts of the mean abundance composition ratio of each metabolite type in all samples. C: Stacked histogram of the relative abundance of each metabolite type in each sample. D: KEGG pathways.

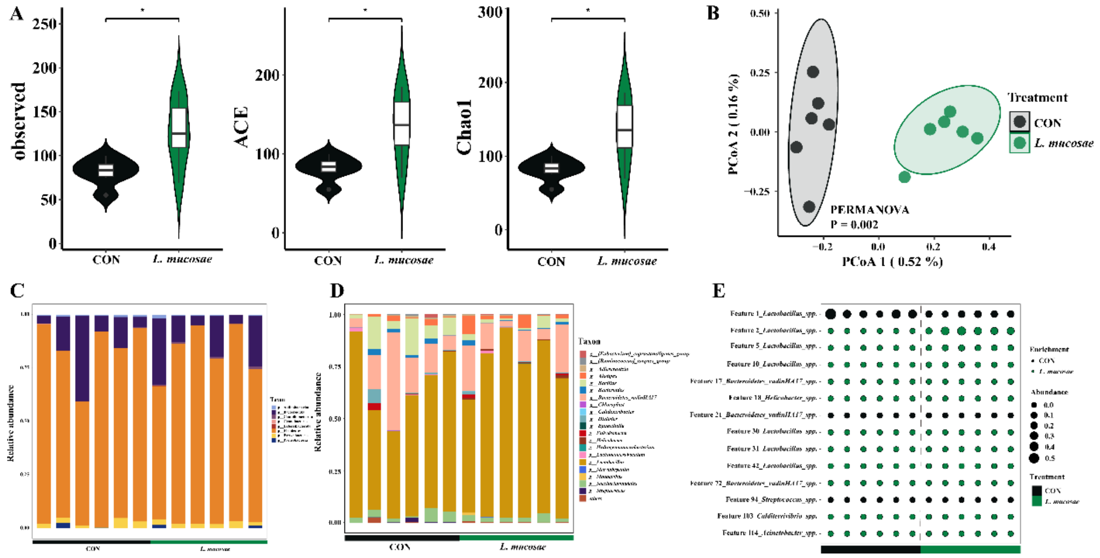

Supplementary Figure 8: Intestinal microbiota characteristics remodeled by *L. mucosae*, related to Figure 6

A: Comparison of alpha diversity (Observed, ACE, and Chao1) of gut microbiome at the ASV level between the two groups. B: PCoA analysis of gut microbiome at the ASV level between the two groups, data were analyzed using PERMANOVA. C: Histogram of the composition of the two groups of intestinal microbiomes at the phylum level. D: Histogram of the composition of the two groups of intestinal microbiomes at the genus level. E: Differential bacteria at the ASV level between the two groups. n = 10. Mann-Whitney U test was performed (A). * $P < 0.05$.

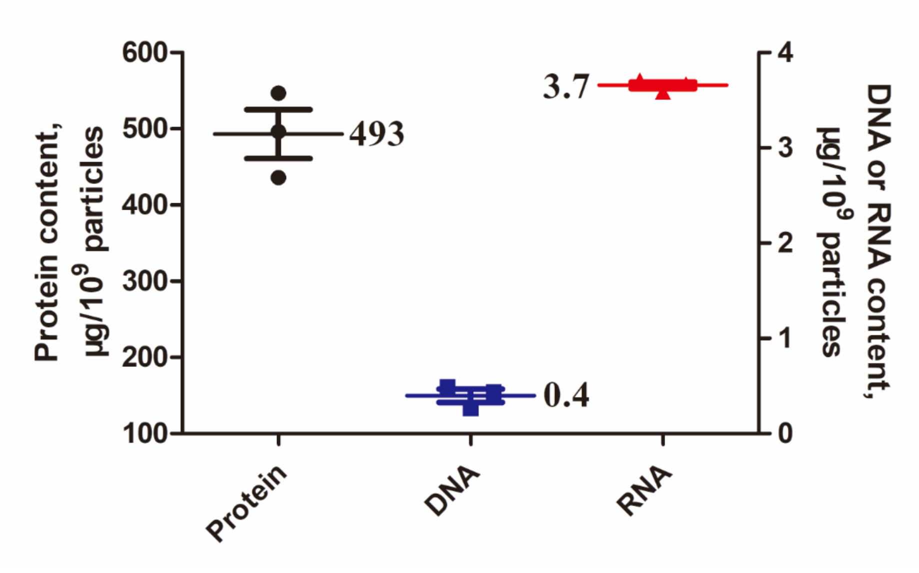

Supplementary Figure 9: Biochemical analyses of LmEVs, related to Figure 7.

Quantifications of DNA, RNA, and protein in the LmEVs. n = 3.

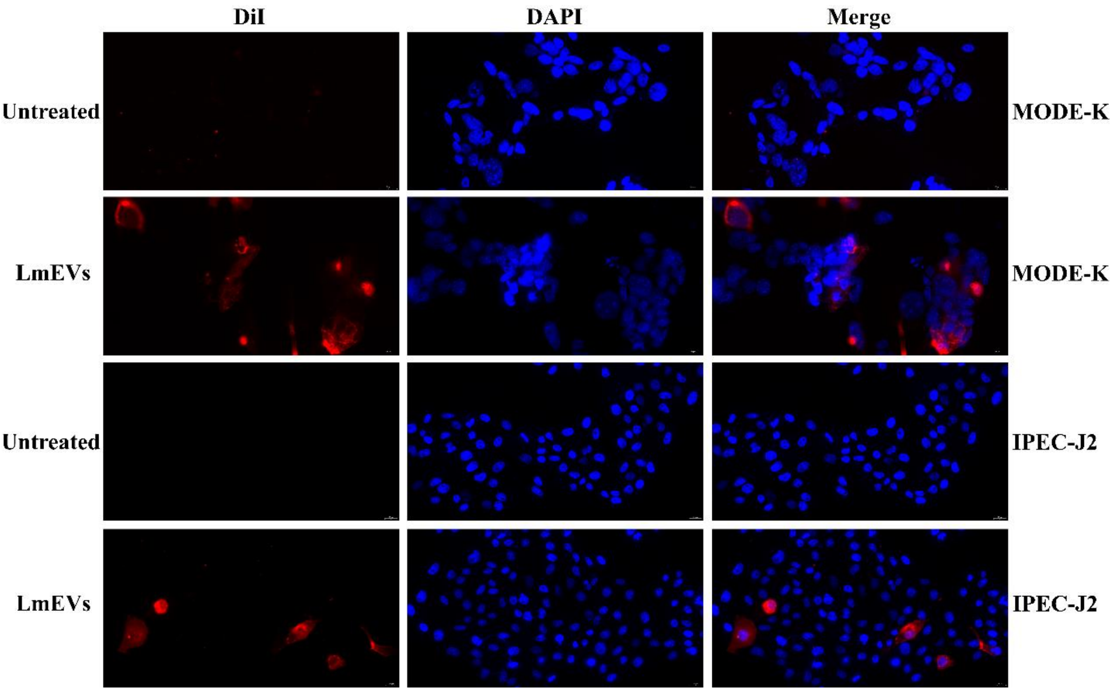

Supplementary Figure 10: Fluorescence microscope showed that LmEVs were internalized by MODE-K and IPEC-J2 cells, related to Figure 7

The MODE-K or IPEC-J2 cells were co-incubated with medium (row 1 or 3) and DiI-labeled LmEVs (row 2 or 4) for 6 h at 37 °C. LmEVs were labeled with DiI (red signal), and the cell nucleus was stained with DAPI (blue signal).

Supplementary Table 1: The relative abundance of differential KOs in healthy and diarrheal newborn piglets

KO	Mean (Diarrheic newborn piglets)	SD (Diarrheic newborn piglets)	Mean (Healthy newborn piglets)	SD (Healthy newborn piglets)	Enrichment	P_adjust
K01704	0.00	0.00	0.00	0.00	Diarrhea	0.00
K03307	0.00	0.00	0.00	0.00	Diarrhea	0.00
K07862	0.00	0.00	0.00	0.00	Diarrhea	0.00
K01999	0.00	0.00	0.00	0.00	Diarrhea	0.00
K02825	0.00	0.00	0.00	0.00	Health	0.00
K02843	0.00	0.00	0.00	0.00	Diarrhea	0.00
K03271	0.00	0.00	0.00	0.00	Diarrhea	0.00
K07652	0.00	0.00	0.00	0.00	Health	0.00
K10439	0.00	0.00	0.00	0.00	Diarrhea	0.00
K16323	0.00	0.00	0.00	0.00	Health	0.00
K18692	0.00	0.00	0.00	0.00	Health	0.00
K19002	0.00	0.00	0.00	0.00	Health	0.00
K01069	0.00	0.00	0.00	0.00	Diarrhea	0.00
K07507	0.00	0.00	0.00	0.00	Diarrhea	0.00
K00016	0.00	0.00	0.00	0.00	Health	0.00
K00133	0.00	0.00	0.00	0.00	Diarrhea	0.00
K02106	0.00	0.00	0.00	0.00	Diarrhea	0.00
K00712	0.00	0.00	0.00	0.00	Health	0.00
K01627	0.00	0.00	0.00	0.00	Diarrhea	0.00
K02806	0.00	0.00	0.00	0.00	Diarrhea	0.00
K03073	0.00	0.00	0.00	0.00	Health	0.00
K08303	0.00	0.00	0.00	0.00	Diarrhea	0.00
K10440	0.00	0.00	0.00	0.00	Diarrhea	0.00
K16511	0.00	0.00	0.00	0.00	Health	0.00
K16899	0.00	0.00	0.00	0.00	Health	0.00
K19005	0.00	0.00	0.00	0.00	Health	0.00
K02015	0.00	0.00	0.00	0.00	Diarrhea	0.00
K02916	0.00	0.00	0.00	0.00	Health	0.00
K00783	0.00	0.00	0.00	0.00	Health	0.00
K01464	0.00	0.00	0.00	0.00	Diarrhea	0.00
K01754	0.00	0.00	0.00	0.00	Diarrhea	0.00
K07304	0.00	0.00	0.00	0.00	Health	0.00
K07402	0.00	0.00	0.00	0.00	Diarrhea	0.00
K22397	0.00	0.00	0.00	0.00	Diarrhea	0.00
K00677	0.00	0.00	0.00	0.00	Diarrhea	0.00
K01515	0.00	0.00	0.00	0.00	Diarrhea	0.00
K02790	0.00	0.00	0.00	0.00	Diarrhea	0.00
K02791	0.00	0.00	0.00	0.00	Diarrhea	0.00
K02859	0.00	0.00	0.00	0.00	Health	0.00

K03273	0.00	0.00	0.00	0.00	Diarrhea	0.00
K03293	0.00	0.00	0.00	0.00	Health	0.00
K03569	0.00	0.00	0.00	0.00	Diarrhea	0.00
K13678	0.00	0.00	0.00	0.00	Health	0.00
K07002	0.00	0.00	0.00	0.00	Health	0.00
K14761	0.00	0.00	0.00	0.00	Diarrhea	0.00
K09787	0.00	0.00	0.00	0.00	Health	0.00
K03367	0.00	0.00	0.00	0.00	Health	0.00
K04758	0.00	0.00	0.00	0.00	Diarrhea	0.00
K01771	0.00	0.00	0.00	0.00	Health	0.00
K12942	0.00	0.00	0.00	0.00	Diarrhea	0.00
K15584	0.00	0.00	0.00	0.00	Diarrhea	0.00
K19268	0.00	0.00	0.00	0.00	Diarrhea	0.00
K02112	0.00	0.00	0.00	0.00	Health	0.00
K02824	0.00	0.00	0.00	0.00	Diarrhea	0.00
K02913	0.04	0.03	0.09	0.04	Health	0.00
K00691	0.00	0.00	0.00	0.00	Health	0.00
K01951	0.00	0.00	0.00	0.00	Health	0.00
K01034	0.00	0.00	0.00	0.00	Diarrhea	0.00
K01597	0.00	0.00	0.00	0.00	Health	0.00
K02063	0.00	0.00	0.00	0.00	Diarrhea	0.00
K02760	0.00	0.00	0.00	0.00	Diarrhea	0.00
K03074	0.00	0.00	0.00	0.00	Diarrhea	0.00
K06041	0.00	0.00	0.00	0.00	Diarrhea	0.00
K13653	0.00	0.00	0.00	0.00	Health	0.00
K07034	0.00	0.00	0.00	0.00	Diarrhea	0.00
K00297	0.00	0.00	0.00	0.00	Diarrhea	0.00
K00939	0.00	0.00	0.00	0.00	Health	0.00
K02968	0.00	0.00	0.00	0.00	Health	0.00
K06187	0.00	0.00	0.00	0.00	Health	0.00
K03095	0.00	0.00	0.00	0.00	Health	0.00
K05339	0.00	0.00	0.00	0.00	Health	0.00
K07712	0.00	0.00	0.00	0.00	Diarrhea	0.00
K09705	0.00	0.00	0.00	0.00	Health	0.00
K22719	0.00	0.00	0.00	0.00	Diarrhea	0.00
K00054	0.00	0.00	0.00	0.00	Health	0.00
K03151	0.00	0.00	0.00	0.00	Health	0.00
K12952	0.00	0.00	0.00	0.00	Health	0.00
K03536	0.00	0.00	0.00	0.00	Health	0.00
K05810	0.00	0.00	0.00	0.00	Diarrhea	0.00
K06917	0.00	0.00	0.00	0.00	Diarrhea	0.00
K08094	0.00	0.00	0.00	0.00	Health	0.00
K14392	0.00	0.00	0.00	0.00	Diarrhea	0.00
K01489	0.00	0.00	0.00	0.00	Diarrhea	0.00

K01685	0.00	0.00	0.00	0.00	Diarrhea	0.00
K01919	0.00	0.00	0.00	0.00	Health	0.00
K02055	0.00	0.00	0.00	0.00	Diarrhea	0.00
K02446	0.00	0.00	0.00	0.00	Diarrhea	0.00
K07009	0.00	0.00	0.00	0.00	Health	0.00
K07052	0.00	0.00	0.00	0.00	Health	0.00
K07177	0.00	0.00	0.00	0.00	Health	0.00
K16898	0.00	0.00	0.00	0.00	Health	0.00
K05823	0.00	0.00	0.00	0.00	Health	0.00
K16213	0.00	0.00	0.00	0.00	Health	0.00
K01689	0.00	0.00	0.00	0.00	Health	0.00
K09698	0.00	0.00	0.00	0.00	Health	0.00
K14205	0.00	0.00	0.00	0.00	Health	0.00
K04086	0.00	0.00	0.00	0.00	Health	0.00
K00111	0.00	0.00	0.00	0.00	Diarrhea	0.00
K22736	0.00	0.00	0.00	0.00	Health	0.00
K07574	0.00	0.00	0.00	0.00	Diarrhea	0.00
K08963	0.00	0.00	0.00	0.00	Diarrhea	0.00
K01035	0.00	0.00	0.00	0.00	Diarrhea	0.00
K01962	0.00	0.00	0.00	0.00	Diarrhea	0.00
K03482	0.00	0.00	0.00	0.00	Diarrhea	0.00
K07019	0.00	0.00	0.00	0.00	Diarrhea	0.00
K02020	0.00	0.00	0.00	0.00	Diarrhea	0.00
K02959	0.00	0.00	0.00	0.00	Health	0.00
K18640	0.00	0.00	0.00	0.00	Health	0.00
K03664	0.00	0.00	0.00	0.00	Health	0.00
K07015	0.00	0.00	0.00	0.00	Health	0.00
K00027	0.00	0.00	0.00	0.00	Diarrhea	0.00
K00041	0.00	0.00	0.00	0.00	Diarrhea	0.00
K07447	0.00	0.00	0.00	0.00	Health	0.00
K09384	0.00	0.00	0.00	0.00	Health	0.00
K07729	0.00	0.00	0.00	0.00	Health	0.00
K03800	0.00	0.00	0.00	0.00	Health	0.00
K09824	0.00	0.00	0.00	0.00	Diarrhea	0.00
K00053	0.00	0.00	0.00	0.00	Diarrhea	0.00
K01938	0.00	0.00	0.00	0.00	Health	0.00
K16209	0.00	0.00	0.00	0.00	Health	0.00
K21567	0.00	0.00	0.00	0.00	Health	0.00
K03789	0.00	0.00	0.00	0.00	Diarrhea	0.00
K01995	0.00	0.00	0.00	0.00	Diarrhea	0.00
K03565	0.00	0.00	0.00	0.00	Health	0.00
K03628	0.00	0.00	0.00	0.00	Diarrhea	0.00
K10914	0.00	0.00	0.00	0.00	Diarrhea	0.00
K00613	0.00	0.00	0.00	0.00	Health	0.00

K00773	0.00	0.00	0.00	0.00	Diarrhea	0.00
K00789	0.00	0.00	0.00	0.00	Health	0.00
K01756	0.00	0.00	0.00	0.00	Health	0.00
K02654	0.00	0.00	0.00	0.00	Diarrhea	0.00
K02535	0.00	0.00	0.00	0.00	Diarrhea	0.00
K03823	0.00	0.00	0.00	0.00	Health	0.00
K04488	0.00	0.00	0.00	0.00	Diarrhea	0.00
K07668	0.00	0.00	0.00	0.00	Health	0.00
K07238	0.00	0.00	0.00	0.00	Diarrhea	0.00
K07696	0.00	0.00	0.00	0.00	Health	0.00
K08316	0.00	0.00	0.00	0.00	Diarrhea	0.00
K06902	0.00	0.00	0.00	0.00	Health	0.00
K06956	0.00	0.00	0.00	0.00	Diarrhea	0.00
K03324	0.00	0.00	0.00	0.00	Diarrhea	0.00
K06198	0.00	0.00	0.00	0.00	Health	0.00
K00366	0.00	0.00	0.00	0.00	Diarrhea	0.00
K01625	0.00	0.00	0.00	0.00	Diarrhea	0.00
K01933	0.00	0.00	0.00	0.00	Health	0.00
K02053	0.00	0.00	0.00	0.00	Diarrhea	0.00
K02064	0.00	0.00	0.00	0.00	Diarrhea	0.00
K01972	0.00	0.00	0.00	0.00	Health	0.00
K06200	0.00	0.00	0.00	0.00	Diarrhea	0.00
K11104	0.00	0.00	0.00	0.00	Diarrhea	0.00
K12268	0.00	0.00	0.00	0.00	Health	0.00
K03439	0.00	0.00	0.00	0.00	Health	0.00
K06179	0.00	0.00	0.00	0.00	Diarrhea	0.00
K03750	0.00	0.00	0.00	0.00	Diarrhea	0.00
K02509	0.00	0.00	0.00	0.00	Health	0.00
K03210	0.00	0.00	0.00	0.00	Diarrhea	0.00
K12269	0.00	0.00	0.00	0.00	Health	0.00
K00086	0.00	0.00	0.00	0.00	Health	0.00
K09762	0.00	0.00	0.00	0.00	Health	0.00
K01575	0.00	0.00	0.00	0.00	Health	0.00
K02669	0.00	0.00	0.00	0.00	Diarrhea	0.00
K03322	0.00	0.00	0.00	0.00	Health	0.00
K06518	0.00	0.00	0.00	0.00	Diarrhea	0.00
K10542	0.00	0.00	0.00	0.00	Diarrhea	0.00
K02895	0.00	0.00	0.00	0.00	Health	0.00
K02954	0.00	0.00	0.01	0.00	Health	0.00
K03753	0.00	0.00	0.00	0.00	Diarrhea	0.00
K00065	0.00	0.00	0.00	0.00	Diarrhea	0.00
K00995	0.00	0.00	0.00	0.00	Diarrhea	0.00
K03584	0.00	0.00	0.00	0.00	Health	0.00
K07704	0.00	0.00	0.00	0.00	Health	0.00

K12997	0.00	0.00	0.00	0.00	Health	0.00
K02434	0.00	0.00	0.00	0.00	Health	0.00
K03442	0.00	0.00	0.00	0.00	Diarrhea	0.00
K03709	0.00	0.00	0.00	0.00	Health	0.00
K02878	0.00	0.00	0.00	0.00	Health	0.00
K02887	0.00	0.00	0.00	0.00	Health	0.00
K01823	0.00	0.00	0.00	0.00	Health	0.00
K01889	0.00	0.00	0.00	0.00	Health	0.00
K02926	0.00	0.00	0.00	0.00	Health	0.00
K00615	0.00	0.00	0.00	0.00	Diarrhea	0.00
K01056	0.00	0.00	0.00	0.00	Health	0.00
K03312	0.00	0.00	0.00	0.00	Diarrhea	0.00
K03700	0.00	0.00	0.00	0.00	Health	0.00
K09155	0.00	0.00	0.00	0.00	Health	0.00
K02914	0.01	0.00	0.01	0.00	Health	0.00
K06949	0.00	0.00	0.00	0.00	Health	0.00
K00040	0.00	0.00	0.00	0.00	Diarrhea	0.00
K11066	0.00	0.00	0.00	0.00	Diarrhea	0.00
K02768	0.00	0.00	0.00	0.00	Diarrhea	0.00
K05916	0.00	0.00	0.00	0.00	Diarrhea	0.00
K20107	0.00	0.00	0.00	0.00	Health	0.00
K20108	0.00	0.00	0.00	0.00	Health	0.00
K19225	0.00	0.00	0.00	0.00	Health	0.00
K02745	0.00	0.00	0.00	0.00	Diarrhea	0.00
K07586	0.00	0.00	0.00	0.00	Health	0.00
K10254	0.00	0.00	0.00	0.00	Health	0.00
K00020	0.00	0.00	0.00	0.00	Health	0.00
K01419	0.00	0.00	0.00	0.00	Diarrhea	0.00
K01646	0.00	0.00	0.00	0.00	Diarrhea	0.00
K02876	0.00	0.00	0.00	0.00	Health	0.00
K14053	0.00	0.00	0.00	0.00	Diarrhea	0.00
K01012	0.00	0.00	0.00	0.00	Diarrhea	0.00
K01478	0.00	0.00	0.00	0.00	Health	0.00
K03705	0.00	0.00	0.00	0.00	Health	0.00
K07150	0.00	0.00	0.00	0.00	Diarrhea	0.00
K09815	0.00	0.00	0.00	0.00	Diarrhea	0.00
K16789	0.00	0.00	0.00	0.00	Health	0.00
K01046	0.00	0.00	0.00	0.00	Health	0.00
K02054	0.00	0.00	0.00	0.00	Diarrhea	0.00
K01095	0.00	0.00	0.00	0.00	Diarrhea	0.00
K01653	0.00	0.00	0.00	0.00	Diarrhea	0.00
K01939	0.00	0.00	0.00	0.00	Health	0.00
K00645	0.00	0.00	0.00	0.00	Diarrhea	0.00
K02822	0.00	0.00	0.00	0.00	Diarrhea	0.00

K03498	0.00	0.00	0.00	0.00	Diarrhea	0.00
K04043	0.00	0.00	0.00	0.00	Health	0.00
K06199	0.00	0.00	0.00	0.00	Health	0.00
K15553	0.00	0.00	0.00	0.00	Diarrhea	0.00
K06203	0.00	0.00	0.00	0.00	Diarrhea	0.00
K17810	0.00	0.00	0.00	0.00	Health	0.00
K01620	0.00	0.00	0.00	0.00	Diarrhea	0.00
K02982	0.00	0.00	0.00	0.00	Health	0.00
K03294	0.00	0.00	0.00	0.00	Health	0.00
K00077	0.00	0.00	0.00	0.00	Health	0.00
K00287	0.00	0.00	0.00	0.00	Diarrhea	0.00
K07277	0.00	0.00	0.00	0.00	Diarrhea	0.00
K03637	0.00	0.00	0.00	0.00	Diarrhea	0.00
K00845	0.00	0.00	0.00	0.00	Health	0.00
K01875	0.00	0.00	0.00	0.00	Health	0.00
K03609	0.00	0.00	0.00	0.00	Diarrhea	0.00
K00004	0.00	0.00	0.00	0.00	Health	0.00
K03713	0.00	0.00	0.00	0.00	Health	0.00
K09685	0.00	0.00	0.00	0.00	Health	0.00
K07776	0.00	0.00	0.00	0.00	Health	0.00
K00878	0.00	0.00	0.00	0.00	Health	0.00
K14742	0.00	0.00	0.00	0.00	Diarrhea	0.00
K02759	0.00	0.00	0.00	0.00	Diarrhea	0.00
K07705	0.00	0.00	0.00	0.00	Health	0.00
K18214	0.00	0.00	0.00	0.00	Health	0.00
K03697	0.00	0.00	0.00	0.00	Health	0.00
K03040	0.00	0.00	0.00	0.00	Health	0.00
K03517	0.00	0.00	0.00	0.00	Diarrhea	0.00
K00076	0.00	0.00	0.00	0.00	Diarrhea	0.00
K02035	0.00	0.00	0.00	0.00	Diarrhea	0.00
K03522	0.00	0.00	0.00	0.00	Diarrhea	0.00
K08093	0.00	0.00	0.00	0.00	Health	0.00
K22044	0.00	0.00	0.00	0.00	Health	0.00
K03707	0.00	0.00	0.00	0.00	Health	0.00
K06286	0.00	0.00	0.00	0.00	Health	0.00
K03046	0.00	0.00	0.00	0.00	Health	0.00
K08969	0.00	0.00	0.00	0.00	Health	0.00
K03449	0.00	0.00	0.00	0.00	Health	0.00
K02052	0.00	0.00	0.00	0.00	Diarrhea	0.00
K03386	0.00	0.00	0.00	0.00	Diarrhea	0.00
K07284	0.00	0.00	0.00	0.00	Health	0.00
K10824	0.00	0.00	0.00	0.00	Diarrhea	0.00
K01926	0.00	0.00	0.00	0.00	Health	0.00
K03561	0.00	0.00	0.00	0.00	Diarrhea	0.00

K07305	0.00	0.00	0.00	0.00	Health	0.00
K03072	0.00	0.00	0.00	0.00	Diarrhea	0.00
K10119	0.00	0.00	0.00	0.00	Health	0.00
K03698	0.00	0.00	0.00	0.00	Health	0.00
K02871	0.00	0.00	0.00	0.00	Health	0.00
K03499	0.00	0.00	0.00	0.00	Diarrhea	0.00
K11922	0.00	0.00	0.00	0.00	Diarrhea	0.00
K01677	0.00	0.00	0.00	0.00	Diarrhea	0.00
K16013	0.00	0.00	0.00	0.00	Health	0.00
K02314	0.00	0.00	0.00	0.00	Diarrhea	0.00
K21578	0.00	0.00	0.00	0.00	Health	0.00
K02233	0.00	0.00	0.00	0.00	Diarrhea	0.00
K02994	0.00	0.00	0.00	0.00	Health	0.00
K07139	0.00	0.00	0.00	0.00	Diarrhea	0.00
K18672	0.00	0.00	0.00	0.00	Diarrhea	0.00
K00969	0.00	0.00	0.00	0.00	Health	0.00
K03048	0.00	0.00	0.00	0.00	Health	0.00
K03704	0.00	0.00	0.00	0.00	Diarrhea	0.00
K03925	0.00	0.00	0.00	0.00	Health	0.00
K04773	0.00	0.00	0.00	0.00	Diarrhea	0.00
K00209	0.00	0.00	0.00	0.00	Health	0.00
K03546	0.00	0.00	0.00	0.00	Diarrhea	0.00
K00656	0.00	0.00	0.00	0.00	Diarrhea	0.00
K01588	0.00	0.00	0.00	0.00	Health	0.00
K01673	0.00	0.00	0.00	0.00	Diarrhea	0.00
K07584	0.00	0.00	0.00	0.00	Diarrhea	0.00
K16962	0.00	0.00	0.00	0.00	Health	0.00
K10026	0.00	0.00	0.00	0.00	Diarrhea	0.00
K06861	0.00	0.00	0.00	0.00	Diarrhea	0.00
K00674	0.00	0.00	0.00	0.00	Health	0.00
K01426	0.00	0.00	0.00	0.00	Diarrhea	0.00
K02907	0.00	0.00	0.00	0.00	Health	0.00
K10541	0.00	0.00	0.00	0.00	Diarrhea	0.00
K14088	0.00	0.00	0.00	0.00	Health	0.00
K02110	0.00	0.00	0.00	0.00	Diarrhea	0.00
K03530	0.00	0.00	0.00	0.00	Diarrhea	0.00
K01421	0.00	0.00	0.00	0.00	Health	0.00
K07533	0.00	0.00	0.00	0.00	Health	0.00
K03708	0.00	0.00	0.00	0.00	Diarrhea	0.00
K01953	0.00	0.00	0.00	0.00	Health	0.00
K15555	0.00	0.00	0.00	0.00	Diarrhea	0.00
K05979	0.00	0.00	0.00	0.00	Health	0.00
K06910	0.00	0.00	0.00	0.00	Diarrhea	0.00
K00784	0.00	0.00	0.00	0.00	Health	0.00

K11072	0.00	0.00	0.00	0.00	Health	0.00
K13292	0.00	0.00	0.00	0.00	Health	0.00
K01299	0.00	0.00	0.00	0.00	Diarrhea	0.00
K00927	0.00	0.00	0.00	0.00	Health	0.00
K02315	0.00	0.00	0.00	0.00	Diarrhea	0.00
K02881	0.00	0.00	0.00	0.00	Health	0.00
K07029	0.00	0.00	0.00	0.00	Health	0.00
K01567	0.00	0.00	0.00	0.00	Health	0.00
K02453	0.00	0.00	0.00	0.00	Diarrhea	0.00
K03696	0.00	0.00	0.00	0.00	Health	0.00
K03832	0.00	0.00	0.00	0.00	Diarrhea	0.00
K02650	0.00	0.00	0.00	0.00	Health	0.00
K00184	0.00	0.00	0.00	0.00	Health	0.00
K00761	0.00	0.00	0.00	0.00	Health	0.00
K00979	0.00	0.00	0.00	0.00	Diarrhea	0.00
K01577	0.00	0.00	0.00	0.00	Health	0.00
K01961	0.00	0.00	0.00	0.00	Diarrhea	0.00
K02443	0.00	0.00	0.00	0.00	Diarrhea	0.00
K06958	0.00	0.00	0.00	0.00	Health	0.00
K07107	0.00	0.00	0.00	0.00	Diarrhea	0.00
K07260	0.00	0.00	0.00	0.00	Health	0.00
K08301	0.00	0.00	0.00	0.00	Diarrhea	0.00
K22604	0.00	0.00	0.00	0.00	Health	0.00
K07357	0.00	0.00	0.00	0.00	Health	0.01
K01077	0.00	0.00	0.00	0.00	Diarrhea	0.01
K01935	0.00	0.00	0.00	0.00	Diarrhea	0.01
K06950	0.00	0.00	0.00	0.00	Health	0.01
K08365	0.00	0.00	0.00	0.00	Health	0.01
K00763	0.00	0.00	0.00	0.00	Health	0.01
K01998	0.00	0.00	0.00	0.00	Diarrhea	0.01
K02160	0.00	0.00	0.00	0.00	Diarrhea	0.01
K03687	0.00	0.00	0.00	0.00	Health	0.01
K16923	0.00	0.00	0.00	0.00	Health	0.01
K02456	0.00	0.00	0.00	0.00	Diarrhea	0.01
K02970	0.00	0.00	0.00	0.00	Health	0.01
K03559	0.00	0.00	0.00	0.00	Diarrhea	0.01
K03978	0.00	0.00	0.00	0.00	Health	0.01
K07741	0.00	0.00	0.00	0.00	Diarrhea	0.01
K08234	0.00	0.00	0.00	0.00	Diarrhea	0.01
K20116	0.00	0.00	0.00	0.00	Diarrhea	0.01
K20117	0.00	0.00	0.00	0.00	Diarrhea	0.01
K20118	0.00	0.00	0.00	0.00	Diarrhea	0.01
K00681	0.00	0.00	0.00	0.00	Diarrhea	0.01
K01749	0.00	0.00	0.00	0.00	Diarrhea	0.01

K02837	0.00	0.00	0.00	0.00	Health	0.01
K00980	0.00	0.00	0.00	0.00	Health	0.01
K01807	0.00	0.00	0.00	0.00	Health	0.01
K01869	0.00	0.00	0.00	0.00	Health	0.01
K13714	0.00	0.00	0.00	0.00	Health	0.01
K01788	0.00	0.00	0.00	0.00	Diarrhea	0.01
K03106	0.00	0.00	0.00	0.00	Diarrhea	0.01
K07571	0.00	0.00	0.00	0.00	Health	0.01
K11085	0.00	0.00	0.00	0.00	Diarrhea	0.01
K18138	0.00	0.00	0.00	0.00	Diarrhea	0.01
K20342	0.00	0.00	0.00	0.00	Health	0.01
K07149	0.00	0.00	0.00	0.00	Diarrhea	0.01
K02769	0.00	0.00	0.00	0.00	Diarrhea	0.01
K01338	0.00	0.00	0.00	0.00	Diarrhea	0.01
K02243	0.00	0.00	0.00	0.00	Health	0.01
K00748	0.00	0.00	0.00	0.00	Diarrhea	0.01
K01356	0.00	0.00	0.00	0.00	Health	0.01
K01761	0.00	0.00	0.00	0.00	Diarrhea	0.01
K01876	0.00	0.00	0.00	0.00	Health	0.01
K02313	0.00	0.00	0.00	0.00	Health	0.01
K05825	0.00	0.00	0.00	0.00	Health	0.01
K09775	0.00	0.00	0.00	0.00	Diarrhea	0.01
K09778	0.00	0.00	0.00	0.00	Diarrhea	0.01
K07032	0.00	0.00	0.00	0.00	Health	0.01
K01911	0.00	0.00	0.00	0.00	Health	0.01
K07315	0.00	0.00	0.00	0.00	Diarrhea	0.01
K04072	0.00	0.00	0.00	0.00	Diarrhea	0.01
K01589	0.00	0.00	0.00	0.00	Health	0.01
K03070	0.00	0.00	0.00	0.00	Health	0.01
K03775	0.00	0.00	0.00	0.00	Diarrhea	0.01
K06178	0.00	0.00	0.00	0.00	Diarrhea	0.01
K06925	0.00	0.00	0.00	0.00	Diarrhea	0.01
K00100	0.00	0.00	0.00	0.00	Diarrhea	0.01
K03555	0.00	0.00	0.00	0.00	Health	0.01
K02804	0.00	0.00	0.00	0.00	Diarrhea	0.01
K04023	0.00	0.00	0.00	0.00	Diarrhea	0.01
K02057	0.00	0.00	0.00	0.00	Diarrhea	0.01
K03616	0.00	0.00	0.00	0.00	Diarrhea	0.01
K03652	0.00	0.00	0.00	0.00	Health	0.01
K05595	0.00	0.00	0.00	0.00	Diarrhea	0.01
K09858	0.00	0.00	0.00	0.00	Diarrhea	0.01
K08981	0.00	0.00	0.00	0.00	Health	0.01
K00899	0.00	0.00	0.00	0.00	Diarrhea	0.01
K02939	0.00	0.00	0.00	0.00	Health	0.01

K03635	0.00	0.00	0.00	0.00	Health	0.01
K03695	0.00	0.00	0.00	0.00	Diarrhea	0.01
K09790	0.00	0.00	0.00	0.00	Diarrhea	0.01
K21977	0.00	0.00	0.00	0.00	Health	0.01
K07007	0.00	0.00	0.00	0.00	Health	0.01
K03706	0.00	0.00	0.00	0.00	Health	0.01
K02111	0.00	0.00	0.00	0.00	Health	0.01
K02647	0.00	0.00	0.00	0.00	Diarrhea	0.01
K02967	0.00	0.00	0.00	0.00	Health	0.01
K03310	0.00	0.00	0.00	0.00	Diarrhea	0.01
K11733	0.00	0.00	0.00	0.00	Health	0.01
K01232	0.00	0.00	0.00	0.00	Diarrhea	0.01
K13280	0.00	0.00	0.00	0.00	Health	0.01
K00869	0.00	0.00	0.00	0.00	Health	0.01
K01198	0.00	0.00	0.00	0.00	Health	0.01
K13281	0.00	0.00	0.00	0.00	Health	0.01
K02304	0.00	0.00	0.00	0.00	Diarrhea	0.01
K08153	0.00	0.00	0.00	0.00	Diarrhea	0.01
K15383	0.00	0.00	0.00	0.00	Diarrhea	0.01

Supplementary Table 2: The relative abundance of differential KOs in H-FMT and D-FMT group

KO	Mean (D-FMT group)	SD (D-FMT group)	Mean (H-FMT group)	SD (H-FMT group)	Enrichment	P_adjust
K00012	0.00	0.00	0.00	0.00	D-FMT	0.00
K00024	0.00	0.00	0.00	0.00	D-FMT	0.00
K00031	0.00	0.00	0.00	0.00	D-FMT	0.00
K00045	0.00	0.00	0.00	0.00	H-FMT	0.00
K00067	0.00	0.00	0.00	0.00	H-FMT	0.00
K00075	0.00	0.00	0.00	0.00	H-FMT	0.00
K00176	0.00	0.00	0.00	0.00	D-FMT	0.00
K00179	0.00	0.00	0.00	0.00	D-FMT	0.00
K00210	0.00	0.00	0.00	0.00	D-FMT	0.00
K00240	0.00	0.00	0.00	0.00	D-FMT	0.00
K00286	0.00	0.00	0.00	0.00	D-FMT	0.00
K00297	0.00	0.00	0.00	0.00	D-FMT	0.00
K00341	0.00	0.00	0.00	0.00	D-FMT	0.00
K00342	0.00	0.00	0.00	0.00	D-FMT	0.00
K00343	0.00	0.00	0.00	0.00	D-FMT	0.00
K00346	0.00	0.00	0.00	0.00	D-FMT	0.00
K00349	0.00	0.00	0.00	0.00	D-FMT	0.00
K00351	0.00	0.00	0.00	0.00	D-FMT	0.00
K00525	0.00	0.00	0.00	0.00	D-FMT	0.00
K00602	0.00	0.00	0.00	0.00	D-FMT	0.00

K00625	0.00	0.00	0.00	0.00	D-FMT	0.00
K00648	0.00	0.00	0.00	0.00	H-FMT	0.00
K00657	0.00	0.00	0.00	0.00	D-FMT	0.00
K00677	0.00	0.00	0.00	0.00	D-FMT	0.00
K00684	0.00	0.00	0.00	0.00	D-FMT	0.00
K00721	0.00	0.00	0.00	0.00	D-FMT	0.00
K00761	0.00	0.00	0.00	0.00	D-FMT	0.00
K00768	0.00	0.00	0.00	0.00	D-FMT	0.00
K00783	0.00	0.00	0.00	0.00	H-FMT	0.00
K00791	0.00	0.00	0.00	0.00	H-FMT	0.00
K00793	0.00	0.00	0.00	0.00	H-FMT	0.00
K00794	0.00	0.00	0.00	0.00	D-FMT	0.00
K00798	0.00	0.00	0.00	0.00	D-FMT	0.00
K00812	0.00	0.00	0.00	0.00	D-FMT	0.00
K00819	0.00	0.00	0.00	0.00	D-FMT	0.00
K00821	0.00	0.00	0.00	0.00	D-FMT	0.00
K00826	0.00	0.00	0.00	0.00	D-FMT	0.00
K00833	0.00	0.00	0.00	0.00	H-FMT	0.00
K00845	0.00	0.00	0.00	0.00	H-FMT	0.00
K00859	0.00	0.00	0.00	0.00	H-FMT	0.00
K00912	0.00	0.00	0.00	0.00	H-FMT	0.00
K00931	0.00	0.00	0.00	0.00	H-FMT	0.00
K00940	0.00	0.00	0.00	0.00	H-FMT	0.00
K00942	0.00	0.00	0.00	0.00	D-FMT	0.00
K00945	0.00	0.00	0.00	0.00	H-FMT	0.00
K00946	0.00	0.00	0.00	0.00	D-FMT	0.00
K00949	0.00	0.00	0.00	0.00	H-FMT	0.00
K00951	0.00	0.00	0.00	0.00	D-FMT	0.00
K00962	0.00	0.00	0.00	0.00	D-FMT	0.00
K00979	0.00	0.00	0.00	0.00	D-FMT	0.00
K00981	0.00	0.00	0.00	0.00	H-FMT	0.00
K01051	0.00	0.00	0.00	0.00	D-FMT	0.00
K01056	0.00	0.00	0.00	0.00	H-FMT	0.00
K01057	0.00	0.00	0.00	0.00	H-FMT	0.00
K01077	0.00	0.00	0.00	0.00	D-FMT	0.00
K01079	0.00	0.00	0.00	0.00	D-FMT	0.00
K01126	0.00	0.00	0.00	0.00	H-FMT	0.00
K01142	0.00	0.00	0.00	0.00	D-FMT	0.00
K01153	0.00	0.00	0.00	0.00	H-FMT	0.00
K01159	0.00	0.00	0.00	0.00	D-FMT	0.00
K01185	0.00	0.00	0.00	0.00	D-FMT	0.00
K01192	0.00	0.00	0.00	0.00	H-FMT	0.00
K01193	0.00	0.00	0.00	0.00	H-FMT	0.00
K01235	0.00	0.00	0.00	0.00	H-FMT	0.00

K01277	0.00	0.00	0.00	0.00	H-FMT	0.00
K01278	0.00	0.00	0.00	0.00	H-FMT	0.00
K01284	0.00	0.00	0.00	0.00	D-FMT	0.00
K01297	0.00	0.00	0.00	0.00	H-FMT	0.00
K01358	0.00	0.00	0.00	0.00	D-FMT	0.00
K01372	0.00	0.00	0.00	0.00	H-FMT	0.00
K01425	0.00	0.00	0.00	0.00	H-FMT	0.00
K01433	0.00	0.00	0.00	0.00	D-FMT	0.00
K01438	0.00	0.00	0.00	0.00	D-FMT	0.00
K01443	0.00	0.00	0.00	0.00	D-FMT	0.00
K01468	0.00	0.00	0.00	0.00	H-FMT	0.00
K01470	0.00	0.00	0.00	0.00	D-FMT	0.00
K01495	0.00	0.00	0.00	0.00	D-FMT	0.00
K01524	0.00	0.00	0.00	0.00	D-FMT	0.00
K01547	0.00	0.00	0.00	0.00	D-FMT	0.00
K01572	0.00	0.00	0.00	0.00	H-FMT	0.00
K01573	0.00	0.00	0.00	0.00	D-FMT	0.00
K01586	0.00	0.00	0.00	0.00	H-FMT	0.00
K01607	0.00	0.00	0.00	0.00	H-FMT	0.00
K01619	0.00	0.00	0.00	0.00	D-FMT	0.00
K01620	0.00	0.00	0.00	0.00	D-FMT	0.00
K01657	0.00	0.00	0.00	0.00	D-FMT	0.00
K01686	0.00	0.00	0.00	0.00	D-FMT	0.00
K01695	0.00	0.00	0.00	0.00	H-FMT	0.00
K01696	0.00	0.00	0.00	0.00	D-FMT	0.00
K01710	0.00	0.00	0.00	0.00	D-FMT	0.00
K01711	0.00	0.00	0.00	0.00	D-FMT	0.00
K01719	0.00	0.00	0.00	0.00	D-FMT	0.00
K01734	0.00	0.00	0.00	0.00	D-FMT	0.00
K01736	0.00	0.00	0.00	0.00	H-FMT	0.00
K01738	0.00	0.00	0.00	0.00	H-FMT	0.00
K01754	0.00	0.00	0.00	0.00	D-FMT	0.00
K01755	0.00	0.00	0.00	0.00	D-FMT	0.00
K01759	0.00	0.00	0.00	0.00	D-FMT	0.00
K01785	0.00	0.00	0.00	0.00	H-FMT	0.00
K01807	0.00	0.00	0.00	0.00	H-FMT	0.00
K01809	0.00	0.00	0.00	0.00	D-FMT	0.00
K01817	0.00	0.00	0.00	0.00	H-FMT	0.00
K01866	0.00	0.00	0.00	0.00	D-FMT	0.00
K01872	0.00	0.00	0.00	0.00	D-FMT	0.00
K01873	0.00	0.00	0.00	0.00	H-FMT	0.00
K01878	0.00	0.00	0.00	0.00	D-FMT	0.00
K01880	0.00	0.00	0.00	0.00	D-FMT	0.00
K01914	0.00	0.00	0.00	0.00	D-FMT	0.00

K01915	0.00	0.00	0.00	0.00	D-FMT	0.00
K01921	0.00	0.00	0.00	0.00	H-FMT	0.00
K01928	0.00	0.00	0.00	0.00	H-FMT	0.00
K01935	0.00	0.00	0.00	0.00	D-FMT	0.00
K01950	0.00	0.00	0.00	0.00	H-FMT	0.00
K01951	0.00	0.00	0.00	0.00	H-FMT	0.00
K01990	0.00	0.00	0.00	0.00	H-FMT	0.00
K02001	0.00	0.00	0.00	0.00	D-FMT	0.00
K02013	0.00	0.00	0.00	0.00	H-FMT	0.00
K02015	0.00	0.00	0.00	0.00	H-FMT	0.00
K02065	0.00	0.00	0.00	0.00	H-FMT	0.00
K02069	0.00	0.00	0.00	0.00	D-FMT	0.00
K02078	0.00	0.00	0.00	0.00	D-FMT	0.00
K02081	0.00	0.00	0.00	0.00	H-FMT	0.00
K02114	0.00	0.00	0.00	0.00	D-FMT	0.00
K02123	0.00	0.00	0.00	0.00	H-FMT	0.00
K02224	0.00	0.00	0.00	0.00	D-FMT	0.00
K02226	0.00	0.00	0.00	0.00	D-FMT	0.00
K02231	0.00	0.00	0.00	0.00	D-FMT	0.00
K02335	0.00	0.00	0.00	0.00	D-FMT	0.00
K02337	0.00	0.00	0.00	0.00	D-FMT	0.00
K02341	0.00	0.00	0.00	0.00	D-FMT	0.00
K02342	0.00	0.00	0.00	0.00	D-FMT	0.00
K02428	0.00	0.00	0.00	0.00	D-FMT	0.00
K02429	0.00	0.00	0.00	0.00	D-FMT	0.00
K02472	0.00	0.00	0.00	0.00	D-FMT	0.00
K02500	0.00	0.00	0.00	0.00	H-FMT	0.00
K02517	0.00	0.00	0.00	0.00	D-FMT	0.00
K02551	0.00	0.00	0.00	0.00	H-FMT	0.00
K02563	0.00	0.00	0.00	0.00	H-FMT	0.00
K02614	0.00	0.00	0.00	0.00	D-FMT	0.00
K02619	0.00	0.00	0.00	0.00	D-FMT	0.00
K02621	0.00	0.00	0.00	0.00	D-FMT	0.00
K02622	0.00	0.00	0.00	0.00	D-FMT	0.00
K02687	0.00	0.00	0.00	0.00	D-FMT	0.00
K02775	0.00	0.00	0.00	0.00	D-FMT	0.00
K02834	0.00	0.00	0.00	0.00	D-FMT	0.00
K02839	0.00	0.00	0.00	0.00	H-FMT	0.00
K02871	0.00	0.00	0.00	0.00	D-FMT	0.00
K02892	0.00	0.00	0.00	0.00	D-FMT	0.00
K02913	0.01	0.00	0.01	0.00	D-FMT	0.00
K02926	0.00	0.00	0.00	0.00	D-FMT	0.00
K02933	0.00	0.00	0.00	0.00	D-FMT	0.00
K02959	0.00	0.00	0.00	0.00	D-FMT	0.00

K02965	0.01	0.00	0.00	0.00	D-FMT	0.00
K02986	0.00	0.00	0.00	0.00	D-FMT	0.00
K02992	0.00	0.00	0.00	0.00	D-FMT	0.00
K03070	0.00	0.00	0.00	0.00	H-FMT	0.00
K03076	0.00	0.00	0.00	0.00	D-FMT	0.00
K03092	0.00	0.00	0.00	0.00	D-FMT	0.00
K03150	0.00	0.00	0.00	0.00	H-FMT	0.00
K03152	0.00	0.00	0.00	0.00	D-FMT	0.00
K03210	0.00	0.00	0.00	0.00	D-FMT	0.00
K03215	0.00	0.00	0.00	0.00	H-FMT	0.00
K03217	0.00	0.00	0.00	0.00	H-FMT	0.00
K03303	0.00	0.00	0.00	0.00	D-FMT	0.00
K03305	0.00	0.00	0.00	0.00	H-FMT	0.00
K03312	0.00	0.00	0.00	0.00	D-FMT	0.00
K03385	0.00	0.00	0.00	0.00	H-FMT	0.00
K03424	0.00	0.00	0.00	0.00	H-FMT	0.00
K03430	0.00	0.00	0.00	0.00	D-FMT	0.00
K03439	0.00	0.00	0.00	0.00	H-FMT	0.00
K03442	0.00	0.00	0.00	0.00	D-FMT	0.00
K03455	0.00	0.00	0.00	0.00	D-FMT	0.00
K03470	0.00	0.00	0.00	0.00	D-FMT	0.00
K03526	0.00	0.00	0.00	0.00	H-FMT	0.00
K03544	0.00	0.00	0.00	0.00	D-FMT	0.00
K03550	0.00	0.00	0.00	0.00	H-FMT	0.00
K03558	0.00	0.00	0.00	0.00	H-FMT	0.00
K03559	0.00	0.00	0.00	0.00	D-FMT	0.00
K03565	0.00	0.00	0.00	0.00	D-FMT	0.00
K03585	0.00	0.00	0.00	0.00	D-FMT	0.00
K03588	0.00	0.00	0.00	0.00	D-FMT	0.00
K03601	0.00	0.00	0.00	0.00	D-FMT	0.00
K03602	0.00	0.00	0.00	0.00	D-FMT	0.00
K03606	0.00	0.00	0.00	0.00	D-FMT	0.00
K03614	0.00	0.00	0.00	0.00	D-FMT	0.00
K03650	0.00	0.00	0.00	0.00	H-FMT	0.00
K03655	0.00	0.00	0.00	0.00	D-FMT	0.00
K03686	0.00	0.00	0.00	0.00	H-FMT	0.00
K03694	0.00	0.00	0.00	0.00	D-FMT	0.00
K03695	0.00	0.00	0.00	0.00	D-FMT	0.00
K03703	0.00	0.00	0.00	0.00	H-FMT	0.00
K03711	0.00	0.00	0.00	0.00	D-FMT	0.00
K03742	0.00	0.00	0.00	0.00	H-FMT	0.00
K03743	0.00	0.00	0.00	0.00	D-FMT	0.00
K03771	0.00	0.00	0.00	0.00	D-FMT	0.00
K03773	0.00	0.00	0.00	0.00	D-FMT	0.00

K03798	0.00	0.00	0.00	0.00	H-FMT	0.00
K03811	0.00	0.00	0.00	0.00	D-FMT	0.00
K03827	0.00	0.00	0.00	0.00	D-FMT	0.00
K03931	0.00	0.00	0.00	0.00	D-FMT	0.00
K03975	0.00	0.00	0.00	0.00	D-FMT	0.00
K04041	0.00	0.00	0.00	0.00	H-FMT	0.00
K04075	0.00	0.00	0.00	0.00	H-FMT	0.00
K04477	0.00	0.00	0.00	0.00	D-FMT	0.00
K04567	0.00	0.00	0.00	0.00	H-FMT	0.00
K05515	0.00	0.00	0.00	0.00	D-FMT	0.00
K05592	0.00	0.00	0.00	0.00	D-FMT	0.00
K05601	0.00	0.00	0.00	0.00	H-FMT	0.00
K05770	0.00	0.00	0.00	0.00	D-FMT	0.00
K05801	0.00	0.00	0.00	0.00	D-FMT	0.00
K05989	0.00	0.00	0.00	0.00	H-FMT	0.00
K06041	0.00	0.00	0.00	0.00	D-FMT	0.00
K06076	0.00	0.00	0.00	0.00	D-FMT	0.00
K06153	0.00	0.00	0.00	0.00	D-FMT	0.00
K06201	0.00	0.00	0.00	0.00	H-FMT	0.00
K06217	0.00	0.00	0.00	0.00	D-FMT	0.00
K06871	0.00	0.00	0.00	0.00	D-FMT	0.00
K06872	0.00	0.00	0.00	0.00	D-FMT	0.00
K06897	0.00	0.00	0.00	0.00	H-FMT	0.00
K06919	0.00	0.00	0.00	0.00	D-FMT	0.00
K06941	0.00	0.00	0.00	0.00	H-FMT	0.00
K06969	0.00	0.00	0.00	0.00	H-FMT	0.00
K06973	0.00	0.00	0.00	0.00	D-FMT	0.00
K06978	0.00	0.00	0.00	0.00	D-FMT	0.00
K07025	0.00	0.00	0.00	0.00	H-FMT	0.00
K07031	0.00	0.00	0.00	0.00	D-FMT	0.00
K07037	0.00	0.00	0.00	0.00	D-FMT	0.00
K07078	0.00	0.00	0.00	0.00	H-FMT	0.00
K07106	0.00	0.00	0.00	0.00	D-FMT	0.00
K07107	0.00	0.00	0.00	0.00	D-FMT	0.00
K07126	0.00	0.00	0.00	0.00	H-FMT	0.00
K07139	0.00	0.00	0.00	0.00	D-FMT	0.00
K07154	0.00	0.00	0.00	0.00	H-FMT	0.00
K07173	0.00	0.00	0.00	0.00	D-FMT	0.00
K07192	0.00	0.00	0.00	0.00	D-FMT	0.00
K07221	0.00	0.00	0.00	0.00	D-FMT	0.00
K07263	0.00	0.00	0.00	0.00	D-FMT	0.00
K07271	0.00	0.00	0.00	0.00	H-FMT	0.00
K07322	0.00	0.00	0.00	0.00	D-FMT	0.00
K07391	0.00	0.00	0.00	0.00	H-FMT	0.00

K07444	0.00	0.00	0.00	0.00	H-FMT	0.00
K07447	0.00	0.00	0.00	0.00	H-FMT	0.00
K07460	0.00	0.00	0.00	0.00	D-FMT	0.00
K07566	0.00	0.00	0.00	0.00	D-FMT	0.00
K07568	0.00	0.00	0.00	0.00	D-FMT	0.00
K07588	0.00	0.00	0.00	0.00	D-FMT	0.00
K07713	0.00	0.00	0.00	0.00	D-FMT	0.00
K07783	0.00	0.00	0.00	0.00	H-FMT	0.00
K08222	0.00	0.00	0.00	0.00	H-FMT	0.00
K08223	0.00	0.00	0.00	0.00	H-FMT	0.00
K08641	0.00	0.00	0.00	0.00	H-FMT	0.00
K09014	0.00	0.00	0.00	0.00	D-FMT	0.00
K09457	0.00	0.00	0.00	0.00	D-FMT	0.00
K09458	0.00	0.00	0.00	0.00	D-FMT	0.00
K09474	0.00	0.00	0.00	0.00	D-FMT	0.00
K09690	0.00	0.00	0.00	0.00	D-FMT	0.00
K09704	0.00	0.00	0.00	0.00	H-FMT	0.00
K09710	0.00	0.00	0.00	0.00	D-FMT	0.00
K09797	0.00	0.00	0.00	0.00	D-FMT	0.00
K10206	0.00	0.00	0.00	0.00	D-FMT	0.00
K11068	0.00	0.00	0.00	0.00	D-FMT	0.00
K11105	0.00	0.00	0.00	0.00	D-FMT	0.00
K11717	0.00	0.00	0.00	0.00	D-FMT	0.00
K11720	0.00	0.00	0.00	0.00	H-FMT	0.00
K11749	0.00	0.00	0.00	0.00	D-FMT	0.00
K11753	0.00	0.00	0.00	0.00	D-FMT	0.00
K11991	0.00	0.00	0.00	0.00	D-FMT	0.00
K12267	0.00	0.00	0.00	0.00	D-FMT	0.00
K12524	0.00	0.00	0.00	0.00	D-FMT	0.00
K12573	0.00	0.00	0.00	0.00	D-FMT	0.00
K13051	0.00	0.00	0.00	0.00	D-FMT	0.00
K13694	0.00	0.00	0.00	0.00	D-FMT	0.00
K14415	0.00	0.00	0.00	0.00	H-FMT	0.00
K14445	0.00	0.00	0.00	0.00	H-FMT	0.00
K15583	0.00	0.00	0.00	0.00	D-FMT	0.00
K15726	0.00	0.00	0.00	0.00	H-FMT	0.00
K15923	0.00	0.00	0.00	0.00	D-FMT	0.00
K16053	0.00	0.00	0.00	0.00	D-FMT	0.00
K17103	0.00	0.00	0.00	0.00	D-FMT	0.00
K19052	0.00	0.00	0.00	0.00	H-FMT	0.00
K20866	0.00	0.00	0.00	0.00	H-FMT	0.00
K20885	0.00	0.00	0.00	0.00	H-FMT	0.00
K21575	0.00	0.00	0.00	0.00	D-FMT	0.00
K23003	0.00	0.00	0.00	0.00	H-FMT	0.00

K23004	0.00	0.00	0.00	0.00	H-FMT	0.00
K00041	0.00	0.00	0.00	0.00	H-FMT	0.00
K00058	0.00	0.00	0.00	0.00	H-FMT	0.00
K00099	0.00	0.00	0.00	0.00	H-FMT	0.00
K00331	0.00	0.00	0.00	0.00	D-FMT	0.00
K00561	0.00	0.00	0.00	0.00	D-FMT	0.00
K00658	0.00	0.00	0.00	0.00	H-FMT	0.00
K00700	0.00	0.00	0.00	0.00	H-FMT	0.00
K00762	0.00	0.00	0.00	0.00	D-FMT	0.00
K00995	0.00	0.00	0.00	0.00	H-FMT	0.00
K01258	0.00	0.00	0.00	0.00	H-FMT	0.00
K01462	0.00	0.00	0.00	0.00	H-FMT	0.00
K01491	0.00	0.00	0.00	0.00	D-FMT	0.00
K01613	0.00	0.00	0.00	0.00	H-FMT	0.00
K01629	0.00	0.00	0.00	0.00	H-FMT	0.00
K01647	0.00	0.00	0.00	0.00	D-FMT	0.00
K01662	0.00	0.00	0.00	0.00	D-FMT	0.00
K01744	0.00	0.00	0.00	0.00	H-FMT	0.00
K01813	0.00	0.00	0.00	0.00	H-FMT	0.00
K01883	0.00	0.00	0.00	0.00	H-FMT	0.00
K01889	0.00	0.00	0.00	0.00	H-FMT	0.00
K01897	0.00	0.00	0.00	0.00	H-FMT	0.00
K01925	0.00	0.00	0.00	0.00	H-FMT	0.00
K01972	0.00	0.00	0.00	0.00	H-FMT	0.00
K02038	0.00	0.00	0.00	0.00	H-FMT	0.00
K02343	0.00	0.00	0.00	0.00	H-FMT	0.00
K02501	0.00	0.00	0.00	0.00	H-FMT	0.00
K02503	0.00	0.00	0.00	0.00	D-FMT	0.00
K02528	0.00	0.00	0.00	0.00	D-FMT	0.00
K02888	0.00	0.00	0.00	0.00	D-FMT	0.00
K03073	0.00	0.00	0.00	0.00	D-FMT	0.00
K03179	0.00	0.00	0.00	0.00	D-FMT	0.00
K03218	0.00	0.00	0.00	0.00	D-FMT	0.00
K03270	0.00	0.00	0.00	0.00	H-FMT	0.00
K03273	0.00	0.00	0.00	0.00	D-FMT	0.00
K03465	0.00	0.00	0.00	0.00	D-FMT	0.00
K03564	0.00	0.00	0.00	0.00	D-FMT	0.00
K03572	0.00	0.00	0.00	0.00	H-FMT	0.00
K03615	0.00	0.00	0.00	0.00	H-FMT	0.00
K03625	0.00	0.00	0.00	0.00	D-FMT	0.00
K03641	0.00	0.00	0.00	0.00	D-FMT	0.00
K03685	0.00	0.00	0.00	0.00	H-FMT	0.00
K03701	0.00	0.00	0.00	0.00	H-FMT	0.00
K03770	0.00	0.00	0.00	0.00	D-FMT	0.00

K03781	0.00	0.00	0.00	0.00	D-FMT	0.00
K03797	0.00	0.00	0.00	0.00	H-FMT	0.00
K03800	0.00	0.00	0.00	0.00	H-FMT	0.00
K03832	0.00	0.00	0.00	0.00	H-FMT	0.00
K03979	0.00	0.00	0.00	0.00	H-FMT	0.00
K04069	0.00	0.00	0.00	0.00	D-FMT	0.00
K05837	0.00	0.00	0.00	0.00	H-FMT	0.00
K06920	0.00	0.00	0.00	0.00	H-FMT	0.00
K07079	0.00	0.00	0.00	0.00	D-FMT	0.00
K07277	0.00	0.00	0.00	0.00	D-FMT	0.00
K07456	0.00	0.00	0.00	0.00	D-FMT	0.00
K07467	0.00	0.00	0.00	0.00	D-FMT	0.00
K07560	0.00	0.00	0.00	0.00	H-FMT	0.00
K08961	0.00	0.00	0.00	0.00	H-FMT	0.00
K09748	0.00	0.00	0.00	0.00	D-FMT	0.00
K09761	0.00	0.00	0.00	0.00	D-FMT	0.00
K09789	0.00	0.00	0.00	0.00	H-FMT	0.00
K09790	0.00	0.00	0.00	0.00	H-FMT	0.00
K11072	0.00	0.00	0.00	0.00	H-FMT	0.00
K18332	0.00	0.00	0.00	0.00	H-FMT	0.00
K18707	0.00	0.00	0.00	0.00	H-FMT	0.00
K18928	0.00	0.00	0.00	0.00	H-FMT	0.00
K19802	0.00	0.00	0.00	0.00	H-FMT	0.00
K00014	0.00	0.00	0.00	0.00	D-FMT	0.00
K00338	0.00	0.00	0.00	0.00	H-FMT	0.00
K00554	0.00	0.00	0.00	0.00	D-FMT	0.00
K00765	0.00	0.00	0.00	0.00	H-FMT	0.00
K00788	0.00	0.00	0.00	0.00	H-FMT	0.00
K00891	0.00	0.00	0.00	0.00	H-FMT	0.00
K00930	0.00	0.00	0.00	0.00	H-FMT	0.00
K00956	0.00	0.00	0.00	0.00	H-FMT	0.00
K01154	0.00	0.00	0.00	0.00	D-FMT	0.00
K01156	0.00	0.00	0.00	0.00	D-FMT	0.00
K01186	0.00	0.00	0.00	0.00	H-FMT	0.00
K01198	0.00	0.00	0.00	0.00	H-FMT	0.00
K01251	0.00	0.00	0.00	0.00	H-FMT	0.00
K01262	0.00	0.00	0.00	0.00	H-FMT	0.00
K01409	0.00	0.00	0.00	0.00	D-FMT	0.00
K01652	0.00	0.00	0.00	0.00	H-FMT	0.00
K01770	0.00	0.00	0.00	0.00	H-FMT	0.00
K01804	0.00	0.00	0.00	0.00	H-FMT	0.00
K01810	0.00	0.00	0.00	0.00	D-FMT	0.00
K01814	0.00	0.00	0.00	0.00	H-FMT	0.00
K01918	0.00	0.00	0.00	0.00	H-FMT	0.00

K01938	0.00	0.00	0.00	0.00	H-FMT	0.00
K02036	0.00	0.00	0.00	0.00	H-FMT	0.00
K02217	0.00	0.00	0.00	0.00	H-FMT	0.00
K02355	0.00	0.00	0.00	0.00	D-FMT	0.00
K02358	0.00	0.00	0.00	0.00	D-FMT	0.00
K02519	0.00	0.00	0.00	0.00	H-FMT	0.00
K02548	0.00	0.00	0.00	0.00	H-FMT	0.00
K02916	0.00	0.00	0.00	0.00	D-FMT	0.00
K02990	0.00	0.00	0.00	0.00	D-FMT	0.00
K03100	0.00	0.00	0.00	0.00	D-FMT	0.00
K03438	0.00	0.00	0.00	0.00	H-FMT	0.00
K03501	0.00	0.00	0.00	0.00	H-FMT	0.00
K03644	0.00	0.00	0.00	0.00	H-FMT	0.00
K03768	0.00	0.00	0.00	0.00	D-FMT	0.00
K04068	0.00	0.00	0.00	0.00	H-FMT	0.00
K04720	0.00	0.00	0.00	0.00	D-FMT	0.00
K06187	0.00	0.00	0.00	0.00	H-FMT	0.00
K06970	0.00	0.00	0.00	0.00	H-FMT	0.00
K09810	0.00	0.00	0.00	0.00	H-FMT	0.00
K09825	0.00	0.00	0.00	0.00	D-FMT	0.00
K12942	0.00	0.00	0.00	0.00	D-FMT	0.00
K13993	0.00	0.00	0.00	0.00	D-FMT	0.00
K15876	0.00	0.00	0.00	0.00	H-FMT	0.00
K18929	0.00	0.00	0.00	0.00	H-FMT	0.00
K21055	0.00	0.00	0.00	0.00	H-FMT	0.00
K21279	0.00	0.00	0.00	0.00	H-FMT	0.00
K00116	0.00	0.00	0.00	0.00	D-FMT	0.00
K00208	0.00	0.00	0.00	0.00	D-FMT	0.00
K00246	0.00	0.00	0.00	0.00	D-FMT	0.00
K00405	0.00	0.00	0.00	0.00	D-FMT	0.00
K00406	0.00	0.00	0.00	0.00	D-FMT	0.00
K00412	0.00	0.00	0.00	0.00	D-FMT	0.00
K00428	0.00	0.00	0.00	0.00	D-FMT	0.00
K00459	0.00	0.00	0.00	0.00	D-FMT	0.00
K00557	0.00	0.00	0.00	0.00	D-FMT	0.00
K01174	0.00	0.00	0.00	0.00	D-FMT	0.00
K01682	0.00	0.00	0.00	0.00	D-FMT	0.00
K01739	0.00	0.00	0.00	0.00	D-FMT	0.00
K01772	0.00	0.00	0.00	0.00	D-FMT	0.00
K01779	0.00	0.00	0.00	0.00	D-FMT	0.00
K02393	0.00	0.00	0.00	0.00	D-FMT	0.00
K02394	0.00	0.00	0.00	0.00	D-FMT	0.00
K02397	0.00	0.00	0.00	0.00	D-FMT	0.00
K02404	0.00	0.00	0.00	0.00	D-FMT	0.00

K02407	0.00	0.00	0.00	0.00	D-FMT	0.00
K02409	0.00	0.00	0.00	0.00	D-FMT	0.00
K02417	0.00	0.00	0.00	0.00	D-FMT	0.00
K02422	0.00	0.00	0.00	0.00	D-FMT	0.00
K02454	0.00	0.00	0.00	0.00	D-FMT	0.00
K02484	0.00	0.00	0.00	0.00	D-FMT	0.00
K02556	0.00	0.00	0.00	0.00	D-FMT	0.00
K02567	0.00	0.00	0.00	0.00	D-FMT	0.00
K02574	0.00	0.00	0.00	0.00	D-FMT	0.00
K03272	0.00	0.00	0.00	0.00	D-FMT	0.00
K03407	0.00	0.00	0.00	0.00	D-FMT	0.00
K03412	0.00	0.00	0.00	0.00	D-FMT	0.00
K03415	0.00	0.00	0.00	0.00	D-FMT	0.00
K03417	0.00	0.00	0.00	0.00	D-FMT	0.00
K03582	0.00	0.00	0.00	0.00	D-FMT	0.00
K03593	0.00	0.00	0.00	0.00	D-FMT	0.00
K03611	0.00	0.00	0.00	0.00	D-FMT	0.00
K03667	0.00	0.00	0.00	0.00	D-FMT	0.00
K03707	0.00	0.00	0.00	0.00	D-FMT	0.00
K03760	0.00	0.00	0.00	0.00	D-FMT	0.00
K03761	0.00	0.00	0.00	0.00	D-FMT	0.00
K03830	0.00	0.00	0.00	0.00	D-FMT	0.00
K03837	0.00	0.00	0.00	0.00	D-FMT	0.00
K03893	0.00	0.00	0.00	0.00	D-FMT	0.00
K04562	0.00	0.00	0.00	0.00	D-FMT	0.00
K05516	0.00	0.00	0.00	0.00	D-FMT	0.00
K05593	0.00	0.00	0.00	0.00	D-FMT	0.00
K05773	0.00	0.00	0.00	0.00	D-FMT	0.00
K05922	0.00	0.00	0.00	0.00	D-FMT	0.00
K06013	0.00	0.00	0.00	0.00	D-FMT	0.00
K06148	0.00	0.00	0.00	0.00	D-FMT	0.00
K06886	0.00	0.00	0.00	0.00	D-FMT	0.00
K07093	0.00	0.00	0.00	0.00	D-FMT	0.00
K07152	0.00	0.00	0.00	0.00	D-FMT	0.00
K07230	0.00	0.00	0.00	0.00	D-FMT	0.00
K07248	0.00	0.00	0.00	0.00	D-FMT	0.00
K07304	0.00	0.00	0.00	0.00	D-FMT	0.00
K07305	0.00	0.00	0.00	0.00	D-FMT	0.00
K07326	0.00	0.00	0.00	0.00	D-FMT	0.00
K07501	0.00	0.00	0.00	0.00	D-FMT	0.00
K07791	0.00	0.00	0.00	0.00	D-FMT	0.00
K07795	0.00	0.00	0.00	0.00	D-FMT	0.00
K08224	0.00	0.00	0.00	0.00	D-FMT	0.00
K08311	0.00	0.00	0.00	0.00	D-FMT	0.00

K09688	0.00	0.00	0.00	0.00	D-FMT	0.00
K09792	0.00	0.00	0.00	0.00	D-FMT	0.00
K09796	0.00	0.00	0.00	0.00	D-FMT	0.00
K09859	0.00	0.00	0.00	0.00	D-FMT	0.00
K09860	0.00	0.00	0.00	0.00	D-FMT	0.00
K09944	0.00	0.00	0.00	0.00	D-FMT	0.00
K09981	0.00	0.00	0.00	0.00	D-FMT	0.00
K10039	0.00	0.00	0.00	0.00	D-FMT	0.00
K10747	0.00	0.00	0.00	0.00	D-FMT	0.00
K10806	0.00	0.00	0.00	0.00	D-FMT	0.00
K10938	0.00	0.00	0.00	0.00	D-FMT	0.00
K11014	0.00	0.00	0.00	0.00	D-FMT	0.00
K11031	0.00	0.00	0.00	0.00	H-FMT	0.00
K11782	0.00	0.00	0.00	0.00	D-FMT	0.00
K11785	0.00	0.00	0.00	0.00	D-FMT	0.00
K13017	0.00	0.00	0.00	0.00	D-FMT	0.00
K13819	0.00	0.00	0.00	0.00	D-FMT	0.00
K14261	0.00	0.00	0.00	0.00	D-FMT	0.00
K14393	0.00	0.00	0.00	0.00	D-FMT	0.00
K15257	0.00	0.00	0.00	0.00	D-FMT	0.00
K15895	0.00	0.00	0.00	0.00	D-FMT	0.00
K15897	0.00	0.00	0.00	0.00	D-FMT	0.00
K15898	0.00	0.00	0.00	0.00	D-FMT	0.00
K15910	0.00	0.00	0.00	0.00	D-FMT	0.00
K15915	0.00	0.00	0.00	0.00	D-FMT	0.00
K16087	0.00	0.00	0.00	0.00	D-FMT	0.00
K17250	0.00	0.00	0.00	0.00	D-FMT	0.00
K17251	0.00	0.00	0.00	0.00	D-FMT	0.00
K18284	0.00	0.00	0.00	0.00	D-FMT	0.00
K18291	0.00	0.00	0.00	0.00	D-FMT	0.00
K18333	0.00	0.00	0.00	0.00	D-FMT	0.00
K18480	0.00	0.00	0.00	0.00	D-FMT	0.00
K18992	0.00	0.00	0.00	0.00	D-FMT	0.00
K19416	0.00	0.00	0.00	0.00	D-FMT	0.00
K22391	0.00	0.00	0.00	0.00	D-FMT	0.00
K22468	0.00	0.00	0.00	0.00	D-FMT	0.00
K00036	0.00	0.00	0.00	0.00	D-FMT	0.00
K00347	0.00	0.00	0.00	0.00	H-FMT	0.00
K00560	0.00	0.00	0.00	0.00	D-FMT	0.00
K00789	0.00	0.00	0.00	0.00	D-FMT	0.00
K00876	0.00	0.00	0.00	0.00	D-FMT	0.00
K00991	0.00	0.00	0.00	0.00	H-FMT	0.00
K01533	0.00	0.00	0.00	0.00	H-FMT	0.00
K01610	0.00	0.00	0.00	0.00	D-FMT	0.00

K01818	0.00	0.00	0.00	0.00	H-FMT	0.00
K01870	0.00	0.00	0.00	0.00	H-FMT	0.00
K01887	0.00	0.00	0.00	0.00	D-FMT	0.00
K01923	0.00	0.00	0.00	0.00	H-FMT	0.00
K02233	0.00	0.00	0.00	0.00	D-FMT	0.00
K02536	0.00	0.00	0.00	0.00	H-FMT	0.00
K02988	0.00	0.00	0.00	0.00	D-FMT	0.00
K03149	0.00	0.00	0.00	0.00	H-FMT	0.00
K03183	0.00	0.00	0.00	0.00	H-FMT	0.00
K03474	0.00	0.00	0.00	0.00	H-FMT	0.00
K06287	0.00	0.00	0.00	0.00	H-FMT	0.00
K06949	0.00	0.00	0.00	0.00	H-FMT	0.00
K11645	0.00	0.00	0.00	0.00	H-FMT	0.00
K13378	0.00	0.00	0.00	0.00	H-FMT	0.00
K14441	0.00	0.00	0.00	0.00	H-FMT	0.00
K15977	0.00	0.00	0.00	0.00	H-FMT	0.00
K19271	0.00	0.00	0.00	0.00	D-FMT	0.00
K21572	0.02	0.00	0.02	0.00	H-FMT	0.00
K00795	0.00	0.00	0.00	0.00	D-FMT	0.00
K01042	0.00	0.00	0.00	0.00	D-FMT	0.00
K01139	0.00	0.00	0.00	0.00	D-FMT	0.00
K01497	0.00	0.00	0.00	0.00	D-FMT	0.00
K01879	0.00	0.00	0.00	0.00	D-FMT	0.00
K02846	0.00	0.00	0.00	0.00	D-FMT	0.00
K03186	0.00	0.00	0.00	0.00	D-FMT	0.00
K03620	0.00	0.00	0.00	0.00	D-FMT	0.00
K03752	0.00	0.00	0.00	0.00	D-FMT	0.00
K03796	0.00	0.00	0.00	0.00	D-FMT	0.00
K03981	0.00	0.00	0.00	0.00	D-FMT	0.00
K04744	0.00	0.00	0.00	0.00	D-FMT	0.00
K05772	0.00	0.00	0.00	0.00	D-FMT	0.00
K06176	0.00	0.00	0.00	0.00	D-FMT	0.00
K06891	0.00	0.00	0.00	0.00	D-FMT	0.00
K09798	0.00	0.00	0.00	0.00	D-FMT	0.00
K12506	0.00	0.00	0.00	0.00	D-FMT	0.00
K15461	0.00	0.00	0.00	0.00	D-FMT	0.00
K17247	0.00	0.00	0.00	0.00	D-FMT	0.00
K00575	0.00	0.00	0.00	0.00	D-FMT	0.00
K01895	0.00	0.00	0.00	0.00	D-FMT	0.00
K02477	0.00	0.00	0.00	0.00	H-FMT	0.00
K03673	0.00	0.00	0.00	0.00	D-FMT	0.00
K03818	0.00	0.00	0.00	0.00	D-FMT	0.00
K06204	0.00	0.00	0.00	0.00	D-FMT	0.00
K06867	0.00	0.00	0.00	0.00	D-FMT	0.00

K08296	0.00	0.00	0.00	0.00	D-FMT	0.00
K11752	0.00	0.00	0.00	0.00	D-FMT	0.00
K02415	0.00	0.00	0.00	0.00	D-FMT	0.00
K03634	0.00	0.00	0.00	0.00	D-FMT	0.00
K07794	0.00	0.00	0.00	0.00	D-FMT	0.00
K10041	0.00	0.00	0.00	0.00	D-FMT	0.00
K11784	0.00	0.00	0.00	0.00	D-FMT	0.00
K00330	0.00	0.00	0.00	0.00	D-FMT	0.00
K00615	0.00	0.00	0.00	0.00	D-FMT	0.00
K00616	0.00	0.00	0.00	0.00	H-FMT	0.00
K00766	0.00	0.00	0.00	0.00	H-FMT	0.00
K00790	0.00	0.00	0.00	0.00	D-FMT	0.00
K01265	0.00	0.00	0.00	0.00	H-FMT	0.00
K01752	0.00	0.00	0.00	0.00	H-FMT	0.00
K01808	0.00	0.00	0.00	0.00	D-FMT	0.00
K02338	0.00	0.00	0.00	0.00	D-FMT	0.00
K02340	0.00	0.00	0.00	0.00	D-FMT	0.00
K02493	0.00	0.00	0.00	0.00	D-FMT	0.00
K02520	0.00	0.00	0.00	0.00	H-FMT	0.00
K02564	0.00	0.00	0.00	0.00	D-FMT	0.00
K02909	0.00	0.00	0.00	0.00	D-FMT	0.00
K03282	0.00	0.00	0.00	0.00	D-FMT	0.00
K03324	0.00	0.00	0.00	0.00	D-FMT	0.00
K03545	0.00	0.00	0.00	0.00	D-FMT	0.00
K03555	0.00	0.00	0.00	0.00	D-FMT	0.00
K03570	0.00	0.00	0.00	0.00	H-FMT	0.00
K03590	0.00	0.00	0.00	0.00	D-FMT	0.00
K03594	0.00	0.00	0.00	0.00	H-FMT	0.00
K03699	0.00	0.00	0.00	0.00	D-FMT	0.00
K06180	0.00	0.00	0.00	0.00	D-FMT	0.00
K06997	0.00	0.00	0.00	0.00	H-FMT	0.00
K07001	0.00	0.00	0.00	0.00	H-FMT	0.00
K07148	0.00	0.00	0.00	0.00	D-FMT	0.00
K09815	0.00	0.00	0.00	0.00	D-FMT	0.00
K09922	0.00	0.00	0.00	0.00	H-FMT	0.00
K10947	0.00	0.00	0.00	0.00	H-FMT	0.00
K11755	0.00	0.00	0.00	0.00	D-FMT	0.00
K16363	0.00	0.00	0.00	0.00	D-FMT	0.00
K01854	0.00	0.00	0.00	0.00	D-FMT	0.00
K03098	0.00	0.00	0.00	0.00	D-FMT	0.00
K06895	0.00	0.00	0.00	0.00	D-FMT	0.00
K11928	0.00	0.00	0.00	0.00	D-FMT	0.00
K00124	0.00	0.00	0.00	0.00	D-FMT	0.00
K01664	0.00	0.00	0.00	0.00	D-FMT	0.00

K03408	0.00	0.00	0.00	0.00	D-FMT	0.00
K16092	0.00	0.00	0.00	0.00	H-FMT	0.00
K01322	0.00	0.00	0.00	0.00	H-FMT	0.00
K00241	0.00	0.00	0.00	0.00	D-FMT	0.00
K01000	0.00	0.00	0.00	0.00	D-FMT	0.00
K01183	0.00	0.00	0.00	0.00	D-FMT	0.00
K01255	0.00	0.00	0.00	0.00	D-FMT	0.00
K01494	0.00	0.00	0.00	0.00	D-FMT	0.00
K02002	0.00	0.00	0.00	0.00	D-FMT	0.00
K03326	0.00	0.00	0.00	0.00	D-FMT	0.00
K03631	0.00	0.00	0.00	0.00	D-FMT	0.00
K04654	0.00	0.00	0.00	0.00	D-FMT	0.00
K06079	0.00	0.00	0.00	0.00	D-FMT	0.00
K07214	0.00	0.00	0.00	0.00	H-FMT	0.00
K08325	0.00	0.00	0.00	0.00	D-FMT	0.00
K09680	0.00	0.00	0.00	0.00	D-FMT	0.00
K19883	0.00	0.00	0.00	0.00	D-FMT	0.00
K00426	0.00	0.00	0.00	0.00	D-FMT	0.00
K00610	0.00	0.00	0.00	0.00	H-FMT	0.00
K00860	0.00	0.00	0.00	0.00	H-FMT	0.00
K01092	0.00	0.00	0.00	0.00	D-FMT	0.00
K01681	0.00	0.00	0.00	0.00	H-FMT	0.00
K01689	0.00	0.00	0.00	0.00	H-FMT	0.00
K01714	0.00	0.00	0.00	0.00	D-FMT	0.00
K01737	0.00	0.00	0.00	0.00	D-FMT	0.00
K02110	0.00	0.00	0.00	0.00	D-FMT	0.00
K02968	0.00	0.00	0.00	0.00	D-FMT	0.00
K03154	0.00	0.00	0.00	0.00	D-FMT	0.00
K03177	0.00	0.00	0.00	0.00	H-FMT	0.00
K03466	0.00	0.00	0.00	0.00	D-FMT	0.00
K05342	0.00	0.00	0.00	0.00	D-FMT	0.00
K09807	0.00	0.00	0.00	0.00	H-FMT	0.00
K21636	0.00	0.00	0.00	0.00	H-FMT	0.00
K02406	0.00	0.00	0.00	0.00	D-FMT	0.00
K13979	0.00	0.00	0.00	0.00	D-FMT	0.00
K00407	0.00	0.00	0.00	0.00	D-FMT	0.00
K00411	0.00	0.00	0.00	0.00	D-FMT	0.00
K00549	0.00	0.00	0.00	0.00	D-FMT	0.00
K02573	0.00	0.00	0.00	0.00	D-FMT	0.00
K05927	0.00	0.00	0.00	0.00	D-FMT	0.00
K07084	0.00	0.00	0.00	0.00	D-FMT	0.00
K07127	0.00	0.00	0.00	0.00	D-FMT	0.00
K07457	0.00	0.00	0.00	0.00	D-FMT	0.00
K09943	0.00	0.00	0.00	0.00	D-FMT	0.00

K15894	0.00	0.00	0.00	0.00	D-FMT	0.00
K15899	0.00	0.00	0.00	0.00	D-FMT	0.00
K18285	0.00	0.00	0.00	0.00	D-FMT	0.00
K18292	0.00	0.00	0.00	0.00	D-FMT	0.00
K00128	0.00	0.00	0.00	0.00	H-FMT	0.00
K07109	0.00	0.00	0.00	0.00	H-FMT	0.00
K06857	0.00	0.00	0.00	0.00	D-FMT	0.00
K00784	0.00	0.00	0.00	0.00	H-FMT	0.00
K04518	0.00	0.00	0.00	0.00	H-FMT	0.00
K00606	0.00	0.00	0.00	0.00	D-FMT	0.00
K00831	0.00	0.00	0.00	0.00	H-FMT	0.00
K00854	0.00	0.00	0.00	0.00	D-FMT	0.00
K00901	0.00	0.00	0.00	0.00	D-FMT	0.00
K01069	0.00	0.00	0.00	0.00	D-FMT	0.00
K01201	0.00	0.00	0.00	0.00	H-FMT	0.00
K01206	0.00	0.00	0.00	0.00	D-FMT	0.00
K01338	0.00	0.00	0.00	0.00	D-FMT	0.00
K01783	0.00	0.00	0.00	0.00	D-FMT	0.00
K01892	0.00	0.00	0.00	0.00	H-FMT	0.00
K01924	0.00	0.00	0.00	0.00	D-FMT	0.00
K02316	0.00	0.00	0.00	0.00	D-FMT	0.00
K02377	0.00	0.00	0.00	0.00	D-FMT	0.00
K02876	0.00	0.00	0.00	0.00	D-FMT	0.00
K02897	0.00	0.00	0.00	0.00	D-FMT	0.00
K02956	0.00	0.00	0.00	0.00	D-FMT	0.00
K03216	0.00	0.00	0.00	0.00	D-FMT	0.00
K03402	0.00	0.00	0.00	0.00	D-FMT	0.00
K03530	0.00	0.00	0.00	0.00	D-FMT	0.00
K03977	0.00	0.00	0.00	0.00	D-FMT	0.00
K04047	0.00	0.00	0.00	0.00	D-FMT	0.00
K04084	0.00	0.00	0.00	0.00	H-FMT	0.00
K04564	0.00	0.00	0.00	0.00	H-FMT	0.00
K09888	0.00	0.00	0.00	0.00	H-FMT	0.00
K12140	0.00	0.00	0.00	0.00	H-FMT	0.00
K13990	0.00	0.00	0.00	0.00	H-FMT	0.00
K17828	0.00	0.00	0.00	0.00	D-FMT	0.00
K18979	0.00	0.00	0.00	0.00	H-FMT	0.00
K21750	0.00	0.00	0.00	0.00	H-FMT	0.00
K01507	0.00	0.00	0.00	0.00	D-FMT	0.00
K14623	0.00	0.00	0.00	0.00	H-FMT	0.00
K00123	0.00	0.00	0.00	0.00	D-FMT	0.00
K02568	0.00	0.00	0.00	0.00	D-FMT	0.00
K02841	0.00	0.00	0.00	0.00	D-FMT	0.00
K03635	0.00	0.00	0.00	0.00	D-FMT	0.00

K03841	0.00	0.00	0.00	0.00	D-FMT	0.00
K07147	0.00	0.00	0.00	0.00	D-FMT	0.00
K15256	0.00	0.00	0.00	0.00	D-FMT	0.00
K07112	0.00	0.00	0.00	0.00	D-FMT	0.00
K00980	0.00	0.00	0.00	0.00	D-FMT	0.00
K06193	0.00	0.00	0.00	0.00	D-FMT	0.00
K08169	0.00	0.00	0.00	0.00	H-FMT	0.00
K02437	0.00	0.00	0.00	0.00	H-FMT	0.00
K00929	0.00	0.00	0.00	0.00	D-FMT	0.00
K01205	0.00	0.00	0.00	0.00	H-FMT	0.00
K01546	0.00	0.00	0.00	0.00	D-FMT	0.00
K01591	0.00	0.00	0.00	0.00	D-FMT	0.00
K02005	0.00	0.00	0.00	0.00	H-FMT	0.00
K02518	0.01	0.00	0.00	0.00	D-FMT	0.00
K02874	0.00	0.00	0.00	0.00	D-FMT	0.00
K02939	0.00	0.00	0.00	0.00	D-FMT	0.00
K03584	0.00	0.00	0.00	0.00	H-FMT	0.00
K03589	0.00	0.00	0.00	0.00	H-FMT	0.00
K03629	0.00	0.00	0.00	0.00	D-FMT	0.00
K03664	0.00	0.00	0.00	0.00	H-FMT	0.00
K04079	0.00	0.00	0.00	0.00	H-FMT	0.00
K07483	0.00	0.00	0.00	0.00	D-FMT	0.00
K07507	0.00	0.00	0.00	0.00	D-FMT	0.00
K09861	0.00	0.00	0.00	0.00	D-FMT	0.00
K21071	0.00	0.00	0.00	0.00	H-FMT	0.00
K03820	0.00	0.00	0.00	0.00	H-FMT	0.00
K01679	0.00	0.00	0.00	0.00	D-FMT	0.00
K10040	0.00	0.00	0.00	0.00	D-FMT	0.00
K03932	0.00	0.00	0.00	0.00	H-FMT	0.00
K01821	0.00	0.00	0.00	0.00	D-FMT	0.00
K01665	0.00	0.00	0.00	0.00	D-FMT	0.00
K13695	0.00	0.00	0.00	0.00	D-FMT	0.00
K00556	0.00	0.00	0.00	0.00	H-FMT	0.00
K02408	0.00	0.00	0.00	0.00	D-FMT	0.00
K16153	0.00	0.00	0.00	0.00	D-FMT	0.00
K00803	0.00	0.00	0.00	0.00	H-FMT	0.00
K00339	0.00	0.00	0.00	0.00	H-FMT	0.00
K00652	0.00	0.00	0.00	0.00	H-FMT	0.00
K00759	0.00	0.00	0.00	0.00	H-FMT	0.00
K00820	0.00	0.00	0.00	0.00	D-FMT	0.00
K00939	0.00	0.00	0.00	0.00	H-FMT	0.00
K00954	0.00	0.00	0.00	0.00	H-FMT	0.00
K01790	0.00	0.00	0.00	0.00	D-FMT	0.00
K01835	0.00	0.00	0.00	0.00	H-FMT	0.00

K01869	0.00	0.00	0.00	0.00	H-FMT	0.00
K01993	0.00	0.00	0.00	0.00	H-FMT	0.00
K02313	0.00	0.00	0.00	0.00	H-FMT	0.00
K02838	0.00	0.00	0.00	0.00	H-FMT	0.00
K02961	0.00	0.00	0.00	0.00	D-FMT	0.00
K03147	0.00	0.00	0.00	0.00	D-FMT	0.00
K03469	0.00	0.00	0.00	0.00	D-FMT	0.00
K03624	0.00	0.00	0.00	0.00	D-FMT	0.00
K03816	0.00	0.00	0.00	0.00	H-FMT	0.00
K04764	0.00	0.00	0.00	0.00	D-FMT	0.00
K06406	0.00	0.00	0.00	0.00	D-FMT	0.00
K06956	0.00	0.00	0.00	0.00	D-FMT	0.00
K07164	0.00	0.00	0.00	0.00	H-FMT	0.00
K07793	0.00	0.00	0.00	0.00	D-FMT	0.00
K08303	0.00	0.00	0.00	0.00	D-FMT	0.00
K12410	0.00	0.00	0.00	0.00	D-FMT	0.00
K13789	0.00	0.00	0.00	0.00	D-FMT	0.00
K19299	0.00	0.00	0.00	0.00	H-FMT	0.00
K22477	0.00	0.00	0.00	0.00	D-FMT	0.00
K03532	0.00	0.00	0.00	0.00	D-FMT	0.00
K05797	0.00	0.00	0.00	0.00	D-FMT	0.00
K08297	0.00	0.00	0.00	0.00	H-FMT	0.00
K11382	0.00	0.00	0.00	0.00	D-FMT	0.00
K11743	0.00	0.00	0.00	0.00	D-FMT	0.00
K13004	0.00	0.00	0.00	0.00	H-FMT	0.00
K20490	0.00	0.00	0.00	0.00	D-FMT	0.00
K01195	0.00	0.00	0.00	0.00	H-FMT	0.00
K00872	0.00	0.00	0.00	0.00	D-FMT	0.00
K06929	0.00	0.00	0.00	0.00	D-FMT	0.00
K19545	0.00	0.00	0.00	0.00	H-FMT	0.00
K21993	0.00	0.00	0.00	0.00	H-FMT	0.00
K02401	0.00	0.00	0.00	0.00	D-FMT	0.00
K09707	0.00	0.00	0.00	0.00	D-FMT	0.00
K19050	0.00	0.00	0.00	0.00	H-FMT	0.00
K00350	0.00	0.00	0.00	0.00	H-FMT	0.00
K00764	0.00	0.00	0.00	0.00	D-FMT	0.00
K01012	0.00	0.00	0.00	0.00	D-FMT	0.00
K01119	0.00	0.00	0.00	0.00	H-FMT	0.00
K01163	0.00	0.00	0.00	0.00	H-FMT	0.00
K01678	0.00	0.00	0.00	0.00	H-FMT	0.00
K02473	0.00	0.00	0.00	0.00	D-FMT	0.00
K02570	0.00	0.00	0.00	0.00	D-FMT	0.00
K02867	0.00	0.00	0.00	0.00	D-FMT	0.00
K03294	0.00	0.00	0.00	0.00	D-FMT	0.00

K03296	0.00	0.00	0.00	0.00	H-FMT	0.00
K03313	0.00	0.00	0.00	0.00	D-FMT	0.00
K03320	0.00	0.00	0.00	0.00	D-FMT	0.00
K03801	0.00	0.00	0.00	0.00	H-FMT	0.00
K03925	0.00	0.00	0.00	0.00	H-FMT	0.00
K06173	0.00	0.00	0.00	0.00	D-FMT	0.00
K07216	0.00	0.00	0.00	0.00	D-FMT	0.00
K18369	0.00	0.00	0.00	0.00	H-FMT	0.01
K00641	0.00	0.00	0.00	0.00	H-FMT	0.01
K09740	0.00	0.00	0.00	0.00	H-FMT	0.01
K01838	0.00	0.00	0.00	0.00	H-FMT	0.01
K02392	0.00	0.00	0.00	0.00	D-FMT	0.01
K03817	0.00	0.00	0.00	0.00	H-FMT	0.01
K03306	0.00	0.00	0.00	0.00	D-FMT	0.01
K00009	0.00	0.00	0.00	0.00	D-FMT	0.01
K00382	0.00	0.00	0.00	0.00	H-FMT	0.01
K00384	0.00	0.00	0.00	0.00	D-FMT	0.01
K00548	0.00	0.00	0.00	0.00	H-FMT	0.01
K00600	0.00	0.00	0.00	0.00	D-FMT	0.01
K00651	0.00	0.00	0.00	0.00	D-FMT	0.01
K00773	0.00	0.00	0.00	0.00	D-FMT	0.01
K00800	0.00	0.00	0.00	0.00	H-FMT	0.01
K00817	0.00	0.00	0.00	0.00	H-FMT	0.01
K00971	0.00	0.00	0.00	0.00	D-FMT	0.01
K01081	0.00	0.00	0.00	0.00	H-FMT	0.01
K01190	0.00	0.00	0.00	0.00	H-FMT	0.01
K01209	0.00	0.00	0.00	0.00	H-FMT	0.01
K01537	0.00	0.00	0.00	0.00	H-FMT	0.01
K01548	0.00	0.00	0.00	0.00	D-FMT	0.01
K01579	0.00	0.00	0.00	0.00	D-FMT	0.01
K01712	0.00	0.00	0.00	0.00	H-FMT	0.01
K01733	0.00	0.00	0.00	0.00	H-FMT	0.01
K01811	0.00	0.00	0.00	0.00	H-FMT	0.01
K01890	0.00	0.00	0.00	0.00	D-FMT	0.01
K01997	0.00	0.00	0.00	0.00	D-FMT	0.01
K02067	0.00	0.00	0.00	0.00	H-FMT	0.01
K02108	0.00	0.00	0.00	0.00	D-FMT	0.01
K02109	0.00	0.00	0.00	0.00	D-FMT	0.01
K02121	0.00	0.00	0.00	0.00	D-FMT	0.01
K02371	0.00	0.00	0.00	0.00	H-FMT	0.01
K02884	0.00	0.00	0.00	0.00	D-FMT	0.01
K02895	0.00	0.00	0.00	0.00	D-FMT	0.01
K03284	0.00	0.00	0.00	0.00	D-FMT	0.01
K03525	0.00	0.00	0.00	0.00	D-FMT	0.01

K03648	0.00	0.00	0.00	0.00	H-FMT	0.01
K03734	0.00	0.00	0.00	0.00	D-FMT	0.01
K03778	0.00	0.00	0.00	0.00	D-FMT	0.01
K06881	0.00	0.00	0.00	0.00	H-FMT	0.01
K07027	0.00	0.00	0.00	0.00	H-FMT	0.01
K07133	0.00	0.00	0.00	0.00	H-FMT	0.01
K07636	0.00	0.00	0.00	0.00	H-FMT	0.01
K07646	0.00	0.00	0.00	0.00	D-FMT	0.01
K07812	0.00	0.00	0.00	0.00	D-FMT	0.01
K08156	0.00	0.00	0.00	0.00	H-FMT	0.01
K09794	0.00	0.00	0.00	0.00	D-FMT	0.01
K09955	0.00	0.00	0.00	0.00	H-FMT	0.01
K11069	0.00	0.00	0.00	0.00	H-FMT	0.01
K12343	0.00	0.00	0.00	0.00	H-FMT	0.01
K15531	0.00	0.00	0.00	0.00	H-FMT	0.01
K15532	0.00	0.00	0.00	0.00	H-FMT	0.01
K21557	0.00	0.00	0.00	0.00	D-FMT	0.01
K00226	0.00	0.00	0.00	0.00	D-FMT	0.01
K00691	0.00	0.00	0.00	0.00	H-FMT	0.01
K12141	0.00	0.00	0.00	0.00	H-FMT	0.01
K07474	0.00	0.00	0.00	0.00	H-FMT	0.01
K13653	0.00	0.00	0.00	0.00	H-FMT	0.01
K00425	0.00	0.00	0.00	0.00	H-FMT	0.01
K00656	0.00	0.00	0.00	0.00	D-FMT	0.01
K00674	0.00	0.00	0.00	0.00	D-FMT	0.01
K00850	0.00	0.00	0.00	0.00	D-FMT	0.01
K01991	0.00	0.00	0.00	0.00	H-FMT	0.01
K02004	0.00	0.00	0.00	0.00	H-FMT	0.01
K02232	0.00	0.00	0.00	0.00	D-FMT	0.01
K02444	0.00	0.00	0.00	0.00	D-FMT	0.01
K02483	0.00	0.00	0.00	0.00	D-FMT	0.01
K02529	0.00	0.00	0.00	0.00	H-FMT	0.01
K02860	0.00	0.00	0.00	0.00	D-FMT	0.01
K02878	0.00	0.00	0.00	0.00	D-FMT	0.01
K02994	0.00	0.00	0.00	0.00	D-FMT	0.01
K03308	0.00	0.00	0.00	0.00	D-FMT	0.01
K03340	0.00	0.00	0.00	0.00	D-FMT	0.01
K07240	0.00	0.00	0.00	0.00	H-FMT	0.01
K07407	0.00	0.00	0.00	0.00	H-FMT	0.01
K07469	0.00	0.00	0.00	0.00	H-FMT	0.01
K08138	0.00	0.00	0.00	0.00	H-FMT	0.01
K08289	0.00	0.00	0.00	0.00	D-FMT	0.01
K08307	0.00	0.00	0.00	0.00	H-FMT	0.01
K09758	0.00	0.00	0.00	0.00	H-FMT	0.01

K10773	0.00	0.00	0.00	0.00	D-FMT	0.01
K11070	0.00	0.00	0.00	0.00	D-FMT	0.01
K13043	0.00	0.00	0.00	0.00	D-FMT	0.01
K19955	0.00	0.00	0.00	0.00	H-FMT	0.01
K00261	0.00	0.00	0.00	0.00	D-FMT	0.01
K02421	0.00	0.00	0.00	0.00	D-FMT	0.01
K07397	0.00	0.00	0.00	0.00	H-FMT	0.01
K01208	0.00	0.00	0.00	0.00	D-FMT	0.01
K07160	0.00	0.00	0.00	0.00	D-FMT	0.01
K11013	0.00	0.00	0.00	0.00	D-FMT	0.01
K15772	0.00	0.00	0.00	0.00	D-FMT	0.01
K15896	0.00	0.00	0.00	0.00	D-FMT	0.01
K18817	0.00	0.00	0.00	0.00	D-FMT	0.01
K19276	0.00	0.00	0.00	0.00	D-FMT	0.01
K22424	0.00	0.00	0.00	0.00	D-FMT	0.01
K00170	0.00	0.00	0.00	0.00	H-FMT	0.01
K16898	0.00	0.00	0.00	0.00	D-FMT	0.01

Supplementary Table 3: Piglet information statistics table

Piglet ID	Breed	Age (day)	Gender	Body weight (kg)
diarrhea-1	DLY	8	female	3.25
health-1	DLY	8	female	3.43
diarrhea-2	DLY	10	female	3.76
health-2	DLY	10	female	3.87
diarrhea-3	DLY	10	male	3.92
health-3	DLY	10	male	4.03
diarrhea-4	DLY	11	female	4.12
health-4	DLY	11	female	3.98
diarrhea-5	DLY	10	female	3.88
health-5	DLY	10	female	3.67
diarrhea-6	DLY	10	male	3.95
health-6	DLY	10	male	3.89
diarrhea-7	DLY	10	female	3.58
health-7	DLY	10	female	3.77
diarrhea-8	DLY	10	female	3.66
health-8	DLY	10	female	3.59
diarrhea-9	DLY	10	male	3.89
health-9	DLY	10	male	4.08
diarrhea-10	DLY	10	female	3.76
health-10	DLY	10	female	3.83
diarrhea-11	DLY	10	female	3.46
health-11	DLY	10	female	4.02
diarrhea-12	DLY	10	male	3.46
health-12	DLY	10	male	3.34

diarrhea-13	DLY	10	male	4.23
health-13	DLY	10	male	4.19
diarrhea-14	DLY	11	male	3.95
health-14	DLY	11	male	3.77
diarrhea-15	DLY	11	female	4.57
health-15	DLY	11	female	4.63
diarrhea-16	DLY	11	female	4.38
health-16	DLY	11	female	4.21
diarrhea-17	DLY	11	male	4.29
health-17	DLY	11	male	4.16
diarrhea-18	DLY	9	female	3.34
health-18	DLY	9	female	3.53
diarrhea-19	DLY	9	female	3.27
health-19	DLY	9	female	3.62
diarrhea-20	DLY	10	male	3.58
health-20	DLY	10	male	3.42
diarrhea-21	DLY	10	male	3.67
health-21	DLY	10	male	3.33
diarrhea-22	DLY	8	male	2.48
health-22	DLY	8	male	3.06
diarrhea-23	DLY	8	male	3.15
health-23	DLY	8	male	2.83
diarrhea-24	DLY	8	male	3.38
health-24	DLY	8	male	3.86
diarrhea-25	DLY	8	female	2.58
health-25	DLY	8	female	2.71
diarrhea-26	DLY	8	male	3.16
health-26	DLY	8	male	3.23
diarrhea-27	DLY	8	male	3.57
health-27	DLY	8	male	2.93
diarrhea-28	DLY	8	male	3.31
health-28	DLY	8	male	3.24
diarrhea-29	DLY	8	male	3.62
health-29	DLY	8	male	3.73
diarrhea-30	DLY	9	male	3.86
health-30	DLY	9	male	3.97

Note: DLY=Duroc × Landrace × Yorkshire

Original blots presented in the manuscript

Supplementary Figure 11: Original blots presented in the Figure 4E ZO-1 (Ileum)

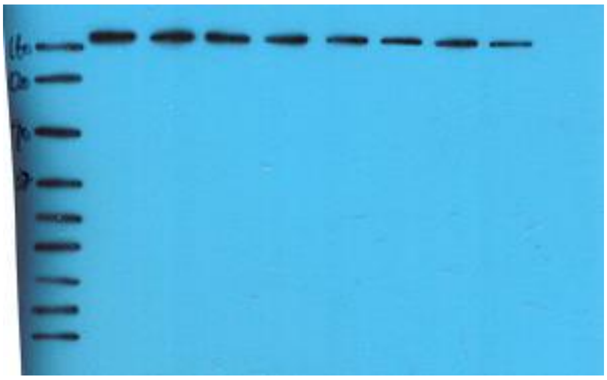

Supplementary Figure 12: Original blots presented in the Figure 4E Occludin (Ileum)

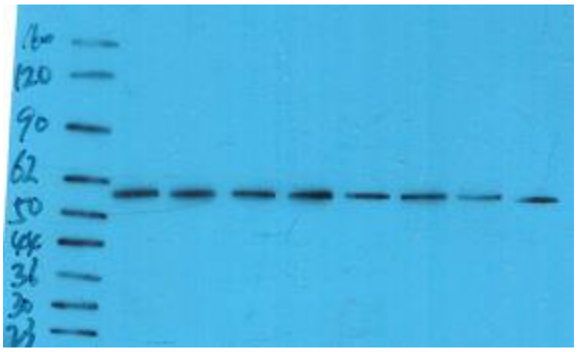

Supplementary Figure 13: Original blots presented in the Figure 4E p-AKT (Ileum)

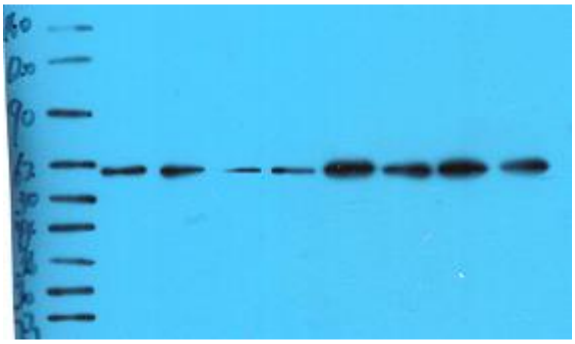

Supplementary Figure 14: Original blots presented in the Figure 4E AKT (Ileum)

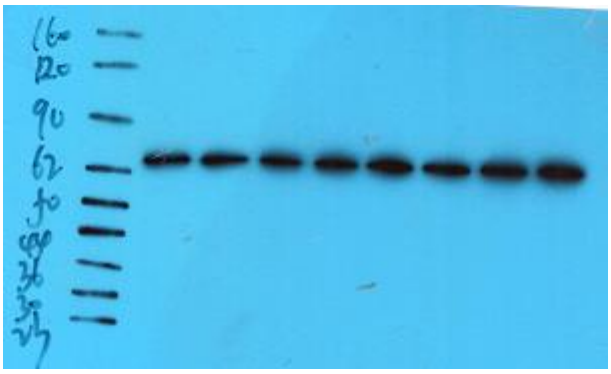

Supplementary Figure 15: Original blots presented in the Figure 4E p-NF-κB (Ileum)

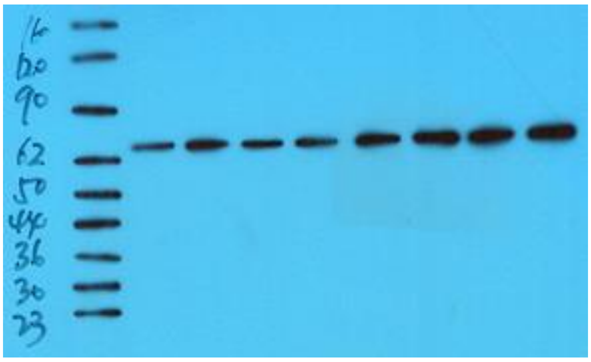

Supplementary Figure 16: Original blots presented in the Figure 4E NF-κB (Ileum)

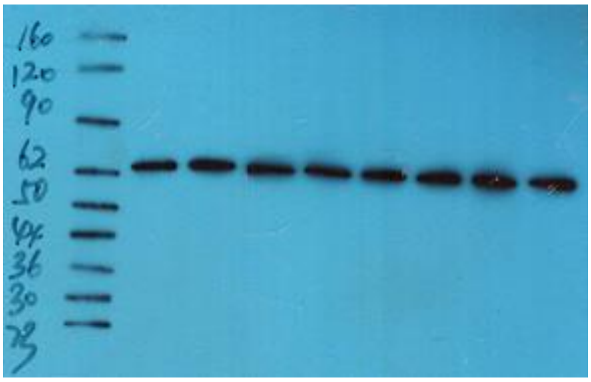

Supplementary Figure 17: Original blots presented in the Figure 4E β-actin (Ileum)

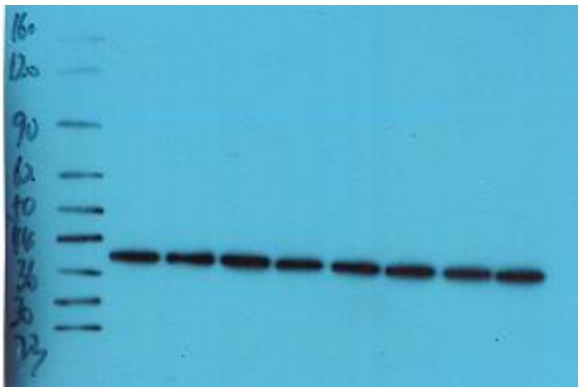

Supplementary Figure 18: Original blots presented in the Figure 4E ZO-1 (Colon)

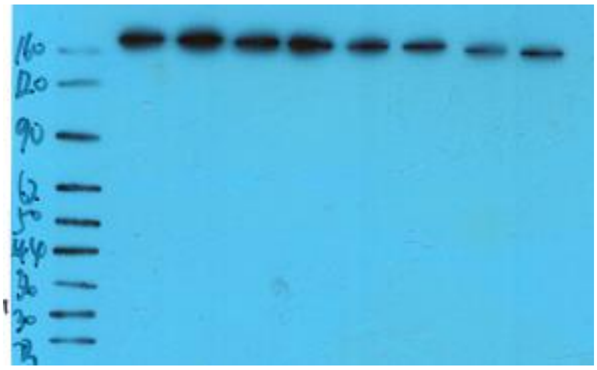

Supplementary Figure 19: Original blots presented in the Figure 4E Occludin (Colon)

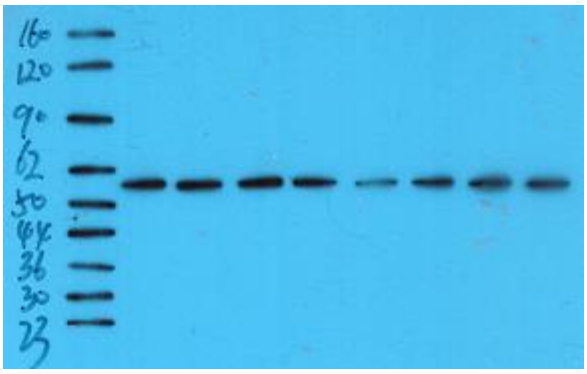

Supplementary Figure 20: Original blots presented in the Figure 4E p-AKT (Colon)

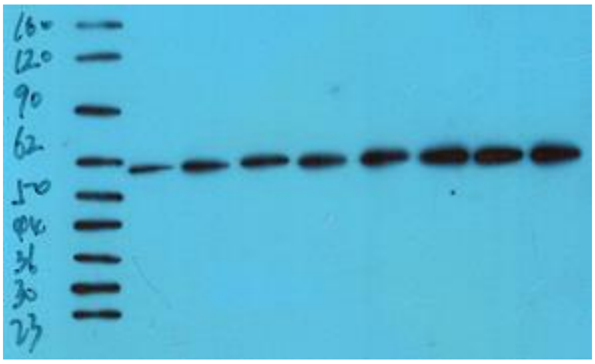

Supplementary Figure 21: Original blots presented in the Figure 4E AKT (Colon)

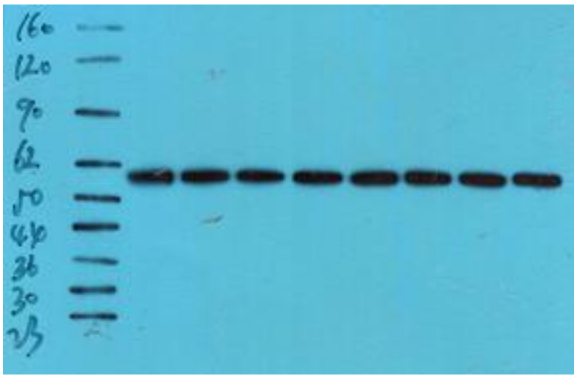

Supplementary Figure 22: Original blots presented in the Figure 4E p-NF-κB (Colon)

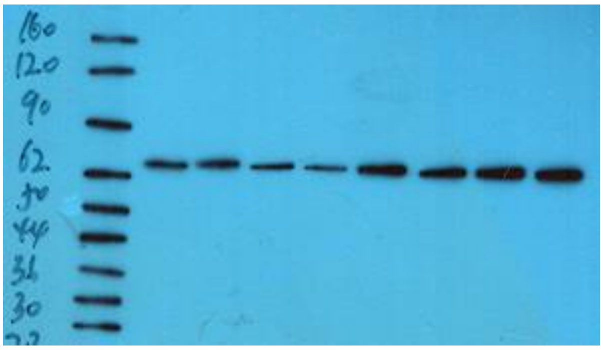

Supplementary Figure 23: Original blots presented in the Figure 4E NF-κB (Colon)

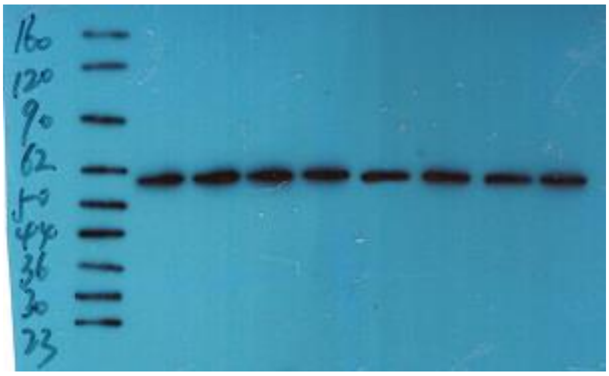

Supplementary Figure 24: Original blots presented in the Figure 4E β-actin (Colon)

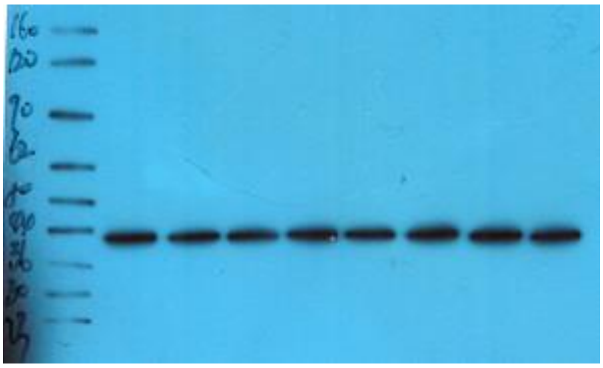

Supplementary Figure 25: Original blots presented in the Figure 5G ZO-1 (Ileum)

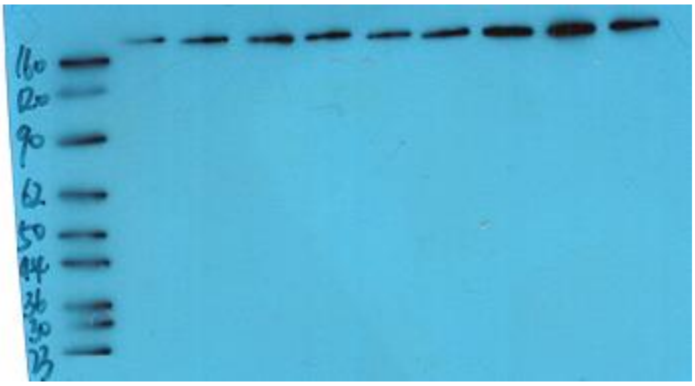

Supplementary Figure 26: Original blots presented in the Figure 5G Occludin (Ileum)

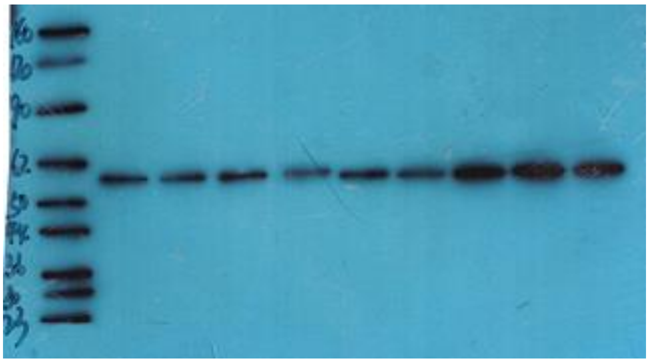

Supplementary Figure 27: Original blots presented in the Figure 5G p-AKT (Ileum)

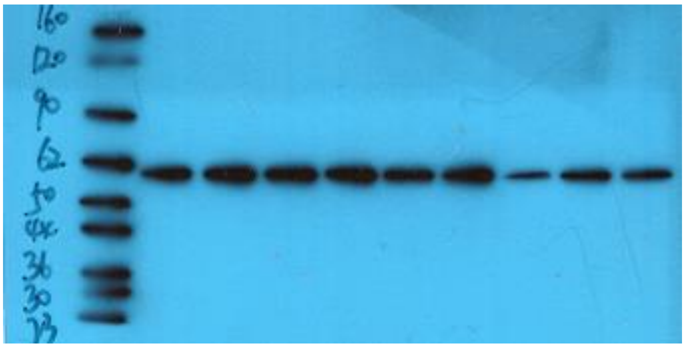

Supplementary Figure 28: Original blots presented in the Figure 5G AKT (Ileum)

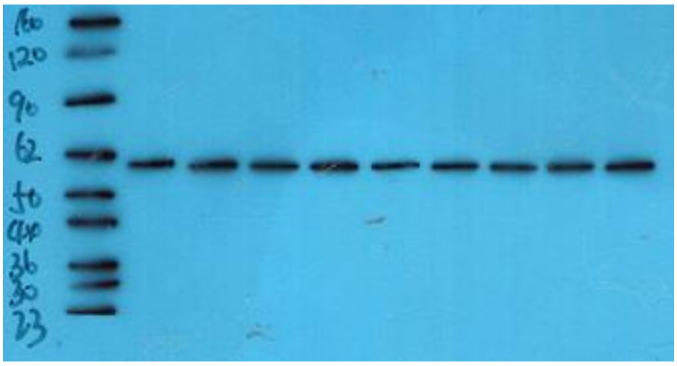

Supplementary Figure 29: Original blots presented in the Figure 5G p-NF-κB (Ileum)

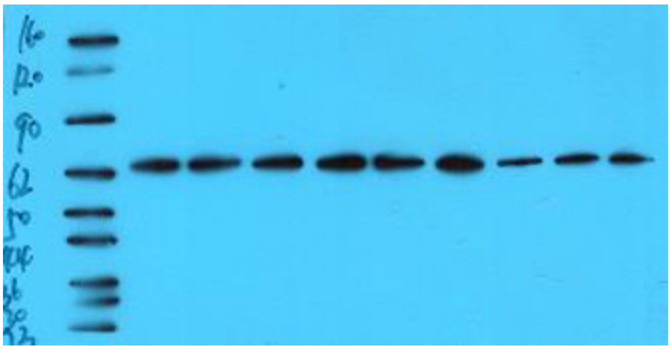

Supplementary Figure 30: Original blots presented in the Figure 5G NF-κB (Ileum)

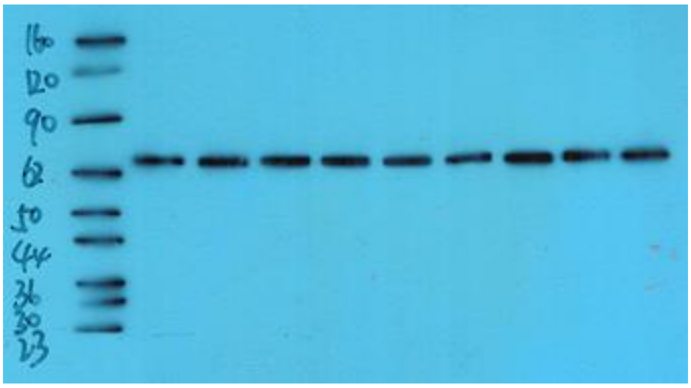

Supplementary Figure 31: Original blots presented in the Figure 5G β-actin (Ileum)

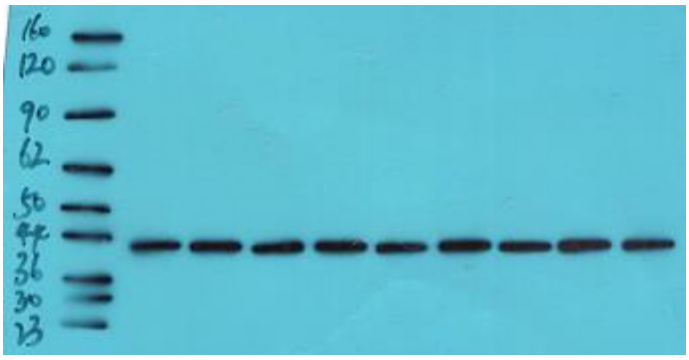

Supplementary Figure 32: Original blots presented in the Figure 5G ZO-1 (Colon)

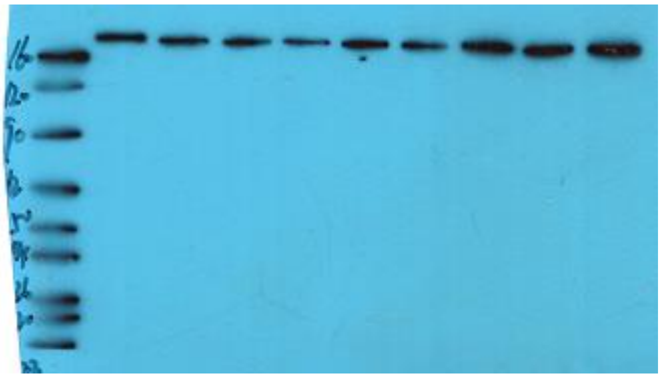

Supplementary Figure 33: Original blots presented in the Figure 5G Occludin (Colon)

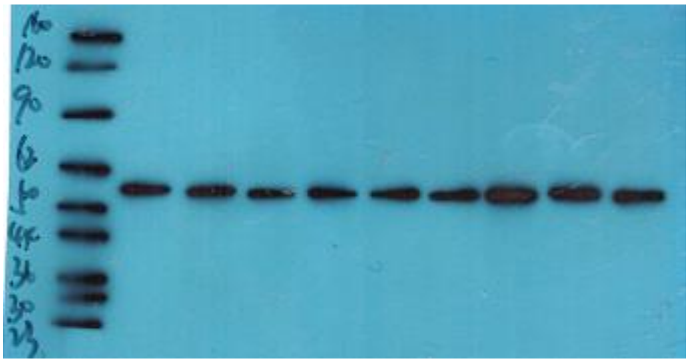

Supplementary Figure 34: Original blots presented in the Figure 5G p-AKT (Colon)

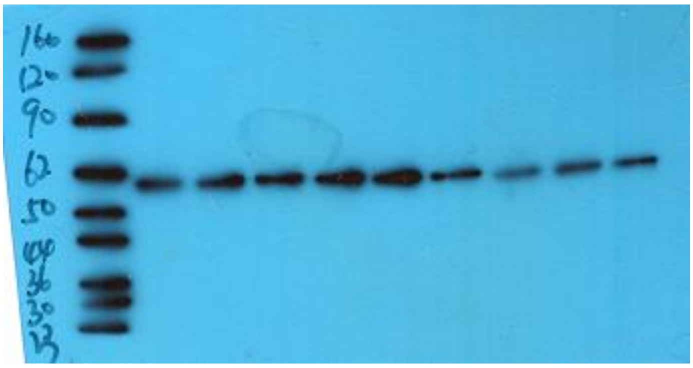

Supplementary Figure 35: Original blots presented in the Figure 5G AKT (Colon)

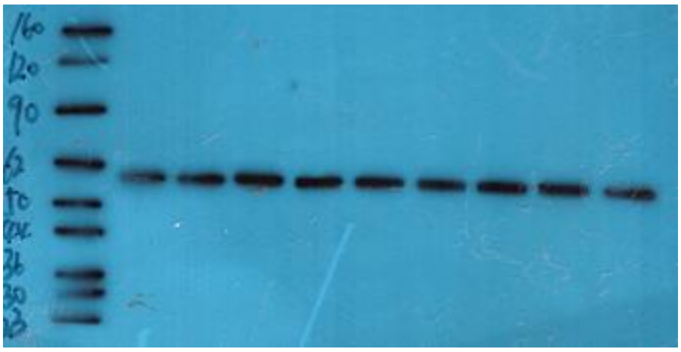

Supplementary Figure 36: Original blots presented in the Figure 5G p-NF-κB (Colon)

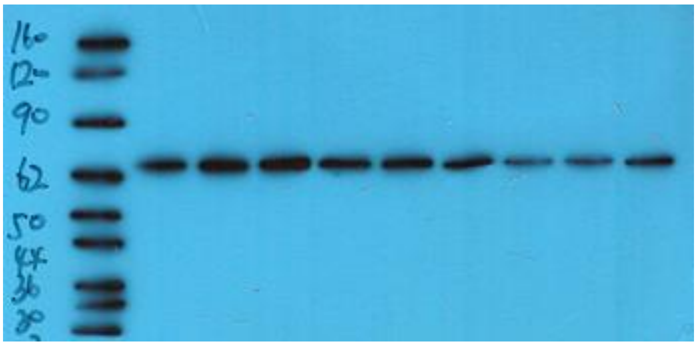

Supplementary Figure 37: Original blots presented in the Figure 5G NF-κB (Colon)

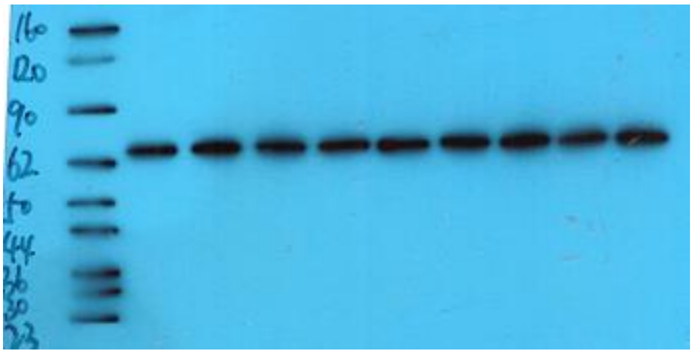

Supplementary Figure 38: Original blots presented in the Figure 5G β-actin (Colon)

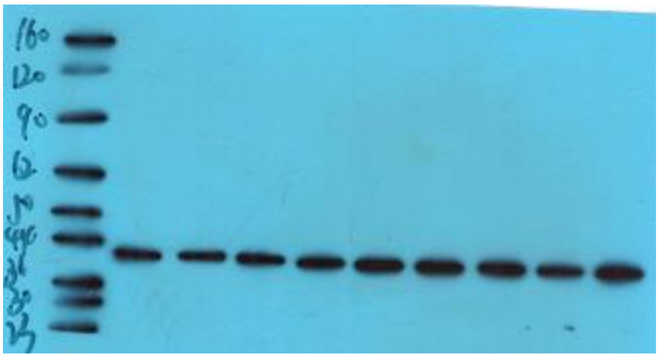

Supplementary Figure 39: Original blots presented in the Figure 6F ZO-1 (Ileum)

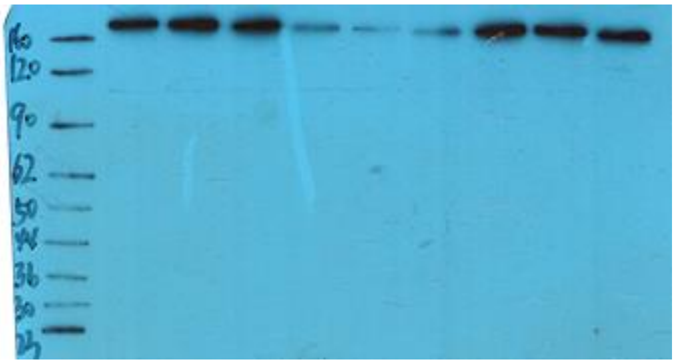

Supplementary Figure 40: Original blots presented in the Figure 6F Occludin (Ileum)

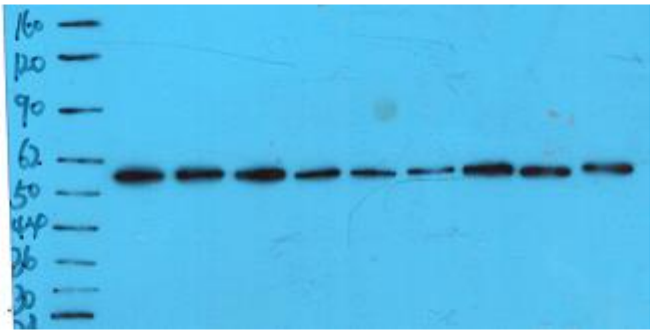

Supplementary Figure 41: Original blots presented in the Figure 6F p-AKT (Ileum)

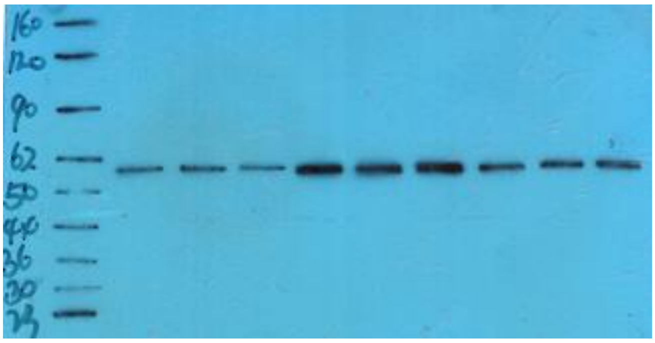

Supplementary Figure 42: Original blots presented in the Figure 6F AKT (Ileum)

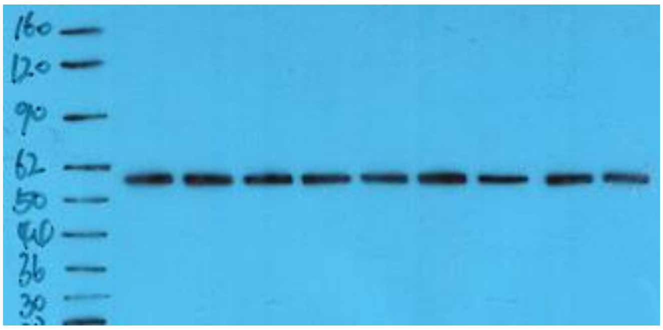

Supplementary Figure 43: Original blots presented in the Figure 6F p-NF-κB (Ileum)

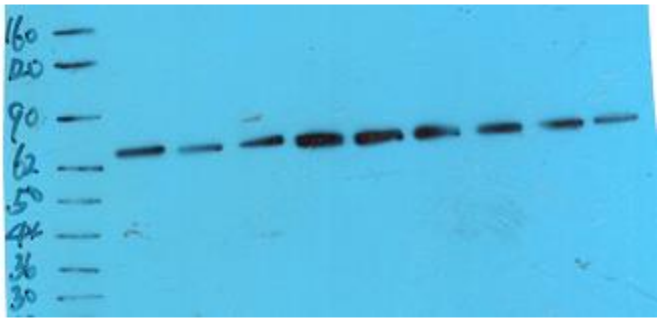

Supplementary Figure 44: Original blots presented in the Figure 6F NF-κB (Ileum)

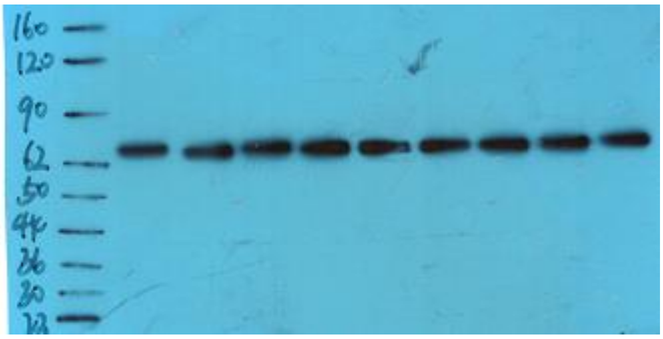

Supplementary Figure 45: Original blots presented in the Figure 6F β-actin (Ileum)

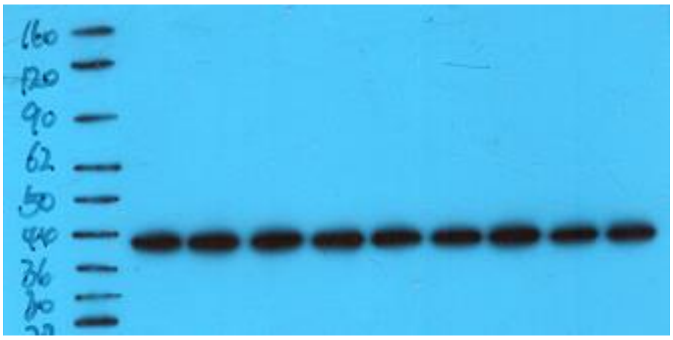

Supplementary Figure 46: Original blots presented in the Figure 6F ZO-1 (Colon)

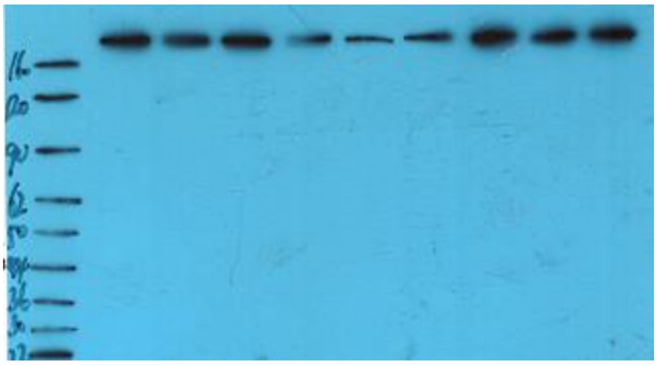

Supplementary Figure 47: Original blots presented in the Figure 6F Occludin (Colon)

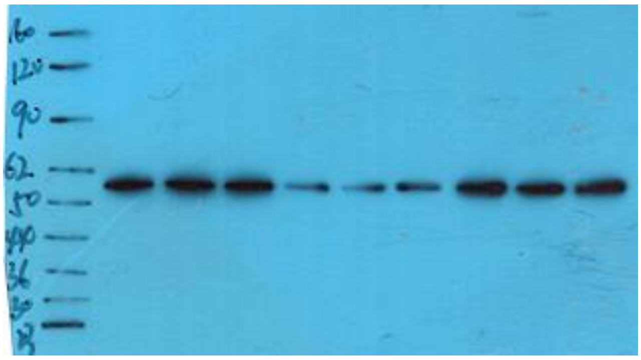

Supplementary Figure 48: Original blots presented in the Figure 6F p-AKT (Colon)

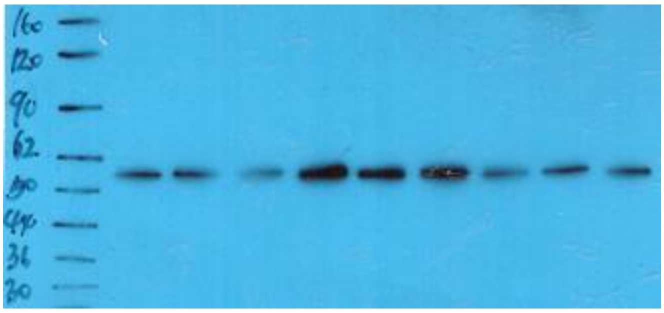

Supplementary Figure 49: Original blots presented in the Figure 6F AKT (Colon)

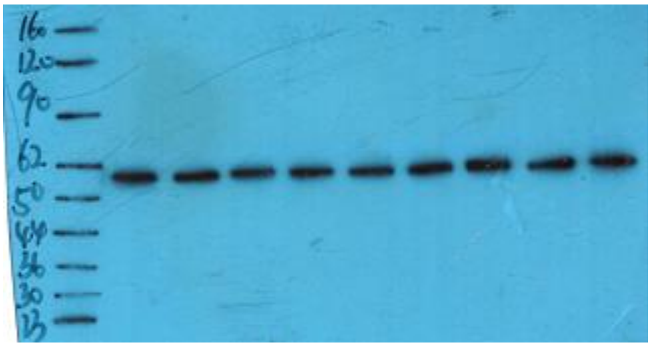

Supplementary Figure 50: Original blots presented in the Figure 6F p-NF-κB (Colon)

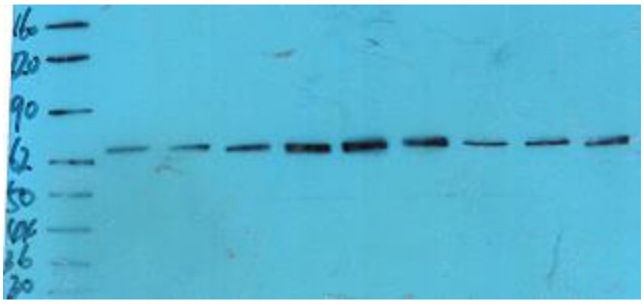

Supplementary Figure 51: Original blots presented in the Figure 6F NF-κB (Colon)

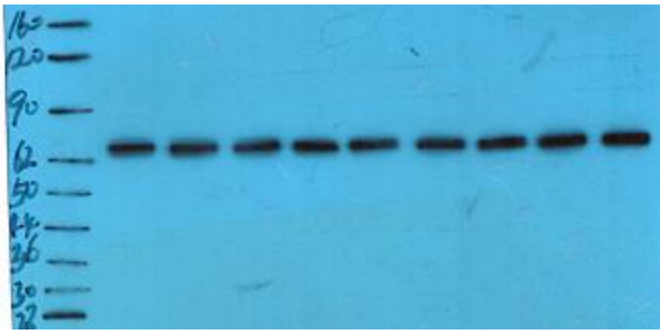

Supplementary Figure 52: Original blots presented in the Figure 6F β-actin (Colon)

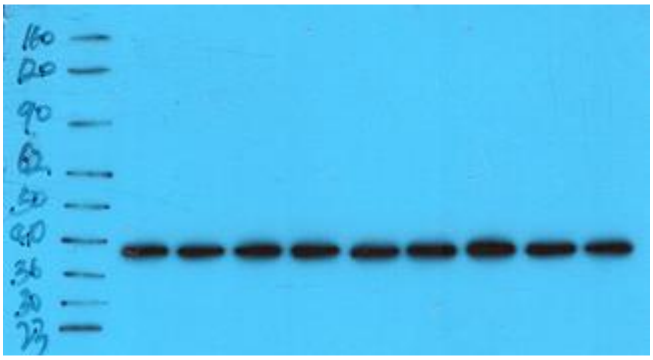

Supplementary Figure 53: Original blots presented in the Figure 7H iNOS (Ileum)

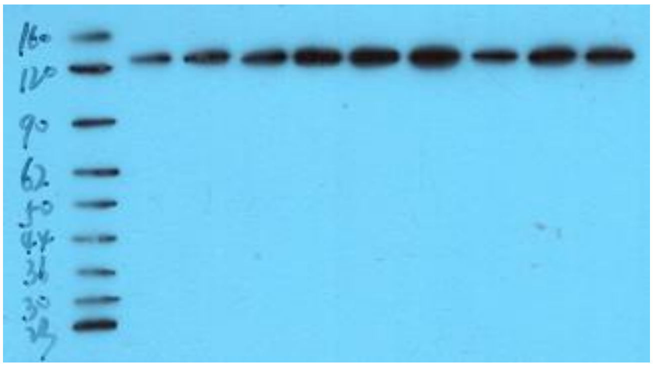

Supplementary Figure 54: Original blots presented in the Figure 7H Arg1 (Ileum)

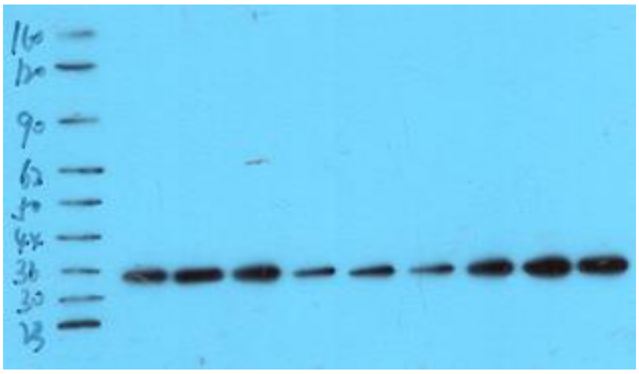

Supplementary Figure 55: Original blots presented in the Figure 7H ZO-1 (Ileum)

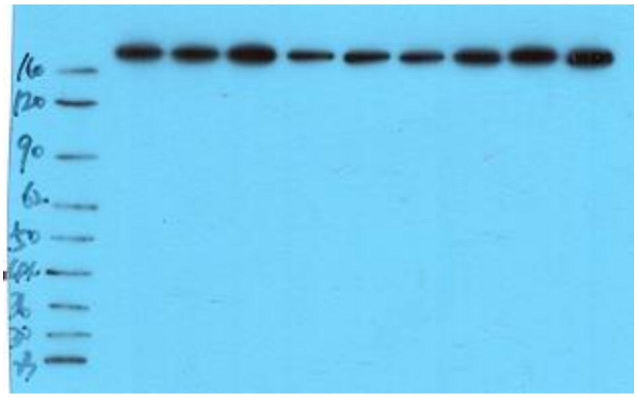

Supplementary Figure 56: Original blots presented in the Figure 7H Occludin (Ileum)

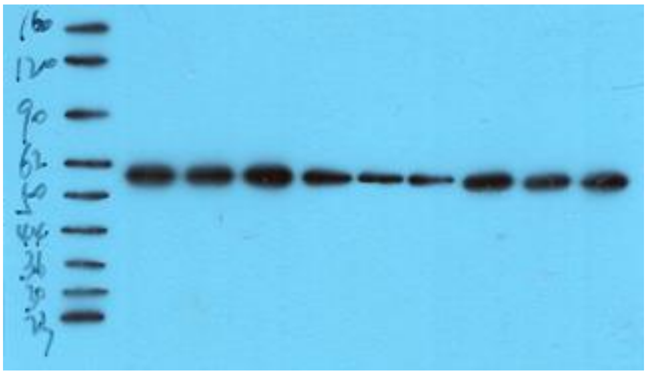

Supplementary Figure 57: Original blots presented in the Figure 7H p-AKT (Ileum)

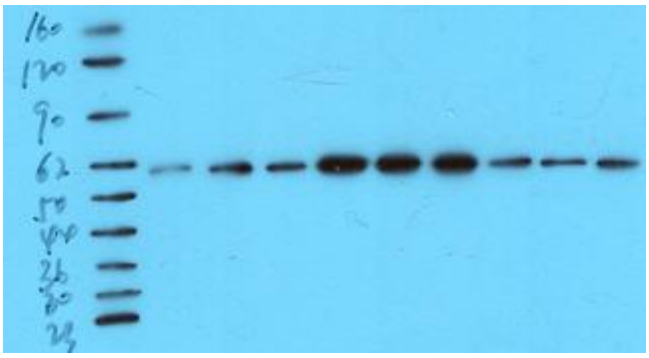

Supplementary Figure 58: Original blots presented in the Figure 7H AKT (Ileum)

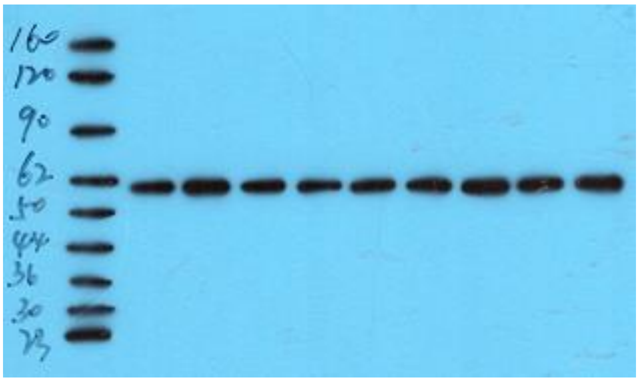

Supplementary Figure 59: Original blots presented in the Figure 7H p-NF-κB (Ileum)

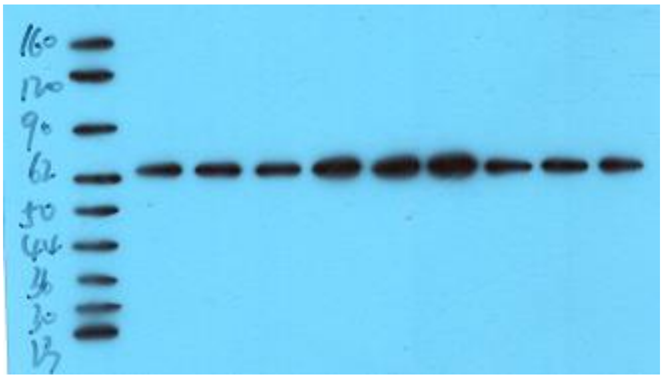

Supplementary Figure 60: Original blots presented in the Figure 7H NF-κB (Ileum)

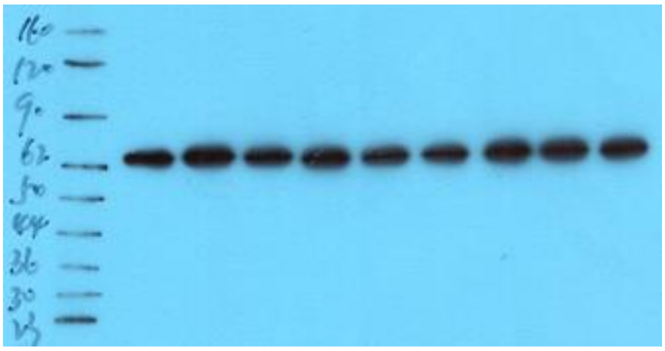

Supplementary Figure 61: Original blots presented in the Figure 7H β-actin (Ileum)

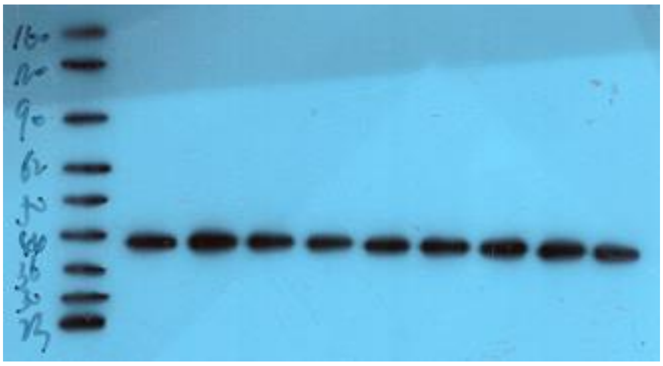

Supplementary Figure 62: Original blots presented in the Figure 7H iNOS (Colon)

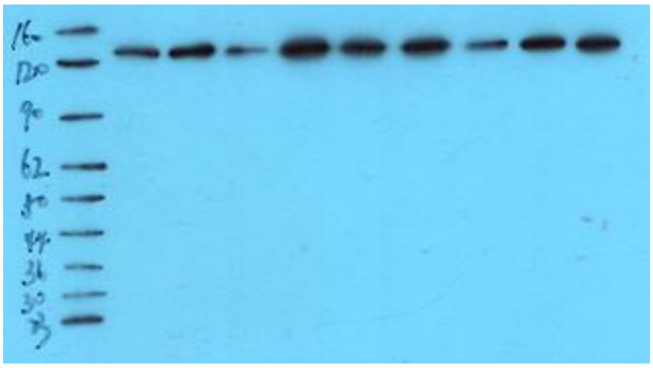

Supplementary Figure 63: Original blots presented in the Figure 7H Arg1 (Colon)

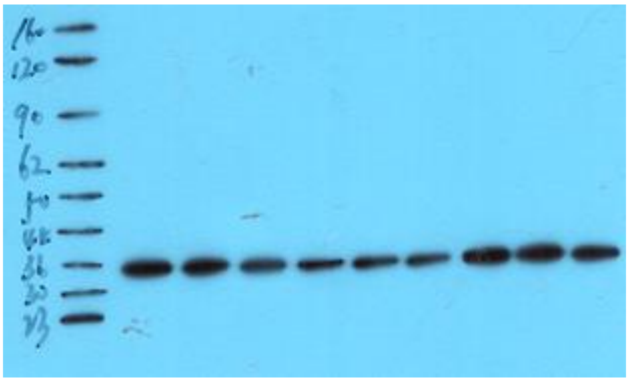

Supplementary Figure 64: Original blots presented in the Figure 7H F ZO-1 (Colon)

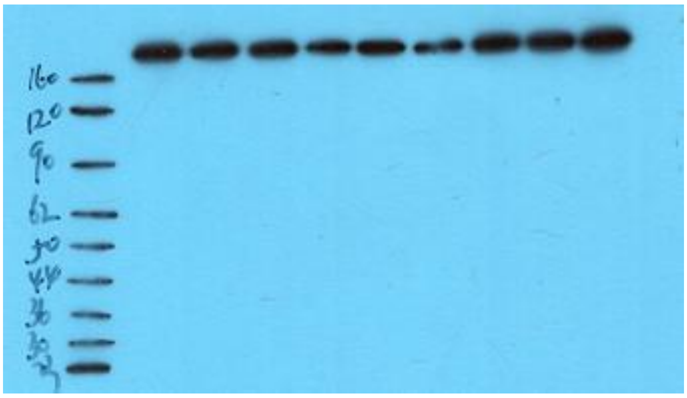

Supplementary Figure 65: Original blots presented in the Figure 7H Occludin (Colon)

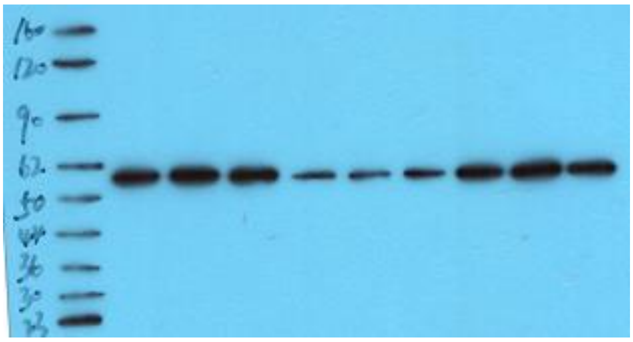

Supplementary Figure 66: Original blots presented in the Figure 7H p-AKT (Colon)

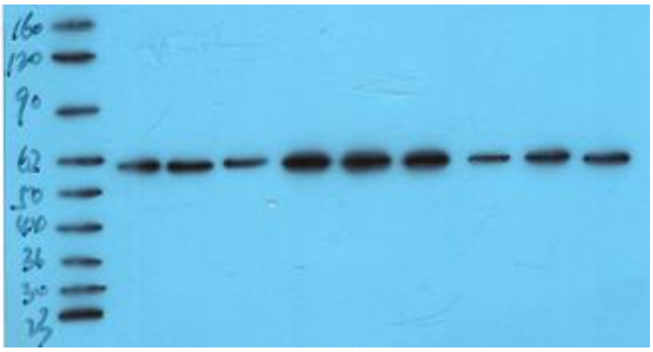

Supplementary Figure 67: Original blots presented in the Figure 7H AKT (Colon)

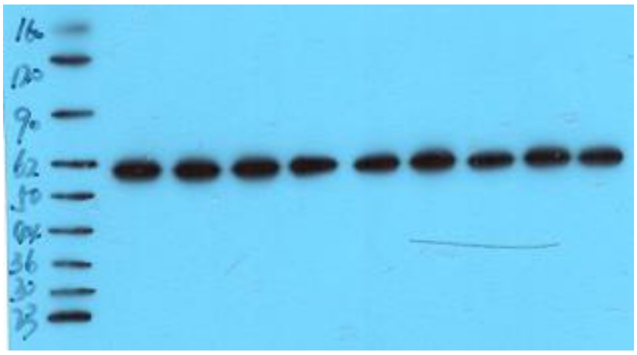

Supplementary Figure 68: Original blots presented in the Figure 7H p-NF-κB (Colon)

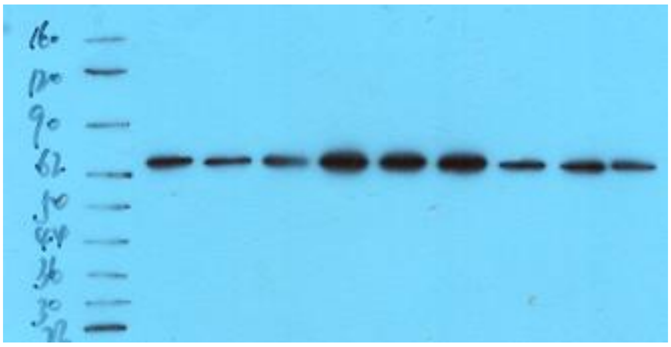

Supplementary Figure 69: Original blots presented in the Figure 7H NF-κB (Colon)

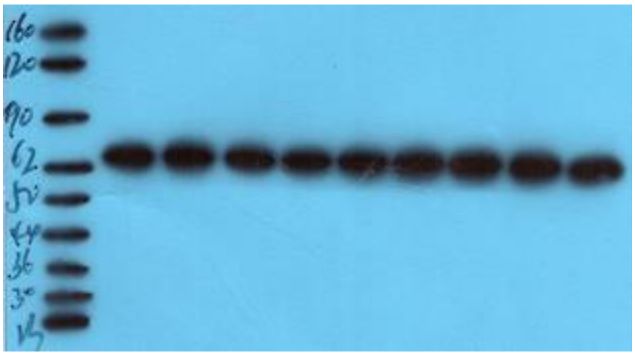

Supplementary Figure 70: Original blots presented in the Figure 7H β-actin (Colon)

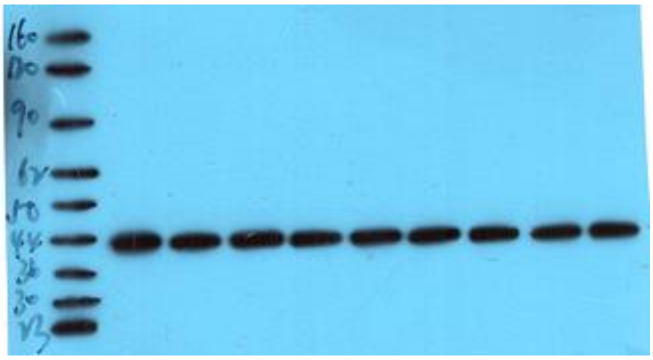

Supplement: Supplementary file 1 — Supplementary Information [file 41522_2023_403_MOESM1_ESM.pdf]
