# Supplementary material for: Limosilactobacillus mucosae-derived extracellular vesicles modulates macrophage phenotype and orchestrates gut homeostasis in a diarrheal piglet model
Source: NPJ Biofilms Microbiomes. 2023 Jun 6;9:33. doi: 10.1038/s41522-023-00403-6 (PMC10244441; doi:10.1038/s41522-023-00403-6)
Supplement: Supplementary file 2 — nr-reporting-summary [file 41522_2023_403_MOESM2_ESM.pdf]

nature portfolio

Corresponding author(s): Shiyu Tao
Last updated by author(s): 2023-5-17

Reporting Summary

Nature Portfolio wishes to improve the reproducibility of the work that we publish. This form provides structure for consistency and transparency in reporting. For further information on Nature Portfolio policies, see our Editorial Policies and the Editorial Policy Checklist.

Statistics

For all statistical analyses, confirm that the following items are present in the figure legend, table legend, main text, or Methods section.

n/a	Confirmed	
☐	☒	The exact sample size (*n*) for each experimental group/condition, given as a discrete number and unit of measurement
☐	☒	A statement on whether measurements were taken from distinct samples or whether the same sample was measured repeatedly
☐	☒	The statistical test(s) used AND whether they are one- or two-sided<br>*Only common tests should be described solely by name; describe more complex techniques in the Methods section.*
☒	☐	A description of all covariates tested
☒	☐	A description of any assumptions or corrections, such as tests of normality and adjustment for multiple comparisons
☒	☐	A full description of the statistical parameters including central tendency (e.g. means) or other basic estimates (e.g. regression coefficient) AND variation (e.g. standard deviation) or associated estimates of uncertainty (e.g. confidence intervals)
☒	☐	For null hypothesis testing, the test statistic (e.g. *F*, *t*, *r*) with confidence intervals, effect sizes, degrees of freedom and *P* value noted<br>*Give P values as exact values whenever suitable.*
☒	☐	For Bayesian analysis, information on the choice of priors and Markov chain Monte Carlo settings
☒	☐	For hierarchical and complex designs, identification of the appropriate level for tests and full reporting of outcomes
☒	☐	Estimates of effect sizes (e.g. Cohen's *d*, Pearson's *r*), indicating how they were calculated

Our web collection on statistics for biologists contains articles on many of the points above.

Software and code

Policy information about availability of computer code

Data collection	No software was used.
Data analysis	SPSS version 20.0 for Windows; SPSS Inc., Chicago, IL, USA

For manuscripts utilizing custom algorithms or software that are central to the research but not yet described in published literature, software must be made available to editors and reviewers. We strongly encourage code deposition in a community repository (e.g. GitHub). See the Nature Portfolio guidelines for submitting code & software for further information.

Data

Policy information about availability of data

All manuscripts must include a data availability statement. This statement should provide the following information, where applicable:

- Accession codes, unique identifiers, or web links for publicly available datasets
- A description of any restrictions on data availability
- For clinical datasets or third party data, please ensure that the statement adheres to our policy

Provide your data availability statement here.

Research involving human participants, their data, or biological material

Policy information about studies with human participants or human data. See also policy information about sex, gender (identity/presentation), and sexual orientation and race, ethnicity and racism.

Reporting on sex and gender	NA
Reporting on race, ethnicity, or other socially relevant groupings	NA
Population characteristics	NA
Recruitment	NA
Ethics oversight	NA

Note that full information on the approval of the study protocol must also be provided in the manuscript.

Field-specific reporting

Please select the one below that is the best fit for your research. If you are not sure, read the appropriate sections before making your selection.

☒ Life sciences ☐ Behavioural & social sciences ☐ Ecological, evolutionary & environmental sciences

For a reference copy of the document with all sections, see nature.com/documents/nr-reporting-summary-flat.pdf

Life sciences study design

All studies must disclose on these points even when the disclosure is negative.

Sample size	We did not predetermined the sample size.The number of samples/experiments for each data panel has been described in the figure legends.
Data exclusions	No data were excluded from the analyses.
Replication	Two experiments were performed. In our manuscript, results are representative of two independent experiments.
Randomization	Experimental groups were defined by experimental conditions. Within an experimental condition, the allocation was random.
Blinding	All experimental procedures and quantification of results including drug injections and tissue histological analysis were done by two independent researchers.

Reporting for specific materials, systems and methods

We require information from authors about some types of materials, experimental systems and methods used in many studies. Here, indicate whether each material, system or method listed is relevant to your study. If you are not sure if a list item applies to your research, read the appropriate section before selecting a response.

Materials & experimental systems

n/a	Involved in the study	
☐	☒	Antibodies
☐	☒	Eukaryotic cell lines
☒	☐	Palaeontology and archaeology
☐	☒	Animals and other organisms
☒	☐	Clinical data
☒	☐	Dual use research of concern
☒	☐	Plants

Methods

n/a	Involved in the study	
☒	☐	ChIP-seq
☒	☐	Flow cytometry
☒	☐	MRI-based neuroimaging

Antibodies

Antibodies used	All the antibodies used in the manuscript have a full describtion in the online methods.
Validation	All the antibodies are validated and provided on the manufacturer"s website.

Eukaryotic cell lines

Policy information about cell lines and Sex and Gender in Research

Cell line source(s)	MODE-K and IPEC-J2 cells were kindly provided by Prof. Wang of China Agricultural University (Beijing, China).
Authentication	None of the cell lines were authenticated.
Mycoplasma contamination	All cell lines tested negative for mycoplasma contamination.
Commonly misidentified lines (See ICLAC register)	NA

Animals and other research organisms

Policy information about studies involving animals; ARRIVE guidelines recommended for reporting animal research, and Sex and Gender in Research

Laboratory animals	Germ-free mice (Kunming, 4 weeks of age) and SPF mice (BALB/c, 4-6 weeks of age) were purchased from Laboratory Animal Center of the Huazhong Agricultural University (Wuhan, China).
Wild animals	The study did not involve wild animals.
Reporting on sex	The study did not involve sex.
Field-collected samples	This study did not involve samples collected from the field.
Ethics oversight	All the animal experimental and sample collection procedures were approved by the Institutional Animal Care and Use Committee of the Huazhong Agricultural University, Hubei, China. In this study, all experimental methods were performed in accordance with the Huazhong Agricultural University of Health Guide for the Care and Use of Laboratory Animals (Approval number HZAUMO-2022-0005).

Note that full information on the approval of the study protocol must also be provided in the manuscript.
